# Brood Ball-Mediated Transmission of Microbiome Members in the Dung Beetle, *Onthophagus taurus* (Coleoptera: Scarabaeidae)

**DOI:** 10.1371/journal.pone.0079061

**Published:** 2013-11-01

**Authors:** Anne M. Estes, David J. Hearn, Emilie C. Snell-Rood, Michele Feindler, Karla Feeser, Tselotie Abebe, Julie C. Dunning Hotopp, Armin P. Moczek

**Affiliations:** 1 Towson University, Department of Biological Sciences, Baltimore, Maryland, United States of America; 2 Institute for Genome Sciences, University of Maryland School of Medicine, Baltimore, Maryland, United States of America; 3 Department of Biology, Indiana University, Bloomington, Indiana, United States of America; 4 Department of Microbiology and Immunology, University of Maryland School of Medicine, Baltimore, Maryland, United States of America; 5 J. Craig Venter Institute, Inc., Plant Genomics, Rockville, Maryland, United States of America,; University Of Montana - Missoula, United States of America

## Abstract

Insects feeding on plant sap, blood, and other nutritionally incomplete diets are typically associated with mutualistic bacteria that supplement missing nutrients. Herbivorous mammal dung contains more than 86% cellulose and lacks amino acids essential for insect development and reproduction. Yet one of the most ecologically necessary and evolutionarily successful groups of beetles, the dung beetles (Scarabaeinae) feeds primarily, or exclusively, on dung. These associations suggest that dung beetles may benefit from mutualistic bacteria that provide nutrients missing from dung. The nesting behaviors of the female parent and the feeding behaviors of the larvae suggest that a microbiome could be vertically transmitted from the parental female to her offspring through the brood ball. Using sterile rearing and a combination of molecular and culture-based techniques, we examine transmission of the microbiome in the bull-headed dung beetle, *Onthophagus taurus*. Beetles were reared on autoclaved dung and the microbiome was characterized across development. A ~1425 bp region of the 16S rRNA identified Pseudomonadaceae, Enterobacteriaceae, and Comamonadaceae as the most common bacterial families across all life stages and populations, including cultured isolates from the 3^rd^ instar digestive system. Finer level phylotyping analyses based on *lepA* and *gyrB* amplicons of cultured isolates placed the isolates closest to *Enterobacter cloacae, Providencia stuartii, Pusillimonas* sp.*, Pedobacter heparinus*, and *Lysinibacillus sphaericus*. Scanning electron micrographs of brood balls constructed from sterile dung reveals secretions and microbes only in the chamber the female prepares for the egg. The use of autoclaved dung for rearing, the presence of microbes in the brood ball and offspring, and identical 16S rRNA sequences in both parent and offspring suggests that the *O. taurus* female parent transmits specific microbiome members to her offspring through the brood chamber. The transmission of the dung beetle microbiome highlights the maintenance and likely importance of this newly-characterized bacterial community.

## Introduction

Dung beetles in the superfamily Scarabaeoidea have specialized on animal waste since the Jurassic period ~152 million years ago [1,2]. As critical decomposers in all temperate and tropical terrestrial ecosystems, dung beetles have evolved to feed on both dry [3,4] and wet dung of mammals in general, and large herbivorous mammals in particular [5], on every continent except Antarctica [6,7]. While many beetles have radiated onto dung as a food source, dung is a nutritionally incomplete diet. Dung lacks amino acids essential for insect metabolic needs including tryptophan, methionine, phenylalanine, histidine, and arginine [8]. The dung of herbivorous ruminants, such as cattle, deer, and buffalo, is more than 86% cellulose [8] — an indigestible polysaccharide for many eukaryotes. Despite these attributes, members of Diptera (flies) and Coleoptera (beetles) specialize on dung.

Although it may seem surprising that such diverse insects have radiated onto a nutritionally-poor resource, mutualistic symbioses between bacteria and their eukaryotic hosts allow animals to feed on a diversity of diets that would otherwise be inaccessible to the host. Such beneficial endosymbionts can provide essential amino acids and vitamins lacking in the host diet, or they can synthesize novel enzymes, such as cellulases and hydrolases, to degrade otherwise indigestible materials like cellulose, lignin, and chitin [9,10]. These mutualisms are seen in animals ranging from cellulose feeding vertebrates to wood-, sap-, and blood-feeding insects [9]. In insects, mutualistic endosymbionts frequently supplement the host with essential amino acids and vitamins missing from their food source [10,11]. This supplementation may be provided primarily by one symbiont species, as in the aphid-*Buchnera* system [12,13], or by a community of symbionts, such as in termites [14,15]. 

No matter the number of beneficial endosymbionts, the faithful transmission of these specific mutualists from parent to offspring is essential for offspring survival. There are two broad categories of transmission: vertical transmission, where symbionts are acquired from the parent, and horizontal transmission, where they are not [9,16]. In the dung beetle system, horizontal transmission could be from many sources including adult siblings, other insect taxa including other species of dung beetles, and host dung. Vertical transmission is the dominant transmission type in evolutionarily stable, obligate insect-bacterial nutritional mutualisms. Vertically transmitted endosymbionts are frequently transmitted transovarially, though alternate methods of transmission from parents to offspring are known [9,16]. These include endosymbiont transmission through proctodeal trophallaxis [17], milk glands [18,19], coprophagy [20], egg smearing [21,22] , capsules [23], and brood chambers [24]. 

Dung beetle diversity may in part be a result of microbial inhabitants that first allowed these scarab beetles to radiate onto and exploit niches that are otherwise inaccessible [25]. Within scarab beetles, specific microbes have been identified using 16S rRNA in a handful of beetle species that feed on living plant tissue [26,27] and humus [28] instead of dung [29,30]. Earlier work on two dung-feeding scarab beetles, *Scarabaeus semipunctatus* and *Chironitis furcifer*, identified culturable dung and cellulose degrading bacteria associated with the beetles, their brood balls, and their vertebrate dung source (i.e. sheep and cow, respectively) using dung agar plates [31]. We hypothesize that species of rolling and tunneling dung beetles that sequester individual eggs in brood balls of dung may provision their brood balls with a specific microbiome to aid in dung digestion. Our research focused on a more thorough description of the dung beetle microbiome by focusing on a species of tunneling dung beetle in the genus *Onthophagus*, which processes the dung of large herbivorous mammals. 

*Onthophagus* is the most diverse tunneling dung beetle genus, with over 2,000 species described [32]. In the United States alone, there are at least 35 native *Onthophagus* spp. [32] and at least 5 more introduced, naturalized, non-native species [33–35]. All of these species are known to be dung specialists. *Onthophagus taurus*, the bull-headed dung beetle, is one of the most abundant dung beetles specialized on cattle dung. It is a native of the Mediterranean, was first found in Florida in 1974 [36], and has increased its distribution throughout the U.S. ever since [37]. Although native dung beetles such as *O. hecate* and *O. pennsylvanicus* are also found in agricultural habitats, *O. taurus* is the most abundant beetle in these settings. However, *O. taurus* is uncommon in non-agricultural ecosystems [37]. 

**Figure 1:**
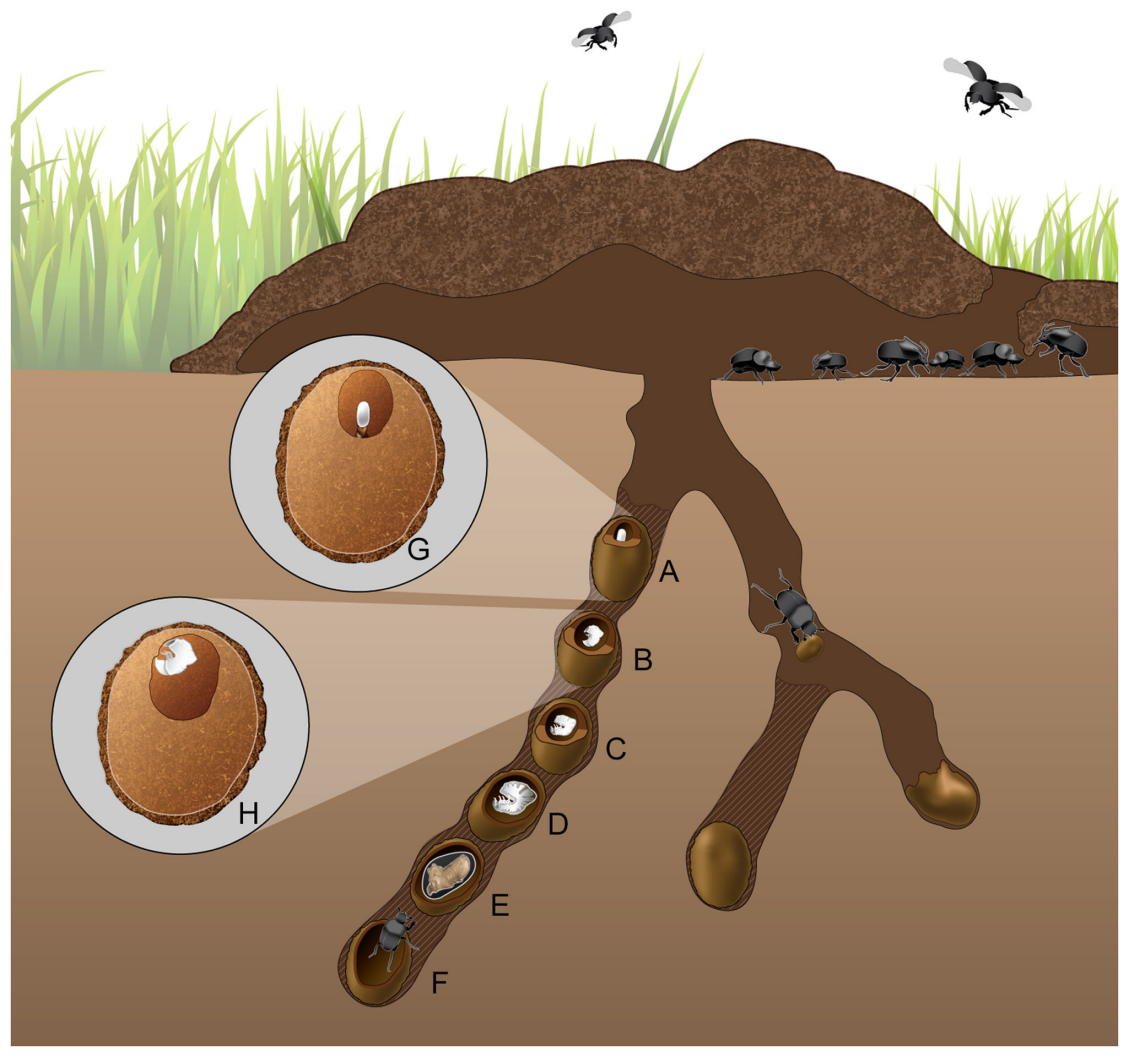
*O. taurus* life cycle. Adults fly into the dung pat to feed and mate. Beneath the dung pat, the juvenile life stages of *Onthophagus taurus* are isolated in the brood chamber of the brood balls constructed by the female beetles in tunnels. Females lay several brood balls in each tunnel that would all be at the same developmental stage. However, for illustrative purposes all life stages are represented in one tunnel. These stages include the: (A) egg, (B) 1^st^ larval instar, (C) 2^nd^ larval instar, (D) 3^rd^ larval instar, (E) pupa, and (F) an eclosing adult beetle that is tunneling toward the surface. The brood ball chamber is larger with each successive life stage as the larva feeds on the chamber walls within the brood ball. The top inset shows (G) the fecal pedestal the egg is positioned upon in brood ball. The bottom inset shows (H) the larval instar feeding on the walls of the brood ball chamber.

**Figure 2:**
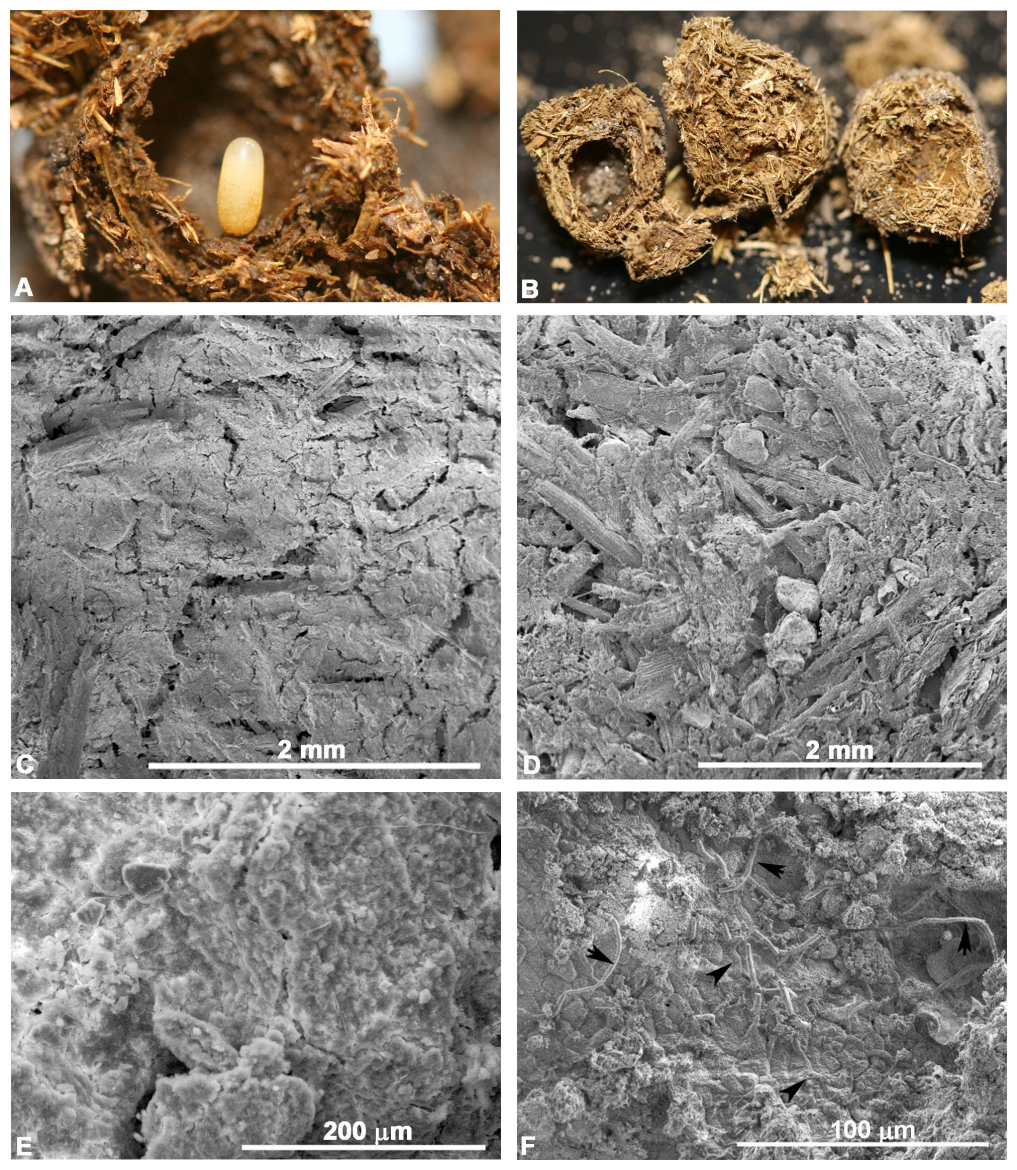
Brood ball chamber. The brood ball chamber is a unique structure for the transmitted microbiome of dung beetles. (A) The innermost walls of the brood ball chamber with the egg are smooth as compared to the fibrous outer walls. (B) A brood ball is shown broken into 3 pieces. The hollow brood ball chamber is where the egg develops (left). The remainder of the brood ball is filled with cellulose-rich, fibrous dung (center and right). Images C-F are scanning electron micrographs of different portions of the brood ball. (C) Micrograph illustrating the smooth biofilm-like matrix coating the inner wall of the brood ball chamber. (D) Fibrous bits of grass from the cow dung found in the portion of the brood ball away from the chamber. (E) Higher magnification of the smooth biofilm-like matrix that coats the brood ball chamber walls. (F) When the smooth matrix is scraped away, rod shaped microbes in chains (arrows) are found underneath the smooth matrix of the brood ball chamber walls. Other rod-shaped structures in the background remain covered in the biofilm-like matrix (arrowheads). This matrix and microbes are only observed where the mother has prepared the brood ball chamber.

*Onthophagus* adults fly to a fresh dung pad where they use scoop-like mouthparts to filter the liquid portion of the dung for associated microbes [38] (Figure 1). Females then tunnel vertically in the soil underneath the dung pad where they form a series of brood balls for their offspring. Females move dung down to the ends of the tunnel where they pack dung into an oval brood ball with a brood chamber at one end of the brood ball (Figure 1). The female meticulously constructs a brood chamber lined with her own saliva where a single egg [39] is laid on top of a pedestal made of the adult female's own excrement [31,39] (Figure 1 A and G and 2 A). The entire juvenile portion of the life cycle - all 3 larval instars as well as pupation - occurs within this brood ball chamber [31,39] (Figure 1A-H). The larva hatches from the egg. Using its heavily sclerotized, toothed mandibles [38], the first instar larva immediately feeds on the dung pedestal, and then methodically alternates feeding on the solid, cellulose-rich portion of dung of the brood ball wall and its own excrement (Figure 1H) until pupation [31] (Figure 1E). Larvae pupate inside a pupation chamber constructed out of late larval fecal matter and left-over brood ball material in the remains of the brood ball (Figure 1E). After ~ 8 days at 25 °C the pupa ecloses into the filial adult, which remains inside the pupation chamber for at least several days until fully sclerotized. During dry or winter seasons, pupae will remain in their pupation chamber until the appropriate weather conditions are present. At this point during the breeding and nesting season, the adult digs to the soil surface to find food and mate [39] (Figure 1F).

Based on the nutritionally incomplete diet dung provides, the nesting behavior of the female, and the larval feeding behavior, we hypothesize that a vertically transmitted microbiome is present that is transmitted through the brood ball in the dung beetle, *Onthophagus taurus*. Here we use a sterile rearing technique along with culture based and molecular methods to assess the potential for microbiome transmission and inheritance of the bull headed dung beetle, *O. taurus*, through the brood ball. 

## Methods

### Beetle collection and sterile rearing of offspring

Adult female *Onthophagus taurus* were collected with permission from the land owner from fresh cow manure on the private organic dairy farms of Ms. Whitney Schlegel at Marble Hill Farm in Monroe County, IN, USA, and Mr. Bob Nutter from Maple View Farm in Wake County, NC, USA. Beetles collected in IN and NC were brought into the laboratory and reared as previously described [40], but with the following modifications to eliminate contamination with bacteria from human caretakers, manure, and the laboratory. All dung, sand, soil, and water were autoclaved at 15 PSI and 121 °C for 30 min to kill living bacteria. Sterile dung was stored at -80 °C until use. Non-autoclavable items (e.g., beetle colony rearing bins) were disinfected with 1% sodium hypochlorite and detergent and then rinsed with sterile water. Gloves were used at all times by human caretakers. 

To obtain offspring, brood balls from each of two wild-collected females were harvested every 10 d and housed separately to avoid any transfer of microbiota among offspring. At least 15 brood balls were collected from each female so that offspring could be obtained at several developmental stages. Offspring were removed using sterilized forceps after the brood balls were opened with gloved hands. For the molecular based bacterial identification, the offspring were sacrificed in 95% ethanol for DNA extractions from 4 stages including 2 first instar larvae from IN, 1 second instar larva from IN, 2 second instar larvae from NC, 3 third instar larvae from IN, 3 third instar larvae from NC, and 2 pupae from IN. For culture-based work, 4 individual 3^rd^ instar larvae from the NC population were collected and dissected to remove the entire digestive system. Original wild-caught female parent beetles from NC and IN were sacrificed in 95% ethanol after their final brood ball was collected. All samples were held in ethanol at 4 °C until DNA was extracted. In total, 17 offspring (9 from NC and 8 from IN) and two wild-caught females were harvested. From each of these individuals, multiple culturing and cloning efforts were conducted to characterize the beetles’ internal microbiota.

### Scanning electron microscopy

We used scanning electron microscopy to survey brood balls for bacteria. We expected bacteria to be present on the brood ball chamber walls where the female deposited a matrix covering the walls. No bacteria were expected outside of the brood ball chamber in the remainder of the brood ball that is away from the chamber, which is composed only of dung, and lacks the matrix found in the brood ball chamber. Brood balls ~ 2-3 d old were collected from the rearing bins in the laboratory and immersed in a standard fixation solution of 2% paraformaldehyde, 2.5% gluteraldehyde and 0.1 mM cacodylate buffer containing 0.06% calcium chloride for 1 h. Samples were rinsed 3X for 15 min each rinse in the cacodylate buffer, post-fixed in 1% osmium tetroxide in cacodylate buffer, rinsed 3X in water, and subjected to an ethanol dehydration gradient ranging from 30% to 100%. Samples were held in 100% ethanol overnight at RT. Samples were then placed in a SamiDri-810 Critical Point Dryer (Tousimis) for 30 min, then broken open with gloved hands and mounted onto stubs for viewing in an FEI Quanta 200 scanning electron microscope. 

### Generation of 16S rRNA sequence data

Beetles were surface sterilized in 1% sodium hypochlorite and 0.1% Triton-X 100, followed by 2 rinses in sterilized water [21]. DNA was extracted from the egg, whole animal, or the cultured bacterial isolate with the PowerSoil Kit (MoBio, Carlsbad, CA), and ~1490 bp 16S rRNA amplicons were generated using universal primers, 10F and 1507R [41], which have been used for several studies of insect associated bacteria [42–45]. The primer analysis program TestProbe in Silva [46] showed that allowing for 2 mismatches, 10F matched ~ 26% (24725/95194) of the bacterial sequences present in the database while 1507R with 2 mismatches matched ~ 56% (63788/113979). The majority of 10F matches were to the Proteobacteria, especially γ-Proteobacteria. The reverse primer, 1507R, had matches similar to 10F, but also included Acidobacteria. As such, sequences obtained with these primers may be enriched for Proteobacteria, and more specifically γ-Proteobacteria. 

As controls, PCR was run on various additional samples to ensure that sterile conditions were maintained. To ensure that surface sterilization removed all bacteria, 400 µl of the rinse water was subjected to a DNA extraction. DNA was also extracted from three samples of 0.25 g of fresh, frozen, or autoclaved dung and autoclaved soil. Wash water, dung, and soil DNA extractions were templates for 16S rRNA PCR as described for the beetle samples. DNA from the olive fruit fly, *Bactrocera oleae*, and its endosymbiont which amplifies with the primers 10F-1507R, were used as a positive control. Amplifications were conducted in a Mastercycler pro (Eppendorf, Hamburg, Germany) using a 65-55 °C touchdown program described previously [21]. PCR amplicons were purified using the Gene JET PCR purification kit (Fermentas, Glen Burnie, MD, USA). Purified PCR amplicons were cloned into the pJET1.2/blunt plasmid (Fermentas, Glen Burnie, MD, USA) and inserted into chemically competent 10G *E. cloni®* cells (Lucigen, Middleton, WI, USA). Transformed cells were grown overnight at 37 °C in LB media with 100 mg/ml ampicillin. Between 15 and 25 colonies/beetle individual were transferred to a new plate and grown overnight in liquid media to be preserved at -80 °C in a final concentration of 40% glycerol. Colonies cloned from 16S rRNA PCR product were prepared for sequencing using either GeneJet plasmid mini prep, (Fermentas, Glen Burnie, MD, USA) of the transformed cells or from PCR products using pJET1.2F and pJET1.2R sequencing primers (Fermentas, Glen Burnie, MD, USA). Cloned samples were sequenced using primers pJET1.2F and pJET1.2R on an ABI 3730xl by Functional Biosciences (Madison, WI, USA). Sequences were edited and contigs assembled using Phred/Phrap/Consed [47]. 

### Culturing the microbiome

The entire digestive system of surface sterilized third instar larvae from NC female parent 2 (n=4) was dissected into 1 ml of sterile deionized water and homogenized using a plastic pestle in a 1.5 ml tube (USA Scientific, Orlando, FL). Serial dilutions of the homogenate were plated onto full strength and 1/10^th^ strength LB agar plates. Plates were incubated at 37 °C for 24 h. Individual colonies were picked and streaked for single colonies. Cells were grown up into either full or 1/10^th^ strength liquid LB, DNA was extracted as described for the beetle samples, and glycerol stocks were made. A total of 50 isolates were collected from 4 larval siblings of NC female parent 2. Cultured isolates were initially classified to family using 1425 bp of the 16S rRNA locus in CloVR as described below. Since 16S rRNA is known to have limited information for taxonomic resolution below the family level [48–50], the taxonomic identity of isolates that produced an amplicon with the *lepA* (~900 bp) and/or *gyrB* (~1300 bp) primers were resolved to genus or species using a phylogenomic analysis as described below.

### Microbiome community diversity analysis using 16S rRNA

The CloVR pipeline with chimera check [51] was run on 275 sequences for a 1425 bp amplicon of the 16S rRNA gene. CloVR uses scripts from Qiime [52] to cluster reads into operational taxonomic units (OTUs), select representative sequences from each OTU, and use the Ribosomal Database naïve Bayesian classifier (0.5 confidence interval) to assign each OTU to a known taxon [53]. The nucleotide sequence identity within an OTU is 95%. OTU’s (n=22) that had less than an 80% confidence with RDP were not classified [54,55]. CloVR uses scripts from Mothur [56] to plot rarefaction curves of richness and diversity, which were calculated every five sequences [53,57]. 

To assess the relatedness of 16S rRNA sequences, sequences were aligned with MUSCLE v3.7 [58], visualized and trimmed with Bioedit v7.1.3.0 [59], and a dendrogram generated with RAxML v7.3.0 [60] using a rapid Bootstrap analysis and search for best-scoring ML tree in a single run, a GTRGAMMA substitution model, random seeds of 12345, and 100 bootstraps. The best tree as ascertained by RAxML is shown and is decorated with symbols denoting the key characteristics of the sample. Percent similarity was determined between all sequences (Table S3).

### Cultured microbiome community diversity analysis using *lepA/gyrB* phylotyping

To compare the microbiome community composition of the cultured isolates to the microbiome community found using molecular methods, all cultured isolates were screened using previously described degenerate primer sets designed for 10 single copy loci. The *lepA* (lepABAUP2/lepABNDN1) and *gyrB* (gyrBBAUP2/gyrsBBNDN1) primer pairs [48] amplified the majority of the cultured isolates. Cultured isolates that amplified with the *lepA* or *gyrB* primers were classified to their most specific taxonomic ranking in a phylogenomic context using a modified version of the AMPHORA pipeline [61]. A maximum likelihood phylogenomic tree was constructed of 1095 complete bacterial genomes (Table S1) based on 264 protein-coding loci concatenated into a supermatrix (Dryad http://doi.org/10.5061/dryad.266qs), Table S2). This tree was then used as a constraint tree to place the *lepA/gyrB* amplicons sequenced from cultured bacteria isolated from the dung beetle larval digestive system. For the constrained analysis, the concatenated *lepA*/*gyrB* sequences from the complete and annotated genomes were analyzed along with the *lepA*/*gyrB* sequences from the dung beetle microbial isolates.

### Nucleotide sequence accession numbers

The 296 sequences generated during this study were deposited in GenBank (Accession numbers: 16S rRNA from cultured isolates: KF193090-KF193120; 16S rRNA from cloned isolates: KF193120-KF193354; *gyrB* from cultured isolates: KF193355-KF193369; *lepA* from cultured isolates: KF193370-KF193385). Phylogenetic trees and amino acid alignments used for phylogenetic inference have been deposited in Dryad http://doi.org/10.5061/dryad.266qs.

## Results

### Sterile rearing of offspring

In order to assess the transmission pattern of the microbiome, dung beetles needed to be both surface sterilized and reared aseptically. Beetle offspring were surface sterilized by washing with dilute bleach and detergent followed by two water rinses. No 16S rRNA PCR amplicons were observed for DNA extracted from the rinse water from surface sterilized beetles (Figure S1, Lane RW). Under the same conditions, DNA extracted from *Bactrocera oleae*, the olive fruit fly, which is known to have bacterial symbionts that amplify with the primer pair 10F-1507R [21,62] yielded the ~1500 bp amplicon and served as a positive control (Figure S1, Lane +). The negative control with sterile water as the template did not produce an amplicon (Figure S1, Lane -). This experiment was repeated two additional times with the same results.

To ensure that bacteria were not living or that exogenous DNA could not be amplified from the dung, DNA was extracted from both frozen and autoclaved dung. The ~1500-bp 16S rRNA amplicon could not be obtained from either the frozen or autoclaved dung (Figure S1, Lane FD and AD). When the *Bactrocera oleae* positive control was added to these same negative control samples, a 1490 bp product was formed demonstrating that the lack of amplification was not due to the differential presence of inhibitors of the PCR reaction (Figure S1, Lane AD+ and AD++). No amplicons were formed from the DNA of the autoclaved water or autoclaved soil (Figure S1, Lanes AW and AS). Amplicons were produced in fresh, unautoclaved, unfrozen dung (Figure S1, Lane UG) and the DNA extracted from the homogenized digestive system of the 3^rd^ larval instar (Figure S1, lane Gut). This experiment was repeated two additional times with the same results.

### Scanning electron microscopy

The inner walls of the brood ball chamber were covered in a matrix making an extremely smooth lining of the chamber walls (Figure 2 A, B, C, and E), especially compared to the portions of the brood ball away from the brood ball chamber that were composed entirely of fibrous, cellulose-rich dung (Figure 2B and D). In regions where the matrix covering the brood ball chamber walls was scraped away, clusters of rod-shaped structures resembling large microbes were better observed (Figure 2F). Rod-shaped structures ranged in width from 1.79 to 2.83 µm (average of 2.10 µm; N=10) and lengths of individual rods ranging from 5.82 to 18.3 µm (average of 11.72 µm; N=10). Some rods formed chains (Figure 2F). These rod-shaped microbes are found sandwiched in a smooth matrix that covers the brood ball chamber walls and away from the fibrous component of the brood ball wall. Microbes and the smooth matrix were not found in our screening of the portions of the brood ball away from the brood ball chamber. Instead, only pieces of grass were seen (Figure 2B and D). No microbes were seen on the surface of the egg. Additionally, 11 eggs from a total of 8 different females did not produce amplicons using the 16S rRNA PCR primers.

### Identification of the *O. taurus* microbiome using molecular methods.

**Figure 3:**
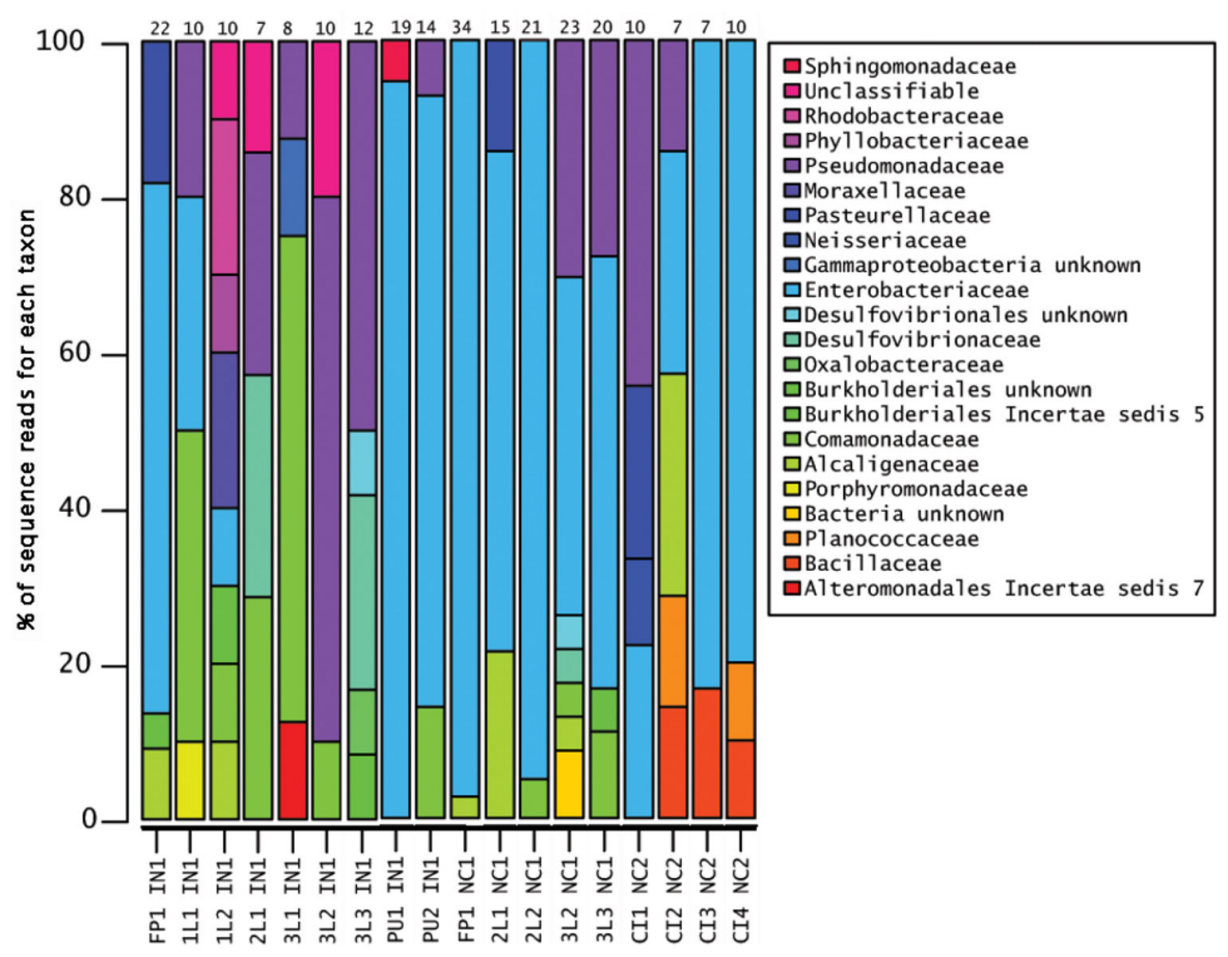
Relative abundance of family level OTUs composing the dung beetle microbiome. The relative abundance of each taxon is shown for each sample. Enterobacteriaceae (bright blue) were found in 95% of the samples, in all populations, and all life stages. Other major components include bacteria in the Comamonadaceae (green) and Pseudomonadaceae (bluish purple). Sample names are abbreviated as follows: 1L (1^st^ instar larva), 2L (2^nd^ instar larva), 3L (3^rd^ instar larva), PU (pupa), CI (cultured isolates), FP (female parent), IN (Indiana population), and NC (North Carolina population). The number after the life stage indicates different individuals sampled.

**Figure 4:**
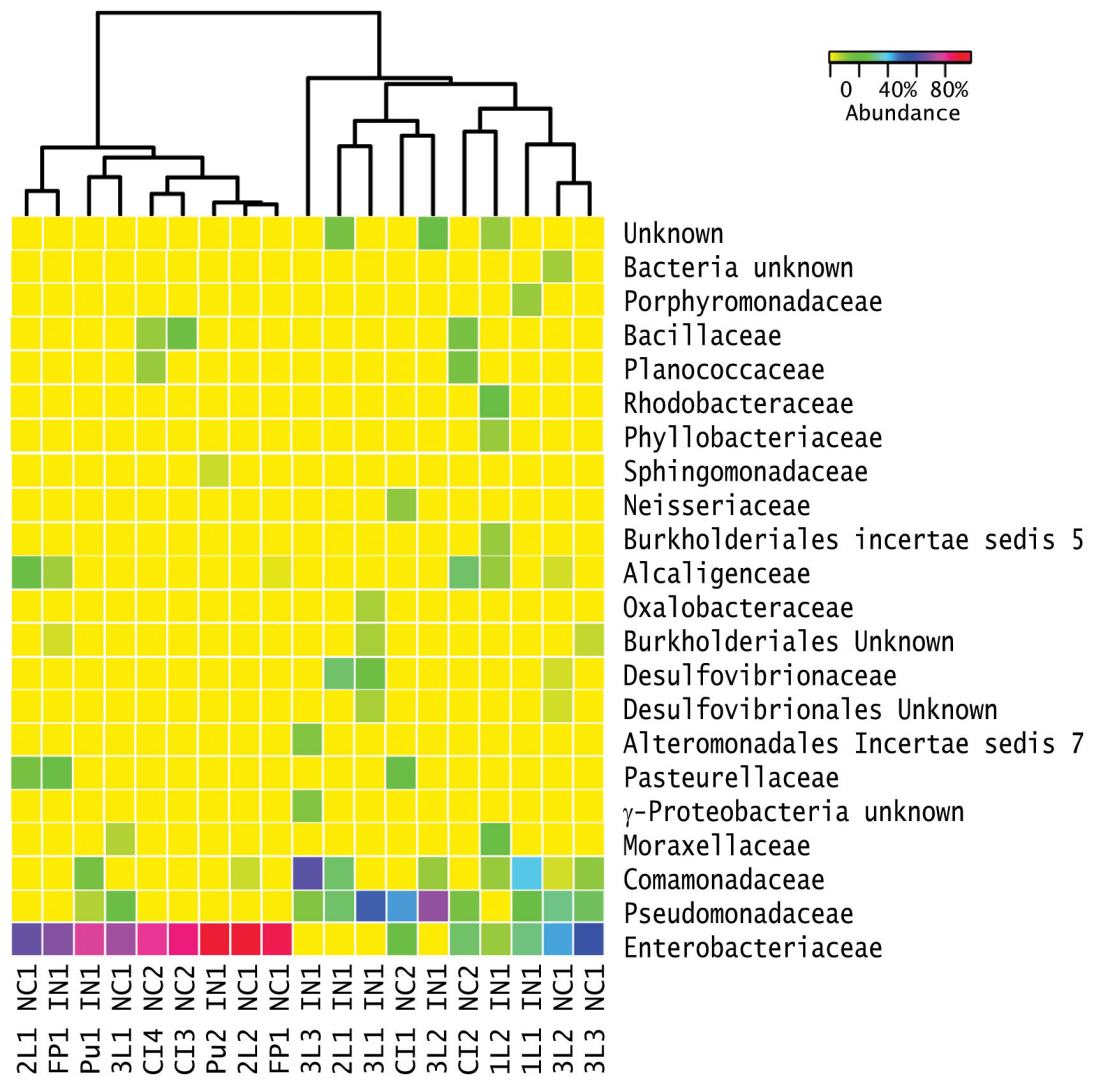
Clustering of samples based on microbiome composition. The relative proportion of sequences in a specific OTU (color key in upper right corner) is illustrated on a heat map with samples clustered using a furthest neighbor clustering to determine similarity of both the composition and abundance of the microbiomes. Several OTUs were only seen once. Overall, samples did not cluster by population, parental female, life stage, or brood ball age except the cultured isolates (CI3NC1 and CI4NC1) from the digestive system of the 3^rd^ instar larvae. Interestingly, the four cultured samples were oviposited at the same time; however, samples CI3NC1 and CI4NC1 that cluster were sampled 7 d later than CI1NC1 and CI2NC1. Thus, samples CI3NC1 and CI4NC1 were older and were transitioning from the 3^rd^ larval instar to the pupal stages. Each sample is labeled by the source (CI=cultured isolate, 1L=1^st^ instar, 3L=3^rd^ instar, 2L=2^nd^ instar, Pu=pupae, FP= female parent), the isolate or individual animal number, the US state from which the parental female was isolated (NC =North Carolina populations, USA, and IN=Indiana, USA), and the female parent number (NC1, NC2, IN1).

**Figure 5:**
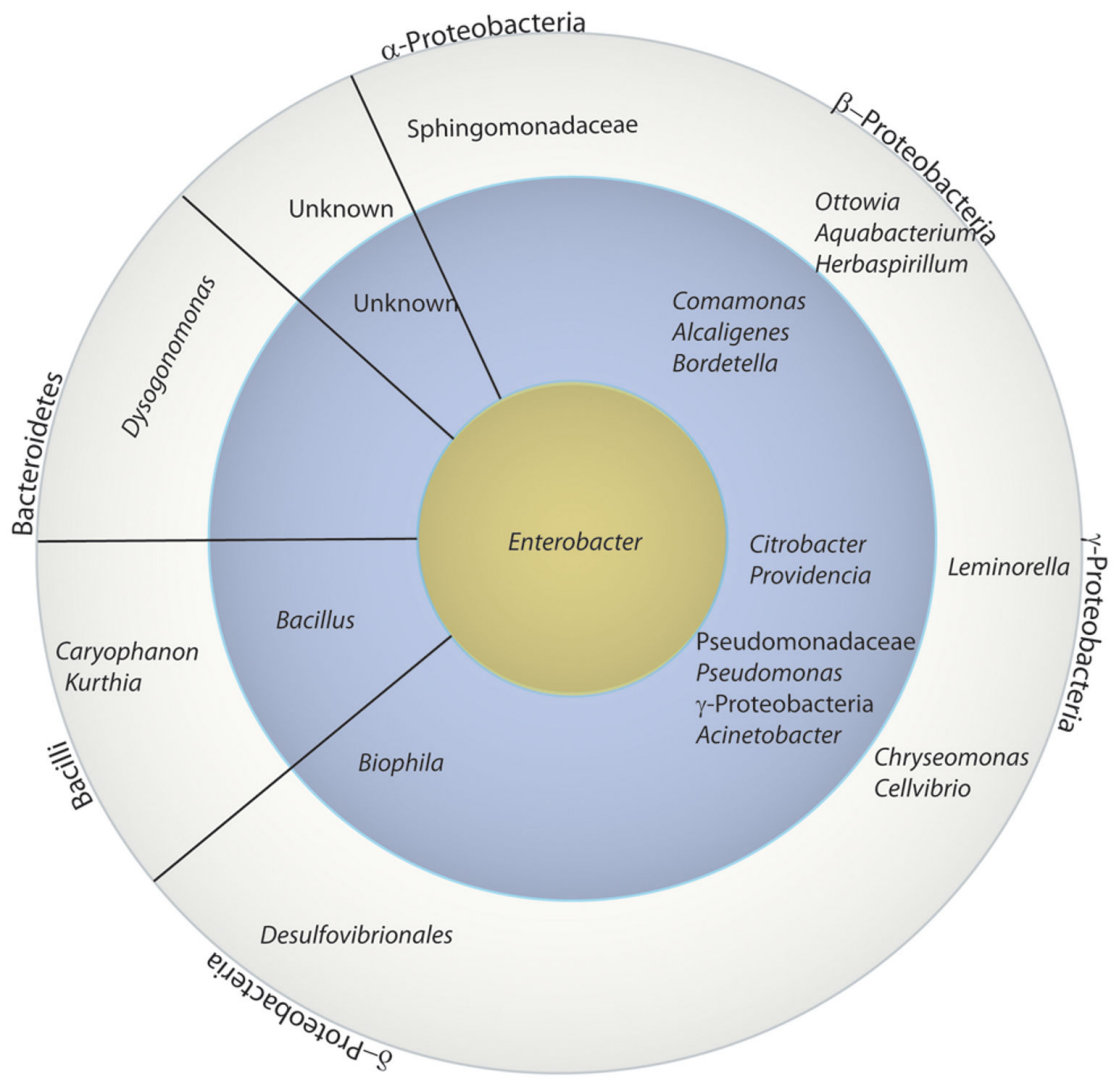
Abundance of lowest common ancestors across all microbiome samples. Bacterial OTUs identified in Qiime are arranged in a bulls-eye configuration with the most commonly occurring group in the yellow center, those occurring in two or more individuals in the blue circle, and those genera occurring only one time in the outermost rim. The lowest common ancestor Qiime classification is given and may be at the genus, family, or class level and as such may overlap. *Enterobacter* was found in offspring of all parental females and in 68% of the individuals. Samples denoted with a star are those OTUs that were found using both culture-dependent and -independent techniques.

OTUs were assigned using the CloVR pipeline with a 95% threshold as described in the methods for the 275 16S rRNA sequences obtained. Predicted chimeric sequences (n=4) as well as sequences shorter than 470 bp (n=6) were discarded. From the 265 remaining sequences, 71 OTUs were identified across 19 individuals. Of those 71 OTUs, 49 OTUs were classified to the family level, 4 to the phylum level, and 4 could not be classified further than “root” (Figures 3, 4, and 5). The most abundant genus found across all samples was *Enterobacter* (Figures 3, 4, and 5)*.*

**Figure 6:**
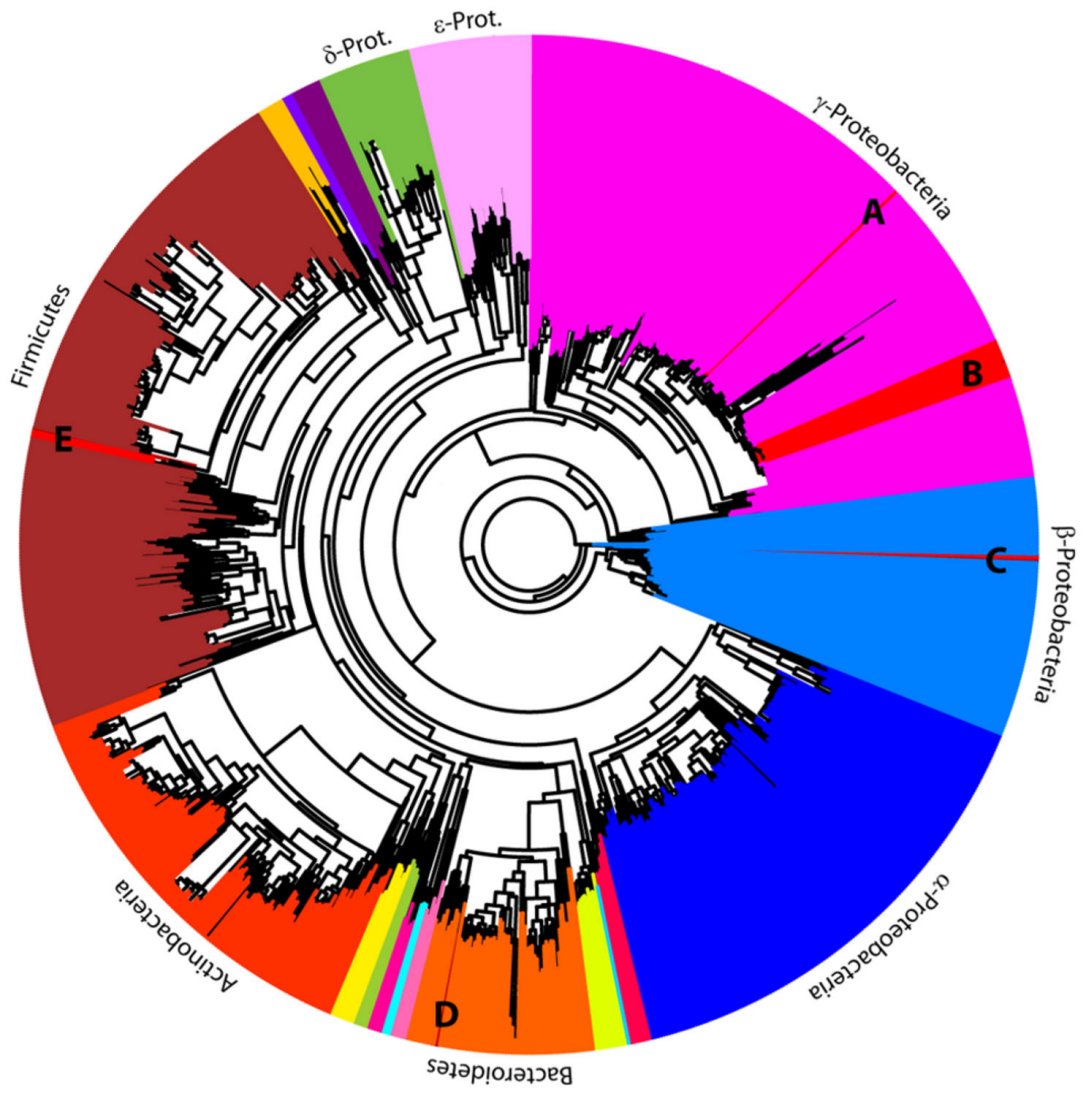
hylotyping. An unrooted phylogenomic tree based on 287 loci from 1095 sequenced bacterial genomes was made as a skeletal tree on which was placed the *lepA* and *gyrB* sequences from 16 bacterial cultures isolated from the digestive system of four different 3^rd^ instar larvae from female parent 2 from the North Carolina population. This analysis placed the isolates sister to (A) *Providencia stuartii*, (B) *Enterobacter cloacae*, (C) *Pusillimonas*, (D) *Pedobacter heparinus*, and (E) *Lysinibacillus sphaericus*.

The 16S rRNA microbial profiles were clustered into two categories using a Euclidean distance metric and complete-linkage (furthest neighbor) clustering as implemented in CloVR (Figure 4). The first cluster was dominated by Enterobacteriaceae. We detected Enterobacteriaceae in ~74% of the individuals from all larvae and pupae, the two mothers, and from both populations. The *gyrB* and *lepA* loci used for the phylotyping indicated that the most abundant culturable bacterium from our samples was most sister to *Enterobacter cloacae* within the Enterobacteriaceae (Figures 6 and S2). The second cluster was more diverse with sequences from the Comamonadaceae, Pseudomonadaceae and Enterobacteriaceae families (Figure 4). A Pseudomonadaceae was abundant in only third larval instars (Figures 3 and 4). We detected it in 42% of individuals sampled at this life stage from both NC and IN populations from either whole animals or cultured gut isolates. An unknown γ-proteobacteria (n=5) was detected in 26% of the offspring from the IN population (Figure 4). An unknown Burkholderiales (n=4) was found in both third instar larvae and one of the parental females (Figure 4). Other OTUs that were found at least once in life stages in both populations included: *Comamonas*, *Acinetobacter*, and *Bilophila* (Figure 5). 

Several offspring and their female parents have bacteria with 100% identical 16S rRNA sequencing, suggesting vertical transmission through the brood ball and its contents (Figure S3 and Table S3). For example in the North Carolina samples, one sequence was recovered five times from the female parent, four times from the 2^nd^ instar larva, and five times from the 3^rd^ instar larvae (Table S3). Additionally, OTU’s classified as *Alcaligenes* and *Pasteurella* are similar between one of the female parents and the cultured isolates from a different female parent. Deeper sequencing of individuals may reveal more shared bacterial OTUs.

Rarefaction curves of the microbial community in several larvae and the IN parental female beetle suggests that additional sampling was needed to identify additional taxa in the microbiome (Figure S4). The rarefaction curves of the NC parental female beetle (35 sequenced clones) and of a second larval instar (20 sequenced clones) suggests that microbiome taxonomic sampling to be close to saturation (Figure S4). The highest bacterial taxonomic richness was isolated from the larval samples and the IN parental female, and lower richness was sampled in pupae and in the NC parental female. However, different primer sets and deeper sampling may amplify a wider diversity of bacterial taxa. 

### Identification of the *O. taurus* microbiome using culture based methods.

The nesting activities of the female parent and the feeding behaviors of the larva suggested that the microbiome may be vertically transmitted from the female parent via the brood ball chamber. Since the microbiome resides outside of its eukaryotic host during this time, we also expected the majority of the dung beetle microbiome could be cultured. The ability to culture members of the microbiome allowed for additional taxonomic resolution of some cultured isolates. Protein coding genes may contain more informative sites allowing resolution of taxonomic identity to species and strain level better than 16S rRNA [48–50]. The majority of the cultured isolates were Enterobacteriaceae based on both the CloVR pipeline analysis of 16S rRNA (Figures 3 and 4) from the cultured isolates and the phylotyping analysis using *lepA/gyrB* (Figure 6). Members of Bacillaceae and Alcaligenaceae were also identified by both methods. Planococcaceae and Pseudomonadaceae cultured isolates only amplified with the 16S rRNA primers (Figure 4). The *lepA/gyrB* phylotyping identified an isolate in the Sphingobacteriaceae, more specifically as sister to *Pedobacter heparinus* (Figures 6 and S2). The majority of isolates in the Enterobacteriaceae were placed as sister to *Enterobacter cloaceae* by the phylotyping analysis. A second enteric isolate was identified as *Providencia* by CloVR, whereas phylotyping resolved the identity most closely to *Providencia stuartii*. In the Bacillaceae, phylotyping identified two samples as sister to *Lysinibacillus sphaericus*. Both CloVR and phylotyping identified isolates in the Alcaligenaceae, with 16S rRNA identifying an *Alcaligenes* and *lepA/gyrB* identifying an isolate as sister to *Pusillimonas* (Figures 4, 6, and S2). However, a complete *Alcaligenes* genome was not available in GenBank at the time of sampling and thus was not included in the taxonomic sampling for the phylotyping. Overall, similar taxonomic composition is seen using both methods, and, as expected, the phylotyping method often provided more finely resolved taxonomic affiliations than the CloVR analyses. Several of the cultured isolates were classified using 16S rRNA as the same OTU as sequences from larvae or the adult beetles. These include *Alcaligenes*, *Citrobacter*, *Pasteurella*, Pseudomonadaceae, and Enterobacteriaceae (Figures 3 and 4).

## Discussion

### A potentially vertically transmitted digestive system microbiome

Our experimental design involved females being provided with autoclaved dung and soil so that offspring developed in a sterile environment. Two lines of evidence suggest that the microbiome is vertically transmitted in this system. First, microbial DNA was amplified from beetles reared in sterile conditions, but not from the sterilized dung in which the female parent beetles built their brood balls. We could not produce PCR amplicons from the water surface-sterilized beetles were rinsed in, from frozen or autoclaved dung, or from autoclaved soil. Thus, it is unlikely that the dung we provided the larvae was the source of their microbiome. Second, the brood ball is the only source for a microbiome. After an egg is deposited in the brood ball, the mother has no further contact with the developing offspring. Siblings are not a potential source of microbes, as they have no contact with each other until after they emerge from the brood balls as adults. In contrast, the female beetle constructs a brood ball through chewing, regurgitating, and defecating the cattle dung, so her digestive system microbiome could be present in the brood ball. Supporting maternal-offspring transmission, a female parent and several offspring from the same population have identical 16S rRNA sequences from the microbiome (Figure S3 and Table S3). Moreover, the Enterobacteriaceae were found in 95% of the samples, in all populations, and all life stages. The Comamonadaceae and Pseudomonadaceae were other dominant members. A complete overlap in the parental and offspring taxa was not seen. However, this is not unexpected given that the offspring physiology, anatomy, and diet may enrich for members of the microbiome that are not necessarily the dominant members in the female parent. 

These initial results suggest that deeper sequencing of individuals may reveal more shared bacterial OTUs. The primers used are biased for amplifying γ-Proteobacteria, thus additional taxa may be identified using different primers. Lastly, some bacteria from the dung also may have a role in the microbiome of beetles in the wild. While, it is not a subject of this study such studies may be fruitful.

The behavior of the female parent and larval dung beetles suggests that the microbiome is vertically transmitted through structures in the brood ball or the egg itself. First, a single egg is laid on a pedestal of the female’s own excrement, or frass. The larva ecloses and immediately feeds on the pedestal. Second, the larvae then systematically alternate between feeding on the walls of the egg chamber and their own frass [31]. Many dung beetle researchers have considered this second stage of coprophagy (self-coprophagy) to be a method for further extracting the nutrients from their frass. However, we hypothesize that the larva may acquire their microbiome from the pedestal and brood ball walls. During self-coprophagy, the larva may be selecting for or concentrating the microbes that facilitate their digestion of the dung the female provides. Similarly, in other cellulose degrading taxa, such as wood roaches, juveniles do not survive unless they ingest frass from parents that have the microbiota needed for digestion [63]. No microbes or matrix were seen on the surface of the dung beetle egg. PCR amplicons were not produced when DNA from the egg was used as a template. Lack of amplification is never a definitive result because it can be explained in other ways such as lack of abundance. However, when paired with the SEM observations this suggests that egg smearing is not likely to be the mechanism of transmission. 

How the microbiome survives and is maintained during metamorphosis remains unknown. The pupae sampled had less diversity of bacterial OTUs identified than other life stages (Figures 3 and 4), although that should not be confused with abundance, which was not assessed. A similar phenomenon is seen in some holometabolous insects [21,64,65], but not others [66]. However, in those systems, the bacteria that persist through metamorphosis are those that are thought to be essential to the symbiosis [66]. Alternatively, the microbiome may be reacquired from the puparium, meconium, or other structures the recently eclosed insect feeds upon post-emergence. Examining how symbionts are maintained during metamorphosis in holometabolous insects remains an intriguing focus of future research. 

Scanning electron microscopy reveals rod-shaped structures embedded in the biofilm-like matrix of the brood ball chamber wall (Figure 2F), but lacking in the portions of the brood ball away from the chamber (Figure 2B and 2D). These rod-shaped structures were patchily distributed in the brood ball chamber walls (Figure 2F) and were best visualized when the matrix was scraped away from the chamber walls, supporting the idea that the larval self-coprophagy is necessary for increasing the microbiome population size or selecting for microbes that degrade the dung. Rod-shaped microbes could be seen within a biofilm-like matrix (Figure 2F). Although these microbes are large for many known bacterial cells, they are similar in size and form chains like *Bacillus megaterium* does. However, at this time, we cannot definitively determine if the microbes are fungal or bacterial. Whether the biofilm-like matrix was produced by the host insect, the bacteria, or both, also remains to be determined.

Collectively, the sterile rearing conditions, overlap of the microbiome between female parent and offspring, specialized dung processing behavior, and bacterial colonies found in the matrix on the brood ball chamber walls, but absent from other locations in the brood ball, suggest that the *O. taurus* microbiome is maternally transmitted via the brood ball. 

The transmission from mother through the brood ball to offspring may be essential for provisioning specific beetle endosymbionts to the offspring since dung beetles develop in an environment rich with other microbes that were excreted from the digestive tract of the mammalian host, as well as bacteria from the soil. Thus, the brood ball provides offspring not only with a safe refuge and food, but it is likely that the female parent additionally provisions a microbiome alongside the egg. However, future experimental validation is needed to further test this hypothesis. 

Vertical transmission via the brood ball may favor organisms that are culturable on standard microbiological media, as the bacteria must persist in an external environment prior to ingestion by the larvae. Our analyses based on separate loci for the cloning versus culture-based assays reveals shared bacterial taxa, suggesting a portion of the microbiome is culturable. This is in contrast to relative unculturability of the majority of vertically transmitted endosymbionts that have been studied thus far in insects. 

Using almost the entire 16S rRNA amplicon for taxonomic identification allows for increased accuracy in taxonomic assignment [67]. Additionally, studies have shown that increasing sampling depth is not necessary when microbial communities are distinct [68]. Our taxon rarefaction curves (Figure S4) indicate that further sampling is required to fully characterize the dung beetle microbiota, despite that our sampling of the cultured isolates from the digestive system of the 3^rd^ instar larvae captures much of the taxonomic variation seen using molecular approaches. However, on-going studies using next generation sequencing technologies to generate 16S rRNA profiles to increase sampling depth within individuals and additional culturing on a wider diversity of media and conditions will resolve this issue. 

To our knowledge, only one other ground-dwelling insect has been described as provisioning offspring brood chambers with specific bacterial endosymbionts. Females in the beewolf genus, *Philanthus*, smear the larval brood chambers with the actinomycete, *Candidatus* Streptomyces *philanthi*, which produces antimicrobial compounds to protect the larvae and developing pupae from soil fungi and bacteria [24,69]. As in the bull-headed dung beetle, the beewolf offspring develop underground in individual brood cells the female creates and provisions with both food and endosymbionts. Upon hatching from the egg, the larva takes up the actinomycete endosymbiont. Prior to pupation, the larva smears the endosymbiont in the cocoon and takes it up after eclosion [24]. In the case of the beewolf, the endosymbionts are beneficial, protecting the developing offspring from fungal and bacterial pathogens [24,69]. Examining other ground and dung nesting insects may identify additional insect-bacterial mutualisms. 

### Taxonomic classification of the microbiome

The overall microbiome composition across the different locales, females, and life stages could be classified into two groups. Individuals either had microbiomes dominated by Enterobacteriaceae or microbiomes composed of bacteria from Enterobacteriaceae, Pseudomonadaceae, and Comamonadaceae. Additionally, unknown γ-proteobacteria and unknown Burkholderiales were present in several individuals. The function and importance of these different bacterial taxa to the microbiome community and insect host cannot be assessed with our current data and are the focus of future studies. Both culture based and molecular techniques to characterize the dung beetle microbiome provided similar results, though the phylogenomic analysis using *lepA* and *gyrB* classified isolates to a more specific taxonomic level. 

The most abundant microbe identified was closely related to *Enterobacter cloacae. Enterobacter* are often found associated with insect digestive systems [21,70]. Different *Enterobacter* *sp.* have a diversity of functional capabilities in insect guts from digesting cellulose in *Bombyx mori* [71] to fixing nitrogen in tephritid fruit flies [70,72]. Another abundant microbe was an uncharacterized and uncultured γ-proteobacteria. Whether this bacterium is widespread in *O. taurus* or is present in other dung beetles remains to be seen. The next most abundant microbes were pseudomonads and comamonads. Pseudomonads are frequently found associated with insects where they assist in cellulose degradation. Pseudomonads have been found associating with detritivorous insects such as the rove beetle *Paederus* *sp.* (Coleoptera:Staphylinidae) [73] as well as plant-feeding beetles such as the Colorado potato beetle, *Leptinotarsa decemlineata*, (Coleoptera:Chrysomelidae) [74]. Cellulolytic pseudomonads dominated the microbiome of plant-feeding *Holotrichia parallelea* (Coleoptera:Scarabaeidae) [75]. Determining if the pseudomonads and other bacteria isolated from *O. taurus* have cellulose degrading properties is underway. If the endosymbionts of *O. taurus* have cellulose-degrading properties and are culturable, they may be bacterial isolates that could be used for processing cellulosic biofuel [75–77].

## Conclusions

Our data suggest that the dung beetle, *O. taurus*, has endosymbionts that are vertically transmitted in a unique method – via a brood ball. Several of the endosymbionts are culturable. Due to the manipulability of the system, the dung beetle symbiosis may be an ideal system to understand how host and microbes co-operate, and perhaps co-evolve, to thrive on nutritionally unbalanced diets. Additionally, these data suggest that the essential ecosystem function of dung beetles, the degradation of cellulose-rich dung, may be due to communities of bacterial endosymbionts. Whether other dung feeding insects have a specific a vertically transmitted microbiome warrants future studies.

## Supporting Information

Figure S1 **Sterile rearing.** DNA extractions and PCR amplification using the 10F and 1507R primers yielded an ~1500 bp product for fresh (unautoclaved) dung samples, homogenized dung beetle gut and a positive control (lanes UD, Gut,+). However, no such amplicon was produced from frozen or autoclaved dung samples, autoclaved sand and soil, and autoclaved water (lanes FD, AD, AS, AW). Lack of amplification was not due to an inhibitor present in the dung since adding either 0.5 µl or 1 µl of the positive control to the autoclaved products did produce an amplicon (Lane AD+ and AD++). Therefore, both freezing and autoclaving the dung and soil used for rearing the offspring degraded large fragments of bacterial DNA. Similarly, the rinse water of a surface sterilized beetle did not appear to inhibit amplicon production indicating that beetles can be effectively sterilized (Lane RW). The GeneRuler 1 kb ladder is used for reference (Lane Ld). Given these results, the microbiome profiles we examined are those that are inherited by the offspring from the female parent.
(TIF)

Figure S2 **A more detailed version of Figure 6 that includes the species or strain names of the taxa used to build the tree.**
(TIF)

Figure S3 **Dendrogram illustrating sequence identity between 16S rRNA sequences relative to the samples.** Abbreviations are the same as in Figure 4.
(TIF)

Figure S4 **Rarefaction curves for selected individuals where the frequency with which diversity is calculated every five sequences.**
(TIF)

Table S1 **Accession numbers and NCBI taxonomy of the bacterial genomes used for the phylotyping analysis.**
(DOCX)

Table S2 **Abbreviations and full names of the loci used for the phylotyping analysis.**
(DOCX)

Table S3 **Sequence identity matrix for 16S rRNA samples.** Cells labeled ID are where the sequence is compared to itself. Cells labeled NA were those where the sequence was not long enough for comparison.
(XLSX)
