# Supplementary figures and images for: Brood Ball-Mediated Transmission of Microbiome Members in the Dung Beetle, Onthophagus taurus (Coleoptera: Scarabaeidae)
Source: PLoS One. 2013 Nov 1;8(11):e79061. doi: 10.1371/journal.pone.0079061 (PMC3815100; doi:10.1371/journal.pone.0079061)

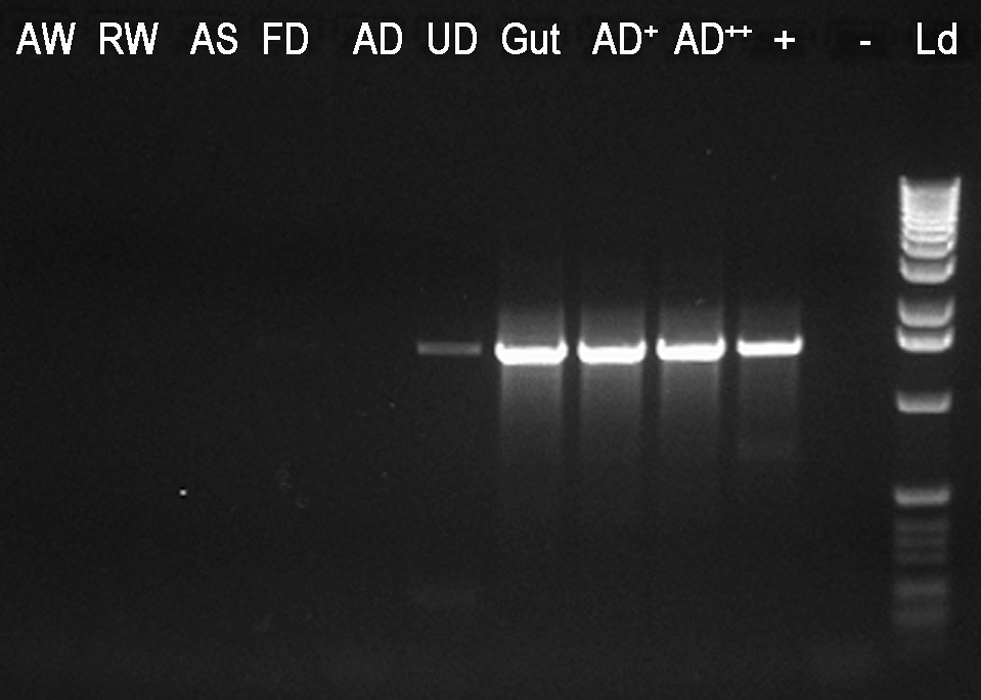

Supplement: Figure S1 — Sterile rearing. DNA extractions and PCR amplification using the 10F and 1507R primers yielded an ~1500 bp product for fresh (unautoclaved) dung samples, homogenized dung beetle gut and a positive control (lanes UD, Gut,+). However, no such amplicon was produced from frozen or autoclaved dung samples, autoclaved sand and soil, and autoclaved water (lanes FD, AD, AS, AW). Lack of amplification was not due to an inhibitor present in the dung since adding either 0.5 µl or 1 µl of the positive control to the autoclaved products did produce an amplicon (Lane AD+ and AD++). Therefore, both freezing and autoclaving the dung and soil used for rearing the offspring degraded large fragments of bacterial DNA. Similarly, the rinse water of a surface sterilized beetle did not appear to inhibit amplicon production indicating that beetles can be effectively sterilized (Lane RW). The GeneRuler 1 kb ladder is used for reference (Lane Ld). Given these results, the microbiome profiles we examined are those that are inherited by the offspring from the female parent. (TIF) [file pone.0079061.s001.tif]

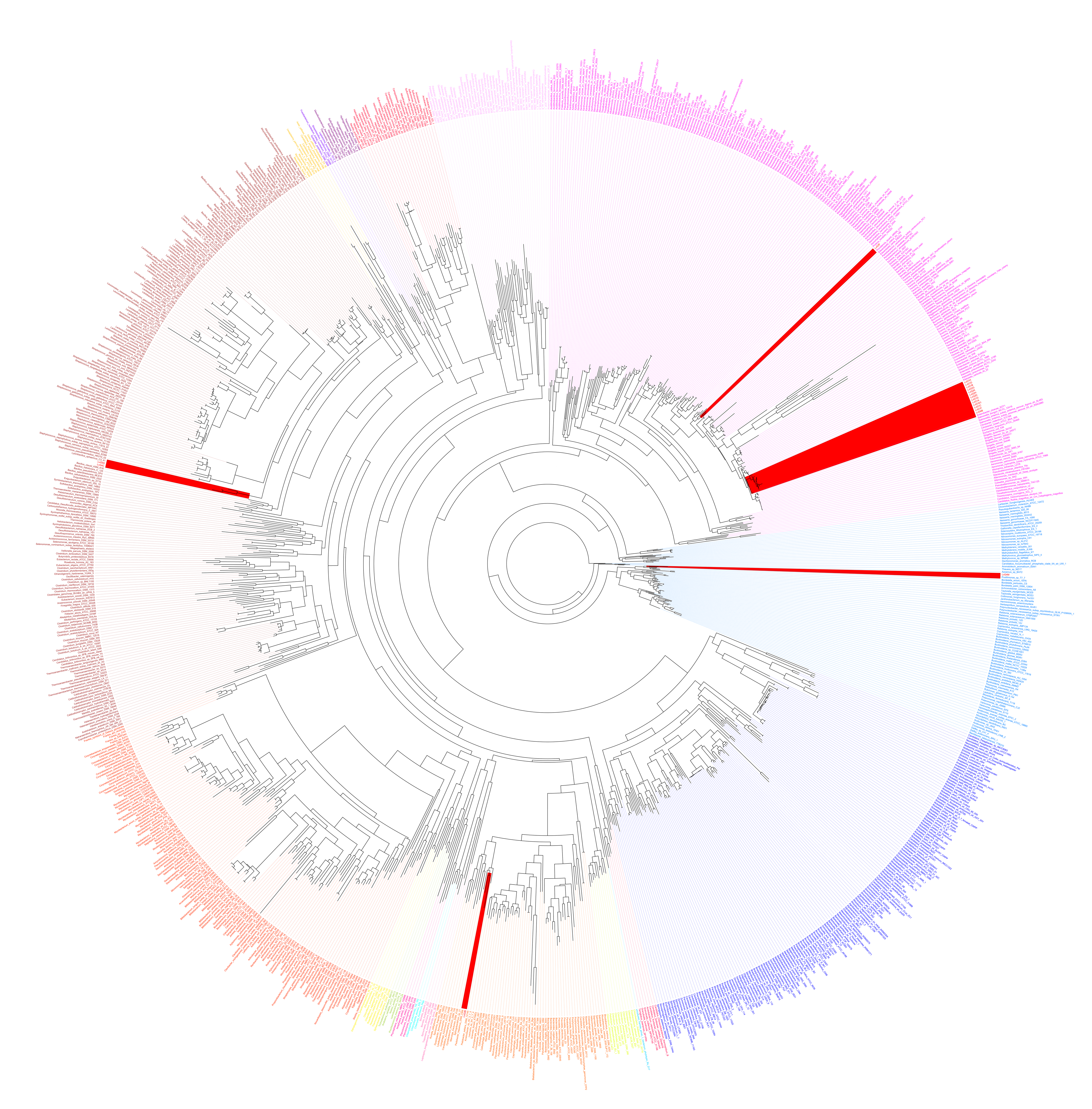

Supplement: Figure S2 — A more detailed version of Figure 6 that includes the species or strain names of the taxa used to build the tree. (TIF) [file pone.0079061.s002.tif]

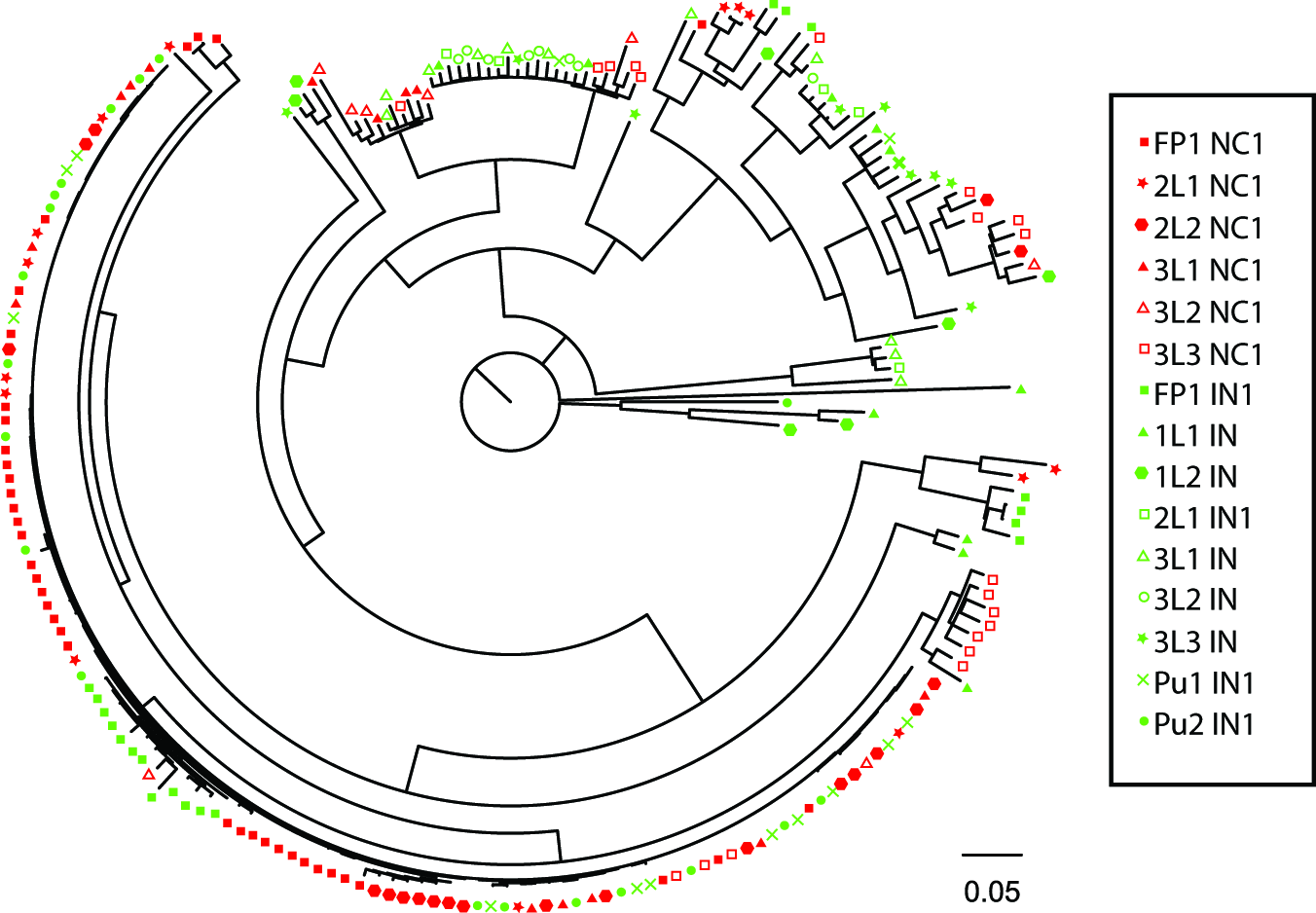

Supplement: Figure S3 — Dendrogram illustrating sequence identity between 16S rRNA sequences relative to the samples. Abbreviations are the same as in Figure 4. (TIF) [file pone.0079061.s003.tif]

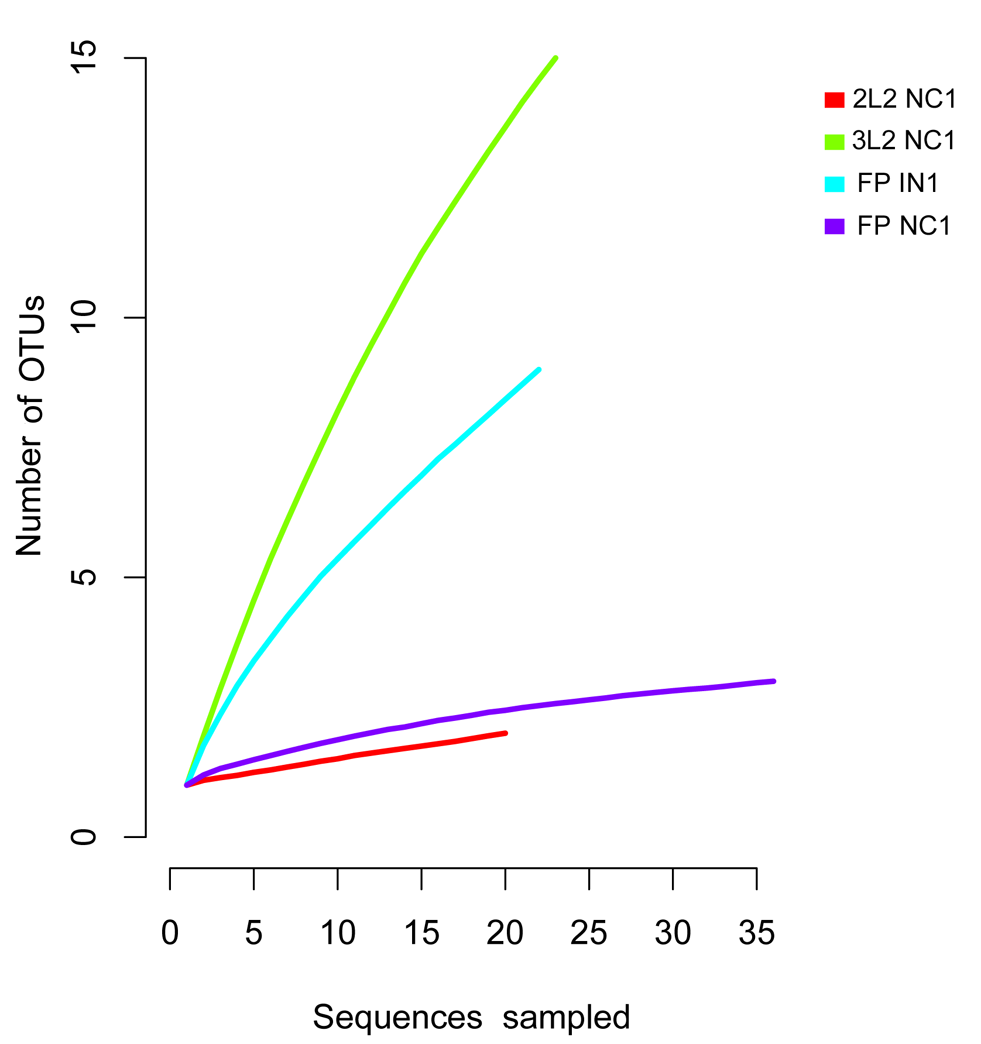

Supplement: Figure S4 — Rarefaction curves for selected individuals where the frequency with which diversity is calculated every five sequences. (TIF) [file pone.0079061.s004.tif]
