# Supplementary material for: Brood Ball-Mediated Transmission of Microbiome Members in the Dung Beetle, Onthophagus taurus (Coleoptera: Scarabaeidae)
Source: PLoS One. 2013 Nov 1;8(11):e79061. doi: 10.1371/journal.pone.0079061 (PMC3815100; doi:10.1371/journal.pone.0079061)
Supplement: Table S1 — Accession numbers and NCBI taxonomy of the bacterial genomes used for the phylotyping analysis. (DOCX) [file pone.0079061.s005.docx]

| **Genome** | **NCBI GenBank Accession Number** | **NCBI Taxonomy** |
| --- | --- | --- |
| Dechlorosoma_suillum_PS | NC_016616.1 | Dechlorosoma_suillum_PS:Azospira:Rhodocyclaceae:Rhodocyclales:Betaproteobacteria:Proteobacteria:Bacteria |
| Staphylococcus_lugdunensis_N920143 | NC_017353.1 | Staphylococcus lugdunensis N920143:Staphylococcus:Bacillales:Firmicutes:Bacteria |
| Thiobacillus_denitrificans  ATCC_25259 | NC_007404.1 | Thiobacillus denitrificans ATCC 25259:Thiobacillus:Hydrogenophilaceae:Hydrogenophilales:Betaproteobacteria:Proteobacteria:Bacteria |
| Mycoplasma_suis_KI3806 | NC_015153.1 | Mycoplasma suis KI3806:Mycoplasma:Mycoplasmataceae:Mollicutes:Tenericutes:Bacteria |
| Wolbachia_endosymbiont  of_Drosophila_melanogaster | NC_002978.6 | Wolbachia endosymbiont of Drosophila melanogaster:Wolbachia:Wolbachieae:Anaplasmataceae:Rickettsiales:Alphaproteobacteria:Proteobacteria:Bacteria |
| Variovorax_paradoxus_S110 | NC_012792.1 | Variovorax paradoxus S110:Variovorax:Comamonadaceae:Burkholderiales:Betaproteobacteria:Proteobacteria:Bacteria |
| Rickettsia_typhi_str_TH1527 | NC_017066.1 | Rickettsia typhi str. TH1527:typhus group:Rickettsia:Rickettsieae:Rickettsiaceae:Rickettsiales:Alphaproteobacteria:Proteobacteria:Bacteria |
| Desulfatibacillum_alkenivorans_AK_01 | NC_011768.1 | Desulfatibacillum alkenivorans AK-01:Desulfatibacillum:Desulfobacteraceae:Desulfobacterales:Deltaproteobacteria:Proteobacteria:Bacteria |
| Sulfurospirillum_deleyianum_DSM_6946 | NC_013512.1 | Sulfurospirillum deleyianum DSM 6946:Sulfurospirillum:Campylobacteraceae:Campylobacterales:Epsilonproteobacteria:Proteobacteria:Bacteria |
| Weeksella_virosa_DSM_16922 | NC_015144.1 | Weeksella virosa DSM 16922:Weeksella:Flavobacteriaceae:Flavobacteriales:Flavobacteriia:Bacteroidetes:Bacteria |
| Leptospira_borgpetersenii  serovar_Hardjo_bovis_JB197 | NC_008511.1 | Leptospira borgpetersenii serovar Hardjo-bovis JB197:Leptospira:Leptospiraceae:Spirochaetales:Spirochaetes:Bacteria |
| Lactobacillus_ruminis_ATCC_27782 | NC_015975.1 | Lactobacillus ruminis ATCC 27782:Lactobacillus:Lactobacillaceae:Lactobacillales:Firmicutes:Bacteria |
| Candidatus_Liberibacter  solanacearum_CLso_ZC1 | NC_014774.1 | Candidatus Liberibacter solanacearum CLso-ZC1:Candidatus Liberibacter:Rhizobiaceae:Rhizobiales:Alphaproteobacteria:Proteobacteria:Bacteria |
| Bifidobacterium_bifidum_PRL2010 | NC_014638.1 | Bifidobacterium bifidum PRL2010:Bifidobacterium:Bifidobacteriaceae:Bifidobacteriales:Actinobacteridae:Actinobacteria:Bacteria |
| Lactobacillus_johnsonii_FI9785 | NC_013504.1 | Lactobacillus johnsonii FI9785:Lactobacillus:Lactobacillaceae:Lactobacillales:Firmicutes:Bacteria |
| Sphaerobacter_thermophilus_DSM_20745 | NC_013524.1 | Sphaerobacter thermophilus DSM 20745:Sphaerobacter:Sphaerobacteraceae:Sphaerobacterineae:Sphaerobacterales:Sphaerobacteridae:Chloroflexi:Bacteria |
| Desulfomicrobium_baculatum_DSM_4028 | NC_013173.1 | Desulfomicrobium baculatum DSM 4028:Desulfomicrobium:Desulfomicrobiaceae:Desulfovibrionales:Deltaproteobacteria:Proteobacteria:Bacteria |
| Gluconacetobacter_diazotrophicus_PAl_5 | NC_011365.1 | Gluconacetobacter diazotrophicus PAl 5:Gluconacetobacter:Acetobacteraceae:Rhodospirillales:Alphaproteobacteria:Proteobacteria:Bacteria |
| Clostridium_lentocellum_DSM_5427 | NC_015275.1 | Clostridium lentocellum DSM 5427:Cellulosilyticum:Lachnospiraceae:Clostridiales:Clostridia:Firmicutes:Bacteria |
| Clostridium_thermocellum_ATCC_27405 | NC_009012.1 | Clostridium thermocellum ATCC 27405:Clostridium:Clostridiaceae:Clostridiales:Clostridia:Firmicutes:Bacteria |
| Streptococcus_pyogenes_Alab49 | NC_017596.1 | Streptococcus pyogenes Alab49:Streptococcus:Streptococcaceae:Lactobacillales:Firmicutes:Bacteria |
| Xanthomonas_axonopodis_pv_citri_str_306 | NC_003919.1 | Xanthomonas axonopodis pv. citri str. 306:Xanthomonas:Xanthomonadaceae:Xanthomonadales:Gammaproteobacteria:Proteobacteria:Bacteria |
| Methylotenera_versatilis_301 | NC_014207.1 | Methylotenera versatilis 301:Methylotenera:Methylophilaceae:Methylophilales:Betaproteobacteria:Proteobacteria:Bacteria |
| Pantoea_vagans_C9_1 | NC_014562.1 | Pantoea vagans C9-1:Pantoea:Enterobacteriaceae:Enterobacteriales:Gammaproteobacteria:Proteobacteria:Bacteria |
| Yersinia_pseudotuberculosis_IP_32953 | NC_006155.1 | Yersinia pseudotuberculosis IP 32953:Yersinia:Enterobacteriaceae:Enterobacteriales:Gammaproteobacteria:Proteobacteria:Bacteria |
| Shewanella_baltica_BA175 | NC_017571.1 | Shewanella baltica BA175:Shewanella:Shewanellaceae:Alteromonadales:Gammaproteobacteria:Proteobacteria:Bacteria |
| Pseudomonas_mendocina_NK_01 | NC_015410.1 | Pseudomonas mendocina NK-01:Pseudomonas:Pseudomonadaceae:Pseudomonadales:Gammaproteobacteria:Proteobacteria:Bacteria |
| Lactobacillus_plantarum  subsp_plantarum_ST_III | NC_014554.1 | Lactobacillus plantarum subsp. plantarum ST-III:Lactobacillus:Lactobacillaceae:Lactobacillales:Firmicutes:Bacteria |
| Methylobacterium_sp_4_46 | NC_010511.1 | Methylobacterium sp. 4-46:Methylobacterium:Methylobacteriaceae:Rhizobiales:Alphaproteobacteria:Proteobacteria:Bacteria |
| Polaromonas_sp_JS666 | NC_007948.1 | Polaromonas sp. JS666:Polaromonas:Comamonadaceae:Burkholderiales:Betaproteobacteria:Proteobacteria:Bacteria |
| Candidatus_Hamiltonella_defensa_5AT_Acyrthosiphon_pisum | NC_012751.1 | Candidatus Hamiltonella defensa 5AT (Acyrthosiphon pisum):Candidatus Hamiltonella:aphid secondary symbionts:Enterobacteriaceae:Enterobacteriales:Gammaproteobacteria:Proteobacteria:Bacteria |
| Kribbella_flavida_DSM_17836 | NC_013729.1 | Kribbella flavida DSM 17836:Kribbella:Nocardioidaceae:Propionibacterineae:Actinomycetales:Actinobacteridae:Actinobacteria:Bacteria |
| Paenibacillus_mucilaginosus_K02 | NC_017672.1 | Paenibacillus mucilaginosus K02:Paenibacillus:Paenibacillaceae:Bacillales:Firmicutes:Bacteria |
| Leuconostoc_mesenteroides_subsp_mesenteroides_J18 | NC_016805.1 | Leuconostoc mesenteroides subsp. mesenteroides J18:Leuconostoc:Lactobacillales:Firmicutes:Bacteria |
| Thermodesulfovibrio_yellowstonii_DSM_11347 | NC_011296.1 | Thermodesulfovibrio yellowstonii DSM 11347:Thermodesulfovibrio:Nitrospiraceae:Nitrospirales:Nitrospirae:Bacteria |
| Pseudomonas_putida_F1 | NC_009512.1 | Pseudomonas putida F1:Pseudomonas:Pseudomonadaceae:Pseudomonadales:Gammaproteobacteria:Proteobacteria:Bacteria |
| Mycobacterium_gilvum_PYR_GCK | NC_009338.1 | Mycobacterium gilvum PYR-GCK:Mycobacterium:Mycobacteriaceae:Corynebacterineae:Actinomycetales:Actinobacteridae:Actinobacteria:Bacteria |
| Caldicellulosiruptor_kristjanssonii_177R1B | NC_014721.1 | Caldicellulosiruptor kristjanssonii 177R1B:Caldicellulosiruptor:Incertae Sedis:Thermoanaerobacterales Family III:Thermoanaerobacterales:Clostridia:Firmicutes:Bacteria |
| Alicycliphilus_denitrificans_K601 | NC_015422.1 | Alicycliphilus denitrificans K601:Alicycliphilus:Comamonadaceae:Burkholderiales:Betaproteobacteria:Proteobacteria:Bacteria |
| Vibrio_parahaemolyticus_RIMD_2210633 | NC_004605.1 | Vibrio parahaemolyticus RIMD 2210633:Vibrio:Vibrionaceae:Vibrionales:Gammaproteobacteria:Proteobacteria:Bacteria |
| Nitratifractor_salsuginis_DSM_16511 | NC_014935.1 | Nitratifractor salsuginis DSM 16511:Nitratifractor:Epsilonproteobacteria:Proteobacteria:Bacteria |
| Glaciecola_nitratireducens_FR1064 | NC_016041.1 | Glaciecola nitratireducens FR1064:Glaciecola:Alteromonadaceae:Alteromonadales:Gammaproteobacteria:Proteobacteria:Bacteria |
| Arcobacter_butzleri_ED_1 | NC_017187.1 | Arcobacter butzleri ED-1:Arcobacter:Campylobacteraceae:Campylobacterales:Epsilonproteobacteria:Proteobacteria:Bacteria |
| Fibrobacter_succinogenes_subsp_succinogenes_S85 | NC_013410.1 | Fibrobacter succinogenes subsp. succinogenes S85:Fibrobacter:Fibrobacteraceae:Fibrobacterales:Fibrobacteres:Bacteria |
| Delftia_acidovorans_SPH_1 | NC_010002.1 | Delftia acidovorans SPH-1:Delftia:Comamonadaceae:Burkholderiales:Betaproteobacteria:Proteobacteria:Bacteria |
| Streptococcus_dysgalactiae_subsp_equisimilis_GGS_124 | NC_012891.1 | Streptococcus dysgalactiae subsp. equisimilis GGS_124:Streptococcus:Streptococcaceae:Lactobacillales:Firmicutes:Bacteria |
| Chlamydophila_abortus_S26_3 | NC_004552.2 | Chlamydophila abortus S26/3:Chlamydophila:Chlamydia/Chlamydophila group:Chlamydiaceae:Chlamydiales:Chlamydiae:Bacteria |
| Tannerella_forsythia_ATCC_43037 | NC_016610.1 | Tannerella forsythia ATCC 43037:Tannerella:Porphyromonadaceae:Bacteroidales:Bacteroidia:Bacteroidetes:Bacteria |
| Campylobacter_hominis  ATCC_BAA_381 | NC_009714.1 | Campylobacter hominis ATCC BAA-381:Campylobacter:Campylobacteraceae:Campylobacterales:Epsilonproteobacteria:Proteobacteria:Bacteria |
| Agrobacterium_sp_H13_3 | NC_015508.1 | Agrobacterium sp. H13-3:Agrobacterium:Rhizobium/Agrobacterium group:Rhizobiaceae:Rhizobiales:Alphaproteobacteria:Proteobacteria:Bacteria |
| Thermincola_potens_JR | NC_014152.1 | Thermincola potens JR:Thermincola:Peptococcaceae:Clostridiales:Clostridia:Firmicutes:Bacteria |
| Kyrpidia_tusciae_DSM_2912 | NC_014098.1 | Kyrpidia tusciae DSM 2912:Kyrpidia:Alicyclobacillaceae:Bacillales:Firmicutes:Bacteria |
| Clavibacter_michiganensis_subsp_michiganensis_NCPPB_382 | NC_009480.1 | Clavibacter michiganensis subsp. michiganensis NCPPB 382:Clavibacter:Microbacteriaceae:Micrococcineae:Actinomycetales:Actinobacteridae:Actinobacteria:Bacteria |
| Shewanella_amazonensis_SB2B | NC_008700.1 | Shewanella amazonensis SB2B:Shewanella:Shewanellaceae:Alteromonadales:Gammaproteobacteria:Proteobacteria:Bacteria |
| Mycobacterium_sp_JLS | NC_009077.1 | Mycobacterium sp. JLS:Mycobacterium:Mycobacteriaceae:Corynebacterineae:Actinomycetales:Actinobacteridae:Actinobacteria:Bacteria |
| Borrelia_burgdorferi_ZS7 | NC_011728.1 | Borrelia burgdorferi ZS7:Borrelia burgdorferi group:Borrelia:Spirochaetaceae:Spirochaetales:Spirochaetes:Bacteria |
| Ferrimonas_balearica_DSM_9799 | NC_014541.1 | Ferrimonas balearica DSM 9799:Ferrimonas:Ferrimonadaceae:Alteromonadales:Gammaproteobacteria:Proteobacteria:Bacteria |
| Thermoanaerobacterium_thermosaccharolyticum_DSM_571 | NC_014410.1 | Thermoanaerobacterium thermosaccharolyticum DSM 571:Thermoanaerobacterium:Incertae Sedis:Thermoanaerobacterales Family III:Thermoanaerobacterales:Clostridia:Firmicutes:Bacteria |
| Rickettsia_rhipicephali_str_3_7_female6_CWPP | NC_017042.1 | Rickettsia rhipicephali str. 3-7-female6-CWPP:spotted fever group:Rickettsia:Rickettsieae:Rickettsiaceae:Rickettsiales:Alphaproteobacteria:Proteobacteria:Bacteria |
| Acetobacter_pasteurianus_IFO_3283_01_42C | NC_017150.1 | Acetobacter pasteurianus IFO 3283-01-42C:Acetobacter:Acetobacteraceae:Rhodospirillales:Alphaproteobacteria:Proteobacteria:Bacteria |
| Geobacillus_thermoglucosidasius  C56_YS93 | NC_015660.1 | Geobacillus thermoglucosidasius C56-YS93:Geobacillus:Bacillaceae:Bacillales:Firmicutes:Bacteria |
| Candidatus_Liberibacter_asiaticus_str_psy62 | NC_012985.3 | Candidatus Liberibacter asiaticus str. psy62:Candidatus Liberibacter:Rhizobiaceae:Rhizobiales:Alphaproteobacteria:Proteobacteria:Bacteria |
| Lactobacillus_reuteri_JCM_1112 | NC_010609.1 | Lactobacillus reuteri JCM 1112:Lactobacillus:Lactobacillaceae:Lactobacillales:Firmicutes:Bacteria |
| Candidatus_Sulcia_muelleri_CARI | NC_014499.1 | Candidatus Sulcia muelleri CARI:Candidatus Sulcia:Flavobacteriales:Flavobacteriia:Bacteroidetes:Bacteria |
| Candidatus_Azobacteroides  pseudotrichonymphae_genomovar_CFP2 | NC_011565.1 | Candidatus Azobacteroides pseudotrichonymphae genomovar. CFP2:Candidatus Azobacteroides:Bacteroidales:Bacteroidia:Bacteroidetes:Bacteria |
| Mycobacterium_ulcerans_Agy99 | NC_008611.1 | Mycobacterium ulcerans Agy99:Mycobacterium:Mycobacteriaceae:Corynebacterineae:Actinomycetales:Actinobacteridae:Actinobacteria:Bacteria |
| Burkholderia_phymatum_STM815 | NC_010623.1 | Burkholderia phymatum STM815:Burkholderia:Burkholderiaceae:Burkholderiales:Betaproteobacteria:Proteobacteria:Bacteria |
| Renibacterium_salmoninarum_ATCC_33209 | NC_010168.1 | Renibacterium salmoninarum ATCC 33209:Renibacterium:Micrococcaceae:Micrococcineae:Actinomycetales:Actinobacteridae:Actinobacteria:Bacteria |
| Laribacter_hongkongensis_HLHK9 | NC_012559.1 | Laribacter hongkongensis HLHK9:Laribacter:Neisseriaceae:Neisseriales:Betaproteobacteria:Proteobacteria:Bacteria |
| Lactococcus_lactis_subsp_cremoris_MG1363 | NC_009004.1 | Lactococcus lactis subsp. cremoris MG1363:Lactococcus:Streptococcaceae:Lactobacillales:Firmicutes:Bacteria |
| Stackebrandtia_nassauensis_DSM_44728 | NC_013947.1 | Stackebrandtia nassauensis DSM 44728:Stackebrandtia:Glycomycetaceae:Glycomycineae:Actinomycetales:Actinobacteridae:Actinobacteria:Bacteria |
| Collimonas_fungivorans_Ter331 | NC_015856.1 | Collimonas fungivorans Ter331:Collimonas:Oxalobacteraceae:Burkholderiales:Betaproteobacteria:Proteobacteria:Bacteria |
| Klebsiella_oxytoca_KCTC_1686 | NC_016612.1 | Klebsiella oxytoca KCTC 1686:Klebsiella:Enterobacteriaceae:Enterobacteriales:Gammaproteobacteria:Proteobacteria:Bacteria |
| Staphylococcus_pseudintermedius_HKU10_03 | NC_014925.1 | Staphylococcus pseudintermedius HKU10-03:Staphylococcus:Bacillales:Firmicutes:Bacteria |
| Vibrio_cholerae_LMA3984_4 | NC_017270.1 | Vibrio cholerae LMA3984-4:Vibrio:Vibrionaceae:Vibrionales:Gammaproteobacteria:Proteobacteria:Bacteria |
| Myxococcus_xanthus_DK_1622 | NC_008095.1 | Myxococcus xanthus DK 1622:Myxococcus:Myxococcaceae:Cystobacterineae:Myxococcales:Deltaproteobacteria:Proteobacteria:Bacteria |
| Vibrio_fischeri_ES114 | NC_006841.2 | Vibrio fischeri ES114:Aliivibrio:Vibrionaceae:Vibrionales:Gammaproteobacteria:Proteobacteria:Bacteria |
| Rickettsia_montanensis_str_OSU_85_930 | NC_017043.1 | Rickettsia montanensis str. OSU 85-930:spotted fever group:Rickettsia:Rickettsieae:Rickettsiaceae:Rickettsiales:Alphaproteobacteria:Proteobacteria:Bacteria |
| Ruminococcus_albus_7 | NC_014833.1 | Ruminococcus albus 7:Ruminococcus:Ruminococcaceae:Clostridiales:Clostridia:Firmicutes:Bacteria |
| Erwinia_pyrifoliae_Ep1_96 | NC_012214.1 | Erwinia pyrifoliae Ep1/96:Erwinia:Enterobacteriaceae:Enterobacteriales:Gammaproteobacteria:Proteobacteria:Bacteria |
| Helicobacter_cetorum_MIT_99_5656 | NC_017735.1 | Helicobacter cetorum MIT 99-5656:Helicobacter:Helicobacteraceae:Campylobacterales:Epsilonproteobacteria:Proteobacteria:Bacteria |
| Lysinibacillus_sphaericus_C3_41 | NC_010382.1 | Lysinibacillus sphaericus C3-41:Lysinibacillus:Bacillaceae:Bacillales:Firmicutes:Bacteria |
| Odoribacter_splanchnicus_DSM_20712 | NC_015160.1 | Odoribacter splanchnicus DSM 20712:Odoribacter:Porphyromonadaceae:Bacteroidales:Bacteroidia:Bacteroidetes:Bacteria |
| Catenulispora_acidiphila_DSM_44928 | NC_013131.1 | Catenulispora acidiphila DSM 44928:Catenulispora:Catenulisporaceae:Catenulisporineae:Actinomycetales:Actinobacteridae:Actinobacteria:Bacteria |
| Rhodococcus_jostii_RHA1 | NC_008268.1 | Rhodococcus jostii RHA1:Rhodococcus:Nocardiaceae:Corynebacterineae:Actinomycetales:Actinobacteridae:Actinobacteria:Bacteria |
| Bartonella_henselae_str_Houston_1 | NC_005956.1 | Bartonella henselae str. Houston-1:Bartonella:Bartonellaceae:Rhizobiales:Alphaproteobacteria:Proteobacteria:Bacteria |
| Streptococcus_suis_05ZYH33 | NC_009442.1 | Streptococcus suis 05ZYH33:Streptococcus:Streptococcaceae:Lactobacillales:Firmicutes:Bacteria |
| Verminephrobacter_eiseniae_EF01_2 | NC_008786.1 | Verminephrobacter eiseniae EF01-2:Verminephrobacter:Comamonadaceae:Burkholderiales:Betaproteobacteria:Proteobacteria:Bacteria |
| Micavibrio_aeruginosavorus_ARL_13 | NC_016026.1 | Micavibrio aeruginosavorus ARL-13:Micavibrio:Alphaproteobacteria:Proteobacteria:Bacteria |
| Rickettsia_massiliae_MTU5 | NC_009900.1 | Rickettsia massiliae MTU5:spotted fever group:Rickettsia:Rickettsieae:Rickettsiaceae:Rickettsiales:Alphaproteobacteria:Proteobacteria:Bacteria |
| Serratia_symbiotica_str_Cinara_cedri | NC_016632.1 | Serratia symbiotica str. 'Cinara cedri':Serratia symbiotica:Serratia:Enterobacteriaceae:Enterobacteriales:Gammaproteobacteria:Proteobacteria:Bacteria |
| Candidatus_Zinderia_insecticola_CARI | NC_014497.1 | Candidatus Zinderia insecticola CARI:Candidatus Zinderia:Oxalobacteraceae:Burkholderiales:Betaproteobacteria:Proteobacteria:Bacteria |
| Owenweeksia_hongkongensis_DSM_17368 | NC_016599.1 | Owenweeksia hongkongensis DSM 17368:Owenweeksia:Cryomorphaceae:Flavobacteriales:Flavobacteriia:Bacteroidetes:Bacteria |
| Amycolatopsis_mediterranei_U32 | NC_014318.1 | Amycolatopsis mediterranei U32:Amycolatopsis:Pseudonocardiaceae:Pseudonocardineae:Actinomycetales:Actinobacteridae:Actinobacteria:Bacteria |
| cyanobacterium_UCYN_A | NC_013771.1 | cyanobacterium UCYN-A:Chroococcales:Cyanobacteria:Bacteria |
| Lactobacillus_casei_BD_II | NC_017474.1 | Lactobacillus casei BD-II:Lactobacillus:Lactobacillaceae:Lactobacillales:Firmicutes:Bacteria |
| Bifidobacterium_dentium_Bd1 | NC_013714.1 | Bifidobacterium dentium Bd1:Bifidobacterium:Bifidobacteriaceae:Bifidobacteriales:Actinobacteridae:Actinobacteria:Bacteria |
| Lactobacillus_salivarius_UCC118 | NC_007929.1 | Lactobacillus salivarius UCC118:Lactobacillus:Lactobacillaceae:Lactobacillales:Firmicutes:Bacteria |
| Nitrosomonas_europaea_ATCC_19718 | NC_004757.1 | Nitrosomonas europaea ATCC 19718:Nitrosomonas:Nitrosomonadaceae:Nitrosomonadales:Betaproteobacteria:Proteobacteria:Bacteria |
| Burkholderia_ambifaria_MC40_6 | NC_010557.1 | Burkholderia ambifaria MC40-6:Burkholderia cepacia complex:Burkholderia:Burkholderiaceae:Burkholderiales:Betaproteobacteria:Proteobacteria:Bacteria |
| Buchnera_aphidicola_str_5A_Acyrthosiphon_pisum | NC_011833.1 | Buchnera aphidicola str. 5A (Acyrthosiphon pisum):Buchnera:Enterobacteriaceae:Enterobacteriales:Gammaproteobacteria:Proteobacteria:Bacteria |
| Candidatus_Riesia_pediculicola_USDA | NC_014109.1 | Candidatus Riesia pediculicola USDA:Candidatus Riesia:Enterobacteriaceae:Enterobacteriales:Gammaproteobacteria:Proteobacteria:Bacteria |
| Mycoplasma_bovis_PG45 | NC_014760.1 | Mycoplasma bovis PG45:Mycoplasma:Mycoplasmataceae:Mollicutes:Tenericutes:Bacteria |
| Starkeya_novella_DSM_506 | NC_014217.1 | Starkeya novella DSM 506:Starkeya:Xanthobacteraceae:Rhizobiales:Alphaproteobacteria:Proteobacteria:Bacteria |
| Streptococcus_pneumoniae_70585 | NC_012468.1 | Streptococcus pneumoniae 70585:Streptococcus:Streptococcaceae:Lactobacillales:Firmicutes:Bacteria |
| Chlamydia_muridarum_Nigg | NC_002620.2 | Chlamydia muridarum Nigg:Chlamydia:Chlamydia/Chlamydophila group:Chlamydiaceae:Chlamydiales:Chlamydiae:Bacteria |
| Halothermothrix_orenii_H_168 | NC_011899.1 | Halothermothrix orenii H 168:Halothermothrix:Halanaerobiaceae:Halanaerobiales:Clostridia:Firmicutes:Bacteria |
| Haemophilus_somnus_2336 | NC_010519.1 | Haemophilus somnus 2336:Histophilus:Pasteurellaceae:Pasteurellales:Gammaproteobacteria:Proteobacteria:Bacteria |
| Marinobacter_adhaerens_HP15 | NC_017506.1 | Marinobacter adhaerens HP15:Marinobacter:Alteromonadaceae:Alteromonadales:Gammaproteobacteria:Proteobacteria:Bacteria |
| Xanthomonas_albilineans_GPE_PC73 | NC_013722.1 | Xanthomonas albilineans GPE PC73:Xanthomonas:Xanthomonadaceae:Xanthomonadales:Gammaproteobacteria:Proteobacteria:Bacteria |
| Methylobacterium_extorquens_AM1 | NC_012808.1 | Methylobacterium extorquens AM1:Methylobacterium:Methylobacteriaceae:Rhizobiales:Alphaproteobacteria:Proteobacteria:Bacteria |
| Bacillus_weihenstephanensis_KBAB4 | NC_010184.1 | Bacillus weihenstephanensis KBAB4:Bacillus cereus group:Bacillus:Bacillaceae:Bacillales:Firmicutes:Bacteria |
| Methylomonas_methanica_MC09 | NC_015572.1 | Methylomonas methanica MC09:Methylomonas:Methylococcaceae:Methylococcales:Gammaproteobacteria:Proteobacteria:Bacteria |
| Nocardioides_sp_JS614 | NC_008699.1 | Nocardioides sp. JS614:Nocardioides:Nocardioidaceae:Propionibacterineae:Actinomycetales:Actinobacteridae:Actinobacteria:Bacteria |
| Stenotrophomonas_maltophilia_JV3 | NC_015947.1 | Stenotrophomonas maltophilia JV3:Stenotrophomonas maltophilia group:Stenotrophomonas:Xanthomonadaceae:Xanthomonadales:Gammaproteobacteria:Proteobacteria:Bacteria |
| Blattabacterium_sp_Blattella_germanica_str_Bge | NC_013454.1 | Blattabacterium sp. (Blattella germanica) str. Bge:Blattabacterium:Blattabacteriaceae:Flavobacteriales:Flavobacteriia:Bacteroidetes:Bacteria |
| Methylovorus_sp_MP688 | NC_014733.1 | Methylovorus sp. MP688:Methylovorus:Methylophilaceae:Methylophilales:Betaproteobacteria:Proteobacteria:Bacteria |
| Corynebacterium_kroppenstedtii_DSM_44385 | NC_012704.1 | Corynebacterium kroppenstedtii DSM 44385:Corynebacterium:Corynebacteriaceae:Corynebacterineae:Actinomycetales:Actinobacteridae:Actinobacteria:Bacteria |
| Candidatus_Moranella_endobia_PCIT | NC_015735.1 | Candidatus Moranella endobia PCIT:Candidatus Moranella:Enterobacteriaceae:Enterobacteriales:Gammaproteobacteria:Proteobacteria:Bacteria |
| Eggerthella_lenta_DSM_2243 | NC_013204.1 | Eggerthella lenta DSM 2243:Eggerthella:Coriobacteriaceae:Coriobacterineae:Coriobacteriales:Coriobacteridae:Actinobacteria:Bacteria |
| Xylella_fastidiosa_M12 | NC_010513.1 | Xylella fastidiosa M12:Xylella:Xanthomonadaceae:Xanthomonadales:Gammaproteobacteria:Proteobacteria:Bacteria |
| Nitrosococcus_oceani_ATCC_19707 | NC_007484.1 | Nitrosococcus oceani ATCC 19707:Nitrosococcus:Chromatiaceae:Chromatiales:Gammaproteobacteria:Proteobacteria:Bacteria |
| Tolumonas_auensis_DSM_9187 | NC_012691.1 | Tolumonas auensis DSM 9187:Tolumonas:Aeromonadaceae:Aeromonadales:Gammaproteobacteria:Proteobacteria:Bacteria |
| Mycobacterium_gilvum_Spyr1 | NC_014814.1 | Mycobacterium gilvum Spyr1:Mycobacterium:Mycobacteriaceae:Corynebacterineae:Actinomycetales:Actinobacteridae:Actinobacteria:Bacteria |
| Rickettsia_prowazekii_Rp22 | NC_017560.1 | Rickettsia prowazekii Rp22:typhus group:Rickettsia:Rickettsieae:Rickettsiaceae:Rickettsiales:Alphaproteobacteria:Proteobacteria:Bacteria |
| Paenibacillus_sp_JDR_2 | NC_012914.1 | Paenibacillus sp. JDR-2:Paenibacillus:Paenibacillaceae:Bacillales:Firmicutes:Bacteria |
| Rhodobacter_sphaeroides_ATCC_17025 | NC_009428.1 | Rhodobacter sphaeroides ATCC 17025:Rhodobacter:Rhodobacteraceae:Rhodobacterales:Alphaproteobacteria:Proteobacteria:Bacteria |
| Enterobacter_sp_638 | NC_009436.1 | Enterobacter sp. 638:Enterobacter:Enterobacteriaceae:Enterobacteriales:Gammaproteobacteria:Proteobacteria:Bacteria |
| Pediococcus_claussenii_ATCC_BAA_344 | NC_016605.1 | Pediococcus claussenii ATCC BAA-344:Pediococcus:Lactobacillaceae:Lactobacillales:Firmicutes:Bacteria |
| Taylorella_asinigenitalis_MCE3 | NC_016043.1 | Taylorella asinigenitalis MCE3:Taylorella:Alcaligenaceae:Burkholderiales:Betaproteobacteria:Proteobacteria:Bacteria |
| Parabacteroides_distasonis_ATCC_8503 | NC_009615.1 | Parabacteroides distasonis ATCC 8503:Parabacteroides:Porphyromonadaceae:Bacteroidales:Bacteroidia:Bacteroidetes:Bacteria |
| Clostridium_kluyveri_NBRC_12016 | NC_011837.1 | Clostridium kluyveri NBRC 12016:Clostridium:Clostridiaceae:Clostridiales:Clostridia:Firmicutes:Bacteria |
| Novosphingobium_aromaticivorans_DSM_12444 | NC_007794.1 | Novosphingobium aromaticivorans DSM 12444:Novosphingobium:Sphingomonadaceae:Sphingomonadales:Alphaproteobacteria:Proteobacteria:Bacteria |
| Erwinia_amylovora_ATCC_49946 | NC_013971.1 | Erwinia amylovora ATCC 49946:Erwinia:Enterobacteriaceae:Enterobacteriales:Gammaproteobacteria:Proteobacteria:Bacteria |
| Bacillus_anthracis_str_Ames_Ancestor | NC_007530.2 | Bacillus anthracis str. 'Ames Ancestor':Bacillus cereus group:Bacillus:Bacillaceae:Bacillales:Firmicutes:Bacteria |
| Clostridium_sticklandii_DSM_519 | NC_014614.1 | Clostridium sticklandii DSM 519:Peptostreptococcaceae:Clostridiales:Clostridia:Firmicutes:Bacteria |
| Prosthecochloris_aestuarii_DSM_271 | NC_011059.1 | Prosthecochloris aestuarii DSM 271:Prosthecochloris:Chlorobiaceae:Chlorobiales:Chlorobia:Chlorobi:Bacteria |
| Candidatus_Tremblaya_princeps_PCIT | NC_015736.1 | Candidatus Tremblaya princeps PCIT:Candidatus Tremblaya:Betaproteobacteria:Proteobacteria:Bacteria |
| Acidovorax_sp_JS42 | NC_008782.1 | Acidovorax sp. JS42:Acidovorax:Comamonadaceae:Burkholderiales:Betaproteobacteria:Proteobacteria:Bacteria |
| Thermovirga_lienii_DSM_17291 | NC_016148.1 | Thermovirga lienii DSM 17291:Thermovirga:Synergistaceae:Synergistales:Synergistia:Synergistetes:Bacteria |
| Candidatus_Tremblaya_princeps_PCVAL | NC_017293.1 | Candidatus Tremblaya princeps PCVAL:Candidatus Tremblaya:Betaproteobacteria:Proteobacteria:Bacteria |
| Rahnella_sp_Y9602 | NC_015061.1 | Rahnella sp. Y9602:Rahnella:Enterobacteriaceae:Enterobacteriales:Gammaproteobacteria:Proteobacteria:Bacteria |
| Bacillus_clausii_KSM_K16 | NC_006582.1 | Bacillus clausii KSM-K16:Bacillus:Bacillaceae:Bacillales:Firmicutes:Bacteria |
| Arthrobacter_phenanthrenivorans_Sphe3 | NC_015145.1 | Arthrobacter phenanthrenivorans Sphe3:Arthrobacter:Micrococcaceae:Micrococcineae:Actinomycetales:Actinobacteridae:Actinobacteria:Bacteria |
| Brucella_suis_1330 | NC_017251.1 | Brucella suis 1330:Brucella:Brucellaceae:Rhizobiales:Alphaproteobacteria:Proteobacteria:Bacteria |
| Desulfotomaculum_ruminis_DSM_2154 | NC_015589.1 | Desulfotomaculum ruminis DSM 2154:Desulfotomaculum:Peptococcaceae:Clostridiales:Clostridia:Firmicutes:Bacteria |
| Porphyromonas_gingivalis_ATCC_33277 | NC_010729.1 | Porphyromonas gingivalis ATCC 33277:Porphyromonas:Porphyromonadaceae:Bacteroidales:Bacteroidia:Bacteroidetes:Bacteria |
| Desulfovibrio_aespoeensis_Aspo_2 | NC_014844.1 | Desulfovibrio aespoeensis Aspo-2:Desulfovibrio:Desulfovibrionaceae:Desulfovibrionales:Deltaproteobacteria:Proteobacteria:Bacteria |
| Rhodomicrobium_vannielii_ATCC_17100 | NC_014664.1 | Rhodomicrobium vannielii ATCC 17100:Rhodomicrobium:Hyphomicrobiaceae:Rhizobiales:Alphaproteobacteria:Proteobacteria:Bacteria |
| Halothiobacillus_neapolitanus_c2 | NC_013422.1 | Halothiobacillus neapolitanus c2:Halothiobacillus:Halothiobacillaceae:Chromatiales:Gammaproteobacteria:Proteobacteria:Bacteria |
| Desulfovibrio_salexigens_DSM_2638 | NC_012881.1 | Desulfovibrio salexigens DSM 2638:Desulfovibrio:Desulfovibrionaceae:Desulfovibrionales:Deltaproteobacteria:Proteobacteria:Bacteria |
| Candidatus_Rickettsia_amblyommii_str_GAT_30V | NC_017028.1 | Candidatus Rickettsia amblyommii str. GAT-30V:spotted fever group:Rickettsia:Rickettsieae:Rickettsiaceae:Rickettsiales:Alphaproteobacteria:Proteobacteria:Bacteria |
| Lactobacillus_delbrueckii_subsp_bulgaricus_2038 | NC_017469.1 | Lactobacillus delbrueckii subsp. bulgaricus 2038:Lactobacillus:Lactobacillaceae:Lactobacillales:Firmicutes:Bacteria |
| Shigella_boydii_CDC_3083_94 | NC_010658.1 | Shigella boydii CDC 3083-94:Shigella:Enterobacteriaceae:Enterobacteriales:Gammaproteobacteria:Proteobacteria:Bacteria |
| Rhizobium_etli_CFN_42 | NC_007761.1 | Rhizobium etli CFN 42:Rhizobium:Rhizobium/Agrobacterium group:Rhizobiaceae:Rhizobiales:Alphaproteobacteria:Proteobacteria:Bacteria |
| Clostridiales_genomosp_BVAB3_str_UPII9_5 | NC_013895.2 | Clostridiales genomosp. BVAB3 str. UPII9-5:Clostridiales:Clostridia:Firmicutes:Bacteria |
| Clostridium_perfringens_ATCC_13124 | NC_008261.1 | Clostridium perfringens ATCC 13124:Clostridium:Clostridiaceae:Clostridiales:Clostridia:Firmicutes:Bacteria |
| Candidatus_Accumulibacter_phosphatis_clade_IIA_str_UW_1 | NC_013194.1 | Candidatus Accumulibacter phosphatis clade IIA str. UW-1:Candidatus Accumulibacter:Betaproteobacteria:Proteobacteria:Bacteria |
| Acidiphilium_multivorum_AIU301 | NC_015186.1 | Acidiphilium multivorum AIU301:Acidiphilium:Acetobacteraceae:Rhodospirillales:Alphaproteobacteria:Proteobacteria:Bacteria |
| Haemophilus_influenzae_86_028NP | NC_007146.2 | Haemophilus influenzae 86-028NP:Haemophilus:Pasteurellaceae:Pasteurellales:Gammaproteobacteria:Proteobacteria:Bacteria |
| Halanaerobium_praevalens_DSM_2228 | NC_017455.1 | Halanaerobium praevalens DSM 2228:Halanaerobium:Halanaerobiaceae:Halanaerobiales:Clostridia:Firmicutes:Bacteria |
| Dickeya_zeae_Ech1591 | NC_012912.1 | Dickeya zeae Ech1591:Dickeya:Enterobacteriaceae:Enterobacteriales:Gammaproteobacteria:Proteobacteria:Bacteria |
| Shigella_dysenteriae_Sd197 | NC_007606.1 | Shigella dysenteriae Sd197:Shigella:Enterobacteriaceae:Enterobacteriales:Gammaproteobacteria:Proteobacteria:Bacteria |
| Parvibaculum_lavamentivorans_DS_1 | NC_009719.1 | Parvibaculum lavamentivorans DS-1:Parvibaculum:Phyllobacteriaceae:Rhizobiales:Alphaproteobacteria:Proteobacteria:Bacteria |
| Gardnerella_vaginalis_ATCC_14019 | NC_014644.1 | Gardnerella vaginalis ATCC 14019:Gardnerella:Bifidobacteriaceae:Bifidobacteriales:Actinobacteridae:Actinobacteria:Bacteria |
| Rhodospirillum_rubrum_F11 | NC_017584.1 | Rhodospirillum rubrum F11:Rhodospirillum:Rhodospirillaceae:Rhodospirillales:Alphaproteobacteria:Proteobacteria:Bacteria |
| Actinoplanes_missouriensis_431 | NC_017093.1 | Actinoplanes missouriensis 431:Actinoplanes:Micromonosporaceae:Micromonosporineae:Actinomycetales:Actinobacteridae:Actinobacteria:Bacteria |
| Xanthobacter_autotrophicus_Py2 | NC_009720.1 | Xanthobacter autotrophicus Py2:Xanthobacter:Xanthobacteraceae:Rhizobiales:Alphaproteobacteria:Proteobacteria:Bacteria |
| Mycobacterium_avium_104 | NC_008595.1 | Mycobacterium avium 104:Mycobacterium avium complex (MAC):Mycobacterium:Mycobacteriaceae:Corynebacterineae:Actinomycetales:Actinobacteridae:Actinobacteria:Bacteria |
| Rhodothermus_marinus_DSM_4252 | NC_013501.1 | Rhodothermus marinus DSM 4252:Rhodothermus:Rhodothermaceae:Incertae sedis:Bacteroidetes Order II:Bacteroidetes:Bacteria |
| Bordetella_avium_197N | NC_010645.1 | Bordetella avium 197N:Bordetella:Alcaligenaceae:Burkholderiales:Betaproteobacteria:Proteobacteria:Bacteria |
| Gramella_forsetii_KT0803 | NC_008571.1 | Gramella forsetii KT0803:Gramella:Flavobacteriaceae:Flavobacteriales:Flavobacteriia:Bacteroidetes:Bacteria |
| Paracoccus_denitrificans_PD1222 | NC_008687.1 | Paracoccus denitrificans PD1222:Paracoccus:Rhodobacteraceae:Rhodobacterales:Alphaproteobacteria:Proteobacteria:Bacteria |
| Streptococcus_agalactiae_A909 | NC_007432.1 | Streptococcus agalactiae A909:Streptococcus:Streptococcaceae:Lactobacillales:Firmicutes:Bacteria |
| Marinomonas_sp_MWYL1 | NC_009654.1 | Marinomonas sp. MWYL1:Marinomonas:Oceanospirillales:Gammaproteobacteria:Proteobacteria:Bacteria |
| Bacillus_thuringiensis_BMB171 | NC_014171.1 | Bacillus thuringiensis BMB171:Bacillus cereus group:Bacillus:Bacillaceae:Bacillales:Firmicutes:Bacteria |
| Thermoanaerobacter_italicus_Ab9 | NC_013921.1 | Thermoanaerobacter italicus Ab9:Thermoanaerobacter:Thermoanaerobacteraceae:Thermoanaerobacterales:Clostridia:Firmicutes:Bacteria |
| Geobacter_sulfurreducens_PCA | NC_002939.4 | Geobacter sulfurreducens PCA:Geobacter:Geobacteraceae:Desulfuromonadales:Deltaproteobacteria:Proteobacteria:Bacteria |
| Enterococcus_faecium_Aus0004 | NC_017022.1 | Enterococcus faecium Aus0004:Enterococcus:Enterococcaceae:Lactobacillales:Firmicutes:Bacteria |
| Francisella_novicida_U112 | NC_008601.1 | Francisella novicida U112:Francisella:Francisellaceae:Thiotrichales:Gammaproteobacteria:Proteobacteria:Bacteria |
| Helicobacter_bizzozeronii_CIII_1 | NC_015674.1 | Helicobacter bizzozeronii CIII-1:Helicobacter:Helicobacteraceae:Campylobacterales:Epsilonproteobacteria:Proteobacteria:Bacteria |
| Wigglesworthia_glossinidia_endosymbiont_of_Glossina_brevipalpis | NC_004344.2 | Wigglesworthia glossinidia endosymbiont of Glossina brevipalpis:Wigglesworthia:Enterobacteriaceae:Enterobacteriales:Gammaproteobacteria:Proteobacteria:Bacteria |
| Listeria_monocytogenes_08_5578 | NC_013766.1 | Listeria monocytogenes 08-5578:Listeria:Listeriaceae:Bacillales:Firmicutes:Bacteria |
| Acinetobacter_baumannii_AB0057 | NC_011586.1 | Acinetobacter baumannii AB0057:Acinetobacter calcoaceticus/baumannii complex:Acinetobacter:Moraxellaceae:Pseudomonadales:Gammaproteobacteria:Proteobacteria:Bacteria |
| Streptococcus_gordonii_str_Challis_substr_CH1 | NC_009785.1 | Streptococcus gordonii str. Challis substr. CH1:Streptococcus:Streptococcaceae:Lactobacillales:Firmicutes:Bacteria |
| Shigella_sonnei | NC_016822.1 | Shigella sonnei 53G:Shigella:Enterobacteriaceae:Enterobacteriales:Gammaproteobacteria:Proteobacteria:Bacteria |
| Desulfobacterium_autotrophicum_HRM2 | NC_012108.1 | Desulfobacterium autotrophicum HRM2:Desulfobacterium:Desulfobacteraceae:Desulfobacterales:Deltaproteobacteria:Proteobacteria:Bacteria |
| Salmonella_bongori_NCTC_12419 | NC_015761.1 | Salmonella bongori NCTC 12419:Salmonella:Enterobacteriaceae:Enterobacteriales:Gammaproteobacteria:Proteobacteria:Bacteria |
| Nitratiruptor_sp_SB155_2 | NC_009662.1 | Nitratiruptor sp. SB155-2:Nitratiruptor:Epsilonproteobacteria:Proteobacteria:Bacteria |
| Desulfovibrio_alaskensis_G20 | NC_007519.1 | Desulfovibrio alaskensis G20:Desulfovibrio:Desulfovibrionaceae:Desulfovibrionales:Deltaproteobacteria:Proteobacteria:Bacteria |
| Aster_yellows_witches_broom_phytoplasma_AYWB | NC_007716.1 | Aster yellows witches'-broom phytoplasma AYWB:Candidatus Phytoplasma asteris:Candidatus Phytoplasma:Acholeplasmataceae:Acholeplasmatales:Mollicutes:Tenericutes:Bacteria |
| Thermotoga_thermarum_DSM_5069 | NC_015707.1 | Thermotoga thermarum DSM 5069:Thermotoga:Thermotogaceae:Thermotogales:Thermotogae:Bacteria |
| Corynebacterium_pseudotuberculosis_267 | NC_017462.1 | Corynebacterium pseudotuberculosis 267:Corynebacterium:Corynebacteriaceae:Corynebacterineae:Actinomycetales:Actinobacteridae:Actinobacteria:Bacteria |
| Helicobacter_cetorum_MIT_00_7128 | NC_017737.1 | Helicobacter cetorum MIT 00-7128:Helicobacter:Helicobacteraceae:Campylobacterales:Epsilonproteobacteria:Proteobacteria:Bacteria |
| Thermomicrobium_roseum_DSM_5159 | NC_011959.1 | Thermomicrobium roseum DSM 5159:Thermomicrobium:Thermomicrobiaceae:Thermomicrobiales:Chloroflexi:Bacteria |
| Chlamydophila_pneumoniae_TW_183 | NC_005043.1 | Chlamydophila pneumoniae TW-183:Chlamydia:Chlamydia/Chlamydophila group:Chlamydiaceae:Chlamydiales:Chlamydiae:Bacteria |
| Mycobacterium_vanbaalenii_PYR_1 | NC_008726.1 | Mycobacterium vanbaalenii PYR-1:Mycobacterium:Mycobacteriaceae:Corynebacterineae:Actinomycetales:Actinobacteridae:Actinobacteria:Bacteria |
| Streptococcus_sanguinis_SK36 | NC_009009.1 | Streptococcus sanguinis SK36:Streptococcus:Streptococcaceae:Lactobacillales:Firmicutes:Bacteria |
| Streptococcus_macedonicus_ACA_DC_198 | NC_016749.1 | Streptococcus macedonicus ACA-DC 198:Streptococcus:Streptococcaceae:Lactobacillales:Firmicutes:Bacteria |
| Pediococcus_pentosaceus_ATCC_25745 | NC_008525.1 | Pediococcus pentosaceus ATCC 25745:Pediococcus:Lactobacillaceae:Lactobacillales:Firmicutes:Bacteria |
| Staphylococcus_epidermidis_RP62A | NC_002976.3 | Staphylococcus epidermidis RP62A:Staphylococcus:Bacillales:Firmicutes:Bacteria |
| Aminobacterium_colombiense_DSM_12261 | NC_014011.1 | Aminobacterium colombiense DSM 12261:Aminobacterium:Synergistaceae:Synergistales:Synergistia:Synergistetes:Bacteria |
| Nitrobacter_winogradskyi_Nb_255 | NC_007406.1 | Nitrobacter winogradskyi Nb-255:Nitrobacter:Bradyrhizobiaceae:Rhizobiales:Alphaproteobacteria:Proteobacteria:Bacteria |
| Ralstonia_eutropha_H16 | NC_008314.1 | Ralstonia eutropha H16:Cupriavidus:Burkholderiaceae:Burkholderiales:Betaproteobacteria:Proteobacteria:Bacteria |
| Melissococcus_plutonius | NC_016938.1 | Melissococcus plutonius DAT561:Melissococcus:Enterococcaceae:Lactobacillales:Firmicutes:Bacteria |
| Bacillus_cellulosilyticus_DSM_2522 | NC_014829.1 | Bacillus cellulosilyticus DSM 2522:Bacillus:Bacillaceae:Bacillales:Firmicutes:Bacteria |
| Thermomonospora_curvata_DSM_43183 | NC_013510.1 | Thermomonospora curvata DSM 43183:Thermomonospora:Thermomonosporaceae:Streptosporangineae:Actinomycetales:Actinobacteridae:Actinobacteria:Bacteria |
| Caldicellulosiruptor_bescii_DSM_6725 | NC_012034.1 | Caldicellulosiruptor bescii DSM 6725:Caldicellulosiruptor:Incertae Sedis:Thermoanaerobacterales Family III:Thermoanaerobacterales:Clostridia:Firmicutes:Bacteria |
| Sideroxydans_lithotrophicus_ES_1 | NC_013959.1 | Sideroxydans lithotrophicus ES-1:Sideroxydans:Gallionellaceae:Gallionellales:Betaproteobacteria:Proteobacteria:Bacteria |
| Bartonella_grahamii_as4aup | NC_012846.1 | Bartonella grahamii as4aup:Bartonella:Bartonellaceae:Rhizobiales:Alphaproteobacteria:Proteobacteria:Bacteria |
| Acidothermus_cellulolyticus_11B | NC_008578.1 | Acidothermus cellulolyticus 11B:Acidothermus:Acidothermaceae:Frankineae:Actinomycetales:Actinobacteridae:Actinobacteria:Bacteria |
| Maricaulis_maris_MCS10 | NC_008347.1 | Maricaulis maris MCS10:Maricaulis:Hyphomonadaceae:Rhodobacterales:Alphaproteobacteria:Proteobacteria:Bacteria |
| Mannheimia_succiniciproducens_MBEL55E | NC_006300.1 | Mannheimia succiniciproducens MBEL55E:Basfia:Pasteurellaceae:Pasteurellales:Gammaproteobacteria:Proteobacteria:Bacteria |
| Proteus_mirabilis_HI4320 | NC_010554.1 | Proteus mirabilis HI4320:Proteus:Enterobacteriaceae:Enterobacteriales:Gammaproteobacteria:Proteobacteria:Bacteria |
| gamma_proteobacterium_HdN1 | NC_014366.1 | gamma proteobacterium HdN1:Gammaproteobacteria:Proteobacteria:Bacteria |
| Novosphingobium_sp_PP1Y | NC_015580.1 | Novosphingobium sp. PP1Y:Novosphingobium:Sphingomonadaceae:Sphingomonadales:Alphaproteobacteria:Proteobacteria:Bacteria |
| Exiguobacterium_sp_AT1b | NC_012673.1 | Exiguobacterium sp. AT1b:Exiguobacterium:Incertae Sedis:Bacillales Family XII:Bacillales:Firmicutes:Bacteria |
| Roseobacter_denitrificans_OCh_114 | NC_008209.1 | Roseobacter denitrificans OCh 114:Roseobacter:Rhodobacteraceae:Rhodobacterales:Alphaproteobacteria:Proteobacteria:Bacteria |
| Syntrophothermus_lipocalidus_DSM_12680 | NC_014220.1 | Syntrophothermus lipocalidus DSM 12680:Syntrophothermus:Syntrophomonadaceae:Clostridiales:Clostridia:Firmicutes:Bacteria |
| Lactobacillus_casei_ATCC_334 | NC_008526.1 | Lactobacillus casei ATCC 334:Lactobacillus:Lactobacillaceae:Lactobacillales:Firmicutes:Bacteria |
| Burkholderia_cenocepacia_AU_1054 | NC_008062.1 | Burkholderia cenocepacia AU 1054:Burkholderia cepacia complex:Burkholderia:Burkholderiaceae:Burkholderiales:Betaproteobacteria:Proteobacteria:Bacteria |
| Lactobacillus_salivarius_CECT_5713 | NC_017481.1 | Lactobacillus salivarius CECT 5713:Lactobacillus:Lactobacillaceae:Lactobacillales:Firmicutes:Bacteria |
| Legionella_pneumophila_str_Corby | NC_009494.2 | Legionella pneumophila str. Corby:Legionella:Legionellaceae:Legionellales:Gammaproteobacteria:Proteobacteria:Bacteria |
| Actinobacillus_pleuropneumoniae_serovar_5b_str_L20 | NC_009053.1 | Actinobacillus pleuropneumoniae serovar 5b str. L20:Actinobacillus:Pasteurellaceae:Pasteurellales:Gammaproteobacteria:Proteobacteria:Bacteria |
| Bacillus_cytotoxicus_NVH_391_98 | NC_009674.1 | Bacillus cytotoxicus NVH 391-98:Bacillus cereus group:Bacillus:Bacillaceae:Bacillales:Firmicutes:Bacteria |
| Cellvibrio_japonicus_Ueda107 | NC_010995.1 | Cellvibrio japonicus Ueda107:Cellvibrio:Pseudomonadaceae:Pseudomonadales:Gammaproteobacteria:Proteobacteria:Bacteria |
| Leuconostoc_gasicomitatum_LMG_18811 | NC_014319.1 | Leuconostoc gasicomitatum LMG 18811:Leuconostoc:Lactobacillales:Firmicutes:Bacteria |
| Geobacillus_thermoleovorans_CCB_US3_UF5 | NC_016593.1 | Geobacillus thermoleovorans CCB_US3_UF5:Geobacillus:Bacillaceae:Bacillales:Firmicutes:Bacteria |
| Lactobacillus_acidophilus_NCFM | NC_006814.3 | Lactobacillus acidophilus NCFM:Lactobacillus:Lactobacillaceae:Lactobacillales:Firmicutes:Bacteria |
| Verrucosispora_maris_AB_18_032 | NC_015434.1 | Verrucosispora maris AB-18-032:Verrucosispora:Micromonosporaceae:Micromonosporineae:Actinomycetales:Actinobacteridae:Actinobacteria:Bacteria |
| Rhodoferax_ferrireducens_T118 | NC_007908.1 | Rhodoferax ferrireducens T118:Albidiferax:Comamonadaceae:Burkholderiales:Betaproteobacteria:Proteobacteria:Bacteria |
| Helicobacter_pylori_2017 | NC_017374.1 | Helicobacter pylori 2017:Helicobacter:Helicobacteraceae:Campylobacterales:Epsilonproteobacteria:Proteobacteria:Bacteria |
| Acetobacter_pasteurianus_IFO_3283_01 | NC_013209.1 | Acetobacter pasteurianus IFO 3283-01:Acetobacter:Acetobacteraceae:Rhodospirillales:Alphaproteobacteria:Proteobacteria:Bacteria |
| Paenibacillus_polymyxa_M1 | NC_017542.1 | Paenibacillus polymyxa M1:Paenibacillus:Paenibacillaceae:Bacillales:Firmicutes:Bacteria |
| Sphingobium_chlorophenolicum_L_1 | NC_015594.1 | Sphingobium chlorophenolicum L-1:Sphingobium:Sphingomonadaceae:Sphingomonadales:Alphaproteobacteria:Proteobacteria:Bacteria |
| Xenorhabdus_bovienii_SS_2004 | NC_013892.1 | Xenorhabdus bovienii SS-2004:Xenorhabdus:Enterobacteriaceae:Enterobacteriales:Gammaproteobacteria:Proteobacteria:Bacteria |
| Streptococcus_suis_98HAH33 | NC_009443.1 | Streptococcus suis 98HAH33:Streptococcus:Streptococcaceae:Lactobacillales:Firmicutes:Bacteria |
| Listeria_ivanovii | NC_016011.1 | Listeria ivanovii subsp. ivanovii PAM 55:Listeria:Listeriaceae:Bacillales:Firmicutes:Bacteria |
| Sulfuricurvum_kujiense_DSM_16994 | NC_014762.1 | Sulfuricurvum kujiense DSM 16994:Sulfuricurvum:Helicobacteraceae:Campylobacterales:Epsilonproteobacteria:Proteobacteria:Bacteria |
| Saprospira_grandis_str_Lewin | NC_016940.1 | Saprospira grandis str. Lewin:Saprospira:Saprospiraceae:Sphingobacteriales:Sphingobacteriia:Bacteroidetes:Bacteria |
| Vibrio_splendidus_LGP32 | NC_011753.2 | Vibrio splendidus LGP32:Vibrio:Vibrionaceae:Vibrionales:Gammaproteobacteria:Proteobacteria:Bacteria |
| Haemophilus_parasuis_SH0165 | NC_011852.1 | Haemophilus parasuis SH0165:Haemophilus:Pasteurellaceae:Pasteurellales:Gammaproteobacteria:Proteobacteria:Bacteria |
| Cellulophaga_lytica_DSM_7489 | NC_015167.1 | Cellulophaga lytica DSM 7489:Cellulophaga:Flavobacteriaceae:Flavobacteriales:Flavobacteriia:Bacteroidetes:Bacteria |
| Acidobacterium_capsulatum_ATCC_51196 | NC_012483.1 | Acidobacterium capsulatum ATCC 51196:Acidobacterium:Acidobacteriaceae:Acidobacteriales:Acidobacteria:Bacteria |
| Arcanobacterium_haemolyticum_DSM_20595 | NC_014218.1 | Arcanobacterium haemolyticum DSM 20595:Arcanobacterium:Actinomycetaceae:Actinomycineae:Actinomycetales:Actinobacteridae:Actinobacteria:Bacteria |
| Saccharomonospora_viridis_DSM_43017 | NC_013159.1 | Saccharomonospora viridis DSM 43017:Saccharomonospora:Pseudonocardiaceae:Pseudonocardineae:Actinomycetales:Actinobacteridae:Actinobacteria:Bacteria |
| Yersinia_pseudotuberculosis_IP_31758 | NC_009708.1 | Yersinia pseudotuberculosis IP 31758:Yersinia:Enterobacteriaceae:Enterobacteriales:Gammaproteobacteria:Proteobacteria:Bacteria |
| Clostridium_novyi_NT | NC_008593.1 | Clostridium novyi NT:Clostridium:Clostridiaceae:Clostridiales:Clostridia:Firmicutes:Bacteria |
| Bacillus_sp_JS | NC_017743.1 | Bacillus sp. JS:Bacillus:Bacillaceae:Bacillales:Firmicutes:Bacteria |
| Alicycliphilus_denitrificans_BC | NC_014910.1 | Alicycliphilus denitrificans BC:Alicycliphilus:Comamonadaceae:Burkholderiales:Betaproteobacteria:Proteobacteria:Bacteria |
| Orientia_tsutsugamushi_str_Ikeda | NC_010793.1 | Orientia tsutsugamushi str. Ikeda:Orientia:Rickettsieae:Rickettsiaceae:Rickettsiales:Alphaproteobacteria:Proteobacteria:Bacteria |
| Burkholderia_vietnamiensis_G4 | NC_009256.1 | Burkholderia vietnamiensis G4:Burkholderia cepacia complex:Burkholderia:Burkholderiaceae:Burkholderiales:Betaproteobacteria:Proteobacteria:Bacteria |
| Salmonella_enterica_subsp_arizonae_serovar_62_str_RSK2980 | NC_010067.1 | Salmonella enterica subsp. arizonae serovar 62:z4,z23:-- str. RSK2980:arizonae serovar 62:z4,z23:--:Salmonella enterica subsp:Salmonella:Enterobacteriaceae:Enterobacteriales:Gammaproteobacteria:Proteobacteria:Bacteria |
| Frankia_symbiont_of_Datisca_glomerata | NC_015656.1 | Frankia symbiont of Datisca glomerata:Frankia:Frankiaceae:Frankineae:Actinomycetales:Actinobacteridae:Actinobacteria:Bacteria |
| Rhodospirillum_rubrum_ATCC_11170 | NC_007643.1 | Rhodospirillum rubrum ATCC 11170:Rhodospirillum:Rhodospirillaceae:Rhodospirillales:Alphaproteobacteria:Proteobacteria:Bacteria |
| Blastococcus_saxobsidens | NC_016943.1 | Blastococcus saxobsidens DD2:Blastococcus:Geodermatophilaceae:Frankineae:Actinomycetales:Actinobacteridae:Actinobacteria:Bacteria |
| Streptococcus_pyogenes_M1_GAS | NC_002737.1 | Streptococcus pyogenes M1 GAS:Streptococcus:Streptococcaceae:Lactobacillales:Firmicutes:Bacteria |
| Bacteroides_salanitronis_DSM_18170 | NC_015164.1 | Bacteroides salanitronis DSM 18170:Bacteroides:Bacteroidaceae:Bacteroidales:Bacteroidia:Bacteroidetes:Bacteria |
| Acinetobacter_oleivorans_DR1 | NC_014259.1 | Acinetobacter oleivorans DR1:Acinetobacter:Moraxellaceae:Pseudomonadales:Gammaproteobacteria:Proteobacteria:Bacteria |
| Bacillus_anthracis_str_Ames | NC_003997.3 | Bacillus anthracis str. Ames:Bacillus cereus group:Bacillus:Bacillaceae:Bacillales:Firmicutes:Bacteria |
| Candidatus_Chloracidobacterium_thermophilum_B | NC_016025.1 | Candidatus Chloracidobacterium thermophilum B:Candidatus Chloracidobacterium:Acidobacteria:Bacteria |
| Desulfarculus_baarsii_DSM_2075 | NC_014365.1 | Desulfarculus baarsii DSM 2075:Desulfarculus:Desulfarculaceae:Desulfarculales:Deltaproteobacteria:Proteobacteria:Bacteria |
| Sulfurovum_sp_NBC37_1 | NC_009663.1 | Sulfurovum sp. NBC37-1:Sulfurovum:Epsilonproteobacteria:Proteobacteria:Bacteria |
| Lactobacillus_fermentum_CECT_5716 | NC_017465.1 | Lactobacillus fermentum CECT 5716:Lactobacillus:Lactobacillaceae:Lactobacillales:Firmicutes:Bacteria |
| Bifidobacterium_longum_subsp_longum_KACC_91563 | NC_017221.1 | Bifidobacterium longum subsp. longum KACC 91563:Bifidobacterium:Bifidobacteriaceae:Bifidobacteriales:Actinobacteridae:Actinobacteria:Bacteria |
| Clostridium_ljungdahlii_DSM_13528 | NC_014328.1 | Clostridium ljungdahlii DSM 13528:Clostridium:Clostridiaceae:Clostridiales:Clostridia:Firmicutes:Bacteria |
| Syntrophus_aciditrophicus_SB | NC_007759.1 | Syntrophus aciditrophicus SB:Syntrophus:Syntrophaceae:Syntrophobacterales:Deltaproteobacteria:Proteobacteria:Bacteria |
| Cryptobacterium_curtum_DSM_15641 | NC_013170.1 | Cryptobacterium curtum DSM 15641:Cryptobacterium:Coriobacteriaceae:Coriobacterineae:Coriobacteriales:Coriobacteridae:Actinobacteria:Bacteria |
| Oceanobacillus_iheyensis_HTE831 | NC_004193.1 | Oceanobacillus iheyensis HTE831:Oceanobacillus:Bacillaceae:Bacillales:Firmicutes:Bacteria |
| Bacillus_megaterium_DSM_319 | NC_014103.1 | Bacillus megaterium DSM 319:Bacillus:Bacillaceae:Bacillales:Firmicutes:Bacteria |
| Cupriavidus_metallidurans_CH34 | NC_007973.1 | Cupriavidus metallidurans CH34:Cupriavidus:Burkholderiaceae:Burkholderiales:Betaproteobacteria:Proteobacteria:Bacteria |
| Buchnera_aphidicola_Cinara_tujafilina | NC_015662.1 | Buchnera aphidicola (Cinara tujafilina):Buchnera:Enterobacteriaceae:Enterobacteriales:Gammaproteobacteria:Proteobacteria:Bacteria |
| Filifactor_alocis_ATCC_35896 | NC_016630.1 | Filifactor alocis ATCC 35896:Filifactor:Peptostreptococcaceae:Clostridiales:Clostridia:Firmicutes:Bacteria |
| Parvularcula_bermudensis_HTCC2503 | NC_014414.1 | Parvularcula bermudensis HTCC2503:Parvularcula:Parvularculaceae:Parvularculales:Alphaproteobacteria:Proteobacteria:Bacteria |
| Klebsiella_pneumoniae_342 | NC_011283.1 | Klebsiella pneumoniae 342:Klebsiella:Enterobacteriaceae:Enterobacteriales:Gammaproteobacteria:Proteobacteria:Bacteria |
| Saccharophagus_degradans_2_40 | NC_007912.1 | Saccharophagus degradans 2-40:Saccharophagus:Alteromonadaceae:Alteromonadales:Gammaproteobacteria:Proteobacteria:Bacteria |
| Sulfobacillus_acidophilus_DSM_10332 | NC_016884.1 | Sulfobacillus acidophilus DSM 10332:Sulfobacillus:Incertae Sedis:Clostridiales Family XVII:Clostridiales:Clostridia:Firmicutes:Bacteria |
| Rickettsia_conorii_str_Malish_7 | NC_003103.1 | Rickettsia conorii str. Malish 7:spotted fever group:Rickettsia:Rickettsieae:Rickettsiaceae:Rickettsiales:Alphaproteobacteria:Proteobacteria:Bacteria |
| Mycobacterium_avium_subsp_paratuberculosis_K_10 | NC_002944.2 | Mycobacterium avium subsp. paratuberculosis K-10:Mycobacterium avium complex (MAC):Mycobacterium:Mycobacteriaceae:Corynebacterineae:Actinomycetales:Actinobacteridae:Actinobacteria:Bacteria |
| Conexibacter_woesei_DSM_14684 | NC_013739.1 | Conexibacter woesei DSM 14684:Conexibacter:Conexibacteraceae:Solirubrobacterales:Rubrobacteridae:Actinobacteria:Bacteria |
| Pelagibacterium_halotolerans_B2 | NC_016078.1 | Pelagibacterium halotolerans B2:Pelagibacterium:Hyphomicrobiaceae:Rhizobiales:Alphaproteobacteria:Proteobacteria:Bacteria |
| Citrobacter_koseri_ATCC_BAA_895 | NC_009792.1 | Citrobacter koseri ATCC BAA-895:Citrobacter:Enterobacteriaceae:Enterobacteriales:Gammaproteobacteria:Proteobacteria:Bacteria |
| Gordonia_polyisoprenivorans_VH2 | NC_016906.1 | Gordonia polyisoprenivorans VH2:Gordonia:Gordoniaceae:Corynebacterineae:Actinomycetales:Actinobacteridae:Actinobacteria:Bacteria |
| Geobacter_lovleyi_SZ | NC_010814.1 | Geobacter lovleyi SZ:Geobacter:Geobacteraceae:Desulfuromonadales:Deltaproteobacteria:Proteobacteria:Bacteria |
| Candidatus_Desulforudis_audaxviator_MP104C | NC_010424.1 | Candidatus Desulforudis audaxviator MP104C:Candidatus Desulforudis:Peptococcaceae:Clostridiales:Clostridia:Firmicutes:Bacteria |
| Methylovorus_glucosetrophus_SIP3_4 | NC_012969.1 | Methylovorus glucosetrophus SIP3-4:Methylovorus:Methylophilaceae:Methylophilales:Betaproteobacteria:Proteobacteria:Bacteria |
| Rothia_mucilaginosa_DY_18 | NC_013715.1 | Rothia mucilaginosa DY-18:Rothia:Micrococcaceae:Micrococcineae:Actinomycetales:Actinobacteridae:Actinobacteria:Bacteria |
| Carboxydothermus_hydrogenoformans_Z_2901 | NC_007503.1 | Carboxydothermus hydrogenoformans Z-2901:Carboxydothermus:Thermoanaerobacteraceae:Thermoanaerobacterales:Clostridia:Firmicutes:Bacteria |
| Caldicellulosiruptor_kronotskyensis_2002 | NC_014720.1 | Caldicellulosiruptor kronotskyensis 2002:Caldicellulosiruptor:Incertae Sedis:Thermoanaerobacterales Family III:Thermoanaerobacterales:Clostridia:Firmicutes:Bacteria |
| Mycobacterium_sp_JDM601 | NC_015576.1 | Mycobacterium sp. JDM601:Mycobacterium:Mycobacteriaceae:Corynebacterineae:Actinomycetales:Actinobacteridae:Actinobacteria:Bacteria |
| Granulibacter_bethesdensis_CGDNIH1 | NC_008343.1 | Granulibacter bethesdensis CGDNIH1:Granulibacter:Acetobacteraceae:Rhodospirillales:Alphaproteobacteria:Proteobacteria:Bacteria |
| Dictyoglomus_thermophilum_H_6_12 | NC_011297.1 | Dictyoglomus thermophilum H-6-12:Dictyoglomus:Dictyoglomaceae:Dictyoglomales:Dictyoglomi:Bacteria |
| Micromonospora_sp_L5 | NC_014815.1 | Micromonospora sp. L5:Micromonospora:Micromonosporaceae:Micromonosporineae:Actinomycetales:Actinobacteridae:Actinobacteria:Bacteria |
| Brucella_suis_ATCC_23445 | NC_010169.1 | Brucella suis ATCC 23445:Brucella:Brucellaceae:Rhizobiales:Alphaproteobacteria:Proteobacteria:Bacteria |
| Thermodesulfobium_narugense_DSM_14796 | NC_015499.1 | Thermodesulfobium narugense DSM 14796:Thermodesulfobium:Thermodesulfobiaceae:Thermoanaerobacterales:Clostridia:Firmicutes:Bacteria |
| Dickeya_dadantii_Ech586 | NC_013592.1 | Dickeya dadantii Ech586:Dickeya:Enterobacteriaceae:Enterobacteriales:Gammaproteobacteria:Proteobacteria:Bacteria |
| Elusimicrobium_minutum_Pei191 | NC_010644.1 | Elusimicrobium minutum Pei191:Elusimicrobium:Elusimicrobiaceae:Elusimicrobiales:Elusimicrobia:Elusimicrobia:Bacteria |
| Halomonas_elongata_DSM_2581 | NC_014532.1 | Halomonas elongata DSM 2581:Halomonas:Halomonadaceae:Oceanospirillales:Gammaproteobacteria:Proteobacteria:Bacteria |
| Chelativorans_sp_BNC1 | NC_008254.1 | Chelativorans sp. BNC1:Chelativorans:Phyllobacteriaceae:Rhizobiales:Alphaproteobacteria:Proteobacteria:Bacteria |
| Desulfurivibrio_alkaliphilus_AHT2 | NC_014216.1 | Desulfurivibrio alkaliphilus AHT2:Desulfurivibrio:Desulfobulbaceae:Desulfobacterales:Deltaproteobacteria:Proteobacteria:Bacteria |
| Sorangium_cellulosum_So_ce_56 | NC_010162.1 | Sorangium cellulosum 'So ce 56':Sorangium:Polyangiaceae:Sorangiineae:Myxococcales:Deltaproteobacteria:Proteobacteria:Bacteria |
| Bartonella_bacilliformis_KC583 | NC_008783.1 | Bartonella bacilliformis KC583:Bartonella:Bartonellaceae:Rhizobiales:Alphaproteobacteria:Proteobacteria:Bacteria |
| Mahella_australiensis_50_1_BON | NC_015520.1 | Mahella australiensis 50-1 BON:Mahella:Incertae Sedis:Thermoanaerobacterales Family IV:Thermoanaerobacterales:Clostridia:Firmicutes:Bacteria |
| Mesorhizobium_ciceri_biovar_biserrulae_WSM1271 | NC_014923.1 | Mesorhizobium ciceri biovar biserrulae WSM1271:Mesorhizobium:Phyllobacteriaceae:Rhizobiales:Alphaproteobacteria:Proteobacteria:Bacteria |
| Haliscomenobacter_hydrossis_DSM_1100 | NC_015510.1 | Haliscomenobacter hydrossis DSM 1100:Haliscomenobacter:Saprospiraceae:Sphingobacteriales:Sphingobacteriia:Bacteroidetes:Bacteria |
| Nocardia_cyriacigeorgica | NC_016887.1 | Nocardia cyriacigeorgica GUH-2:Nocardia:Nocardiaceae:Corynebacterineae:Actinomycetales:Actinobacteridae:Actinobacteria:Bacteria |
| Thermobifida_fusca_YX | NC_007333.1 | Thermobifida fusca YX:Thermobifida:Nocardiopsaceae:Streptosporangineae:Actinomycetales:Actinobacteridae:Actinobacteria:Bacteria |
| Bartonella_tribocorum_CIP_105476 | NC_010161.1 | Bartonella tribocorum CIP 105476:Bartonella:Bartonellaceae:Rhizobiales:Alphaproteobacteria:Proteobacteria:Bacteria |
| Ralstonia_solanacearum_CFBP2957 | NC_014307.1 | Ralstonia solanacearum CFBP2957:Ralstonia:Burkholderiaceae:Burkholderiales:Betaproteobacteria:Proteobacteria:Bacteria |
| Sinorhizobium_fredii_NGR234 | NC_012587.1 | Sinorhizobium fredii NGR234:Sinorhizobium:Sinorhizobium/Ensifer group:Rhizobiaceae:Rhizobiales:Alphaproteobacteria:Proteobacteria:Bacteria |
| Rhodococcus_equi_103S | NC_014659.1 | Rhodococcus equi 103S:Rhodococcus:Nocardiaceae:Corynebacterineae:Actinomycetales:Actinobacteridae:Actinobacteria:Bacteria |
| Pseudomonas_stutzeri_DSM_4166 | NC_017532.1 | Pseudomonas stutzeri DSM 4166:Pseudomonas:Pseudomonadaceae:Pseudomonadales:Gammaproteobacteria:Proteobacteria:Bacteria |
| Veillonella_parvula_DSM_2008 | NC_013520.1 | Veillonella parvula DSM 2008:Veillonella:Veillonellaceae:Selenomonadales:Negativicutes:Firmicutes:Bacteria |
| Caldicellulosiruptor_saccharolyticus_DSM_8903 | NC_009437.1 | Caldicellulosiruptor saccharolyticus DSM 8903:Caldicellulosiruptor:Incertae Sedis:Thermoanaerobacterales Family III:Thermoanaerobacterales:Clostridia:Firmicutes:Bacteria |
| Tsukamurella_paurometabola_DSM_20162 | NC_014158.1 | Tsukamurella paurometabola DSM 20162:Tsukamurella:Tsukamurellaceae:Corynebacterineae:Actinomycetales:Actinobacteridae:Actinobacteria:Bacteria |
| Fibrobacter_succinogenes_subsp_succinogenes_S85 | NC_017448.1 | Fibrobacter succinogenes subsp. succinogenes S85:Fibrobacter:Fibrobacteraceae:Fibrobacterales:Fibrobacteres:Bacteria |
| Pseudomonas_fluorescens_F113 | NC_016830.1 | Pseudomonas fluorescens F113:Pseudomonas:Pseudomonadaceae:Pseudomonadales:Gammaproteobacteria:Proteobacteria:Bacteria |
| Coxiella_burnetii_CbuK_Q154 | NC_011528.1 | Coxiella burnetii CbuK_Q154:Coxiella:Coxiellaceae:Legionellales:Gammaproteobacteria:Proteobacteria:Bacteria |
| Beutenbergia_cavernae_DSM_12333 | NC_012669.1 | Beutenbergia cavernae DSM 12333:Beutenbergia:Beutenbergiaceae:Micrococcineae:Actinomycetales:Actinobacteridae:Actinobacteria:Bacteria |
| Anaplasma_phagocytophilum_HZ | NC_007797.1 | Anaplasma phagocytophilum HZ:phagocytophilum group:Anaplasma:Anaplasmataceae:Rickettsiales:Alphaproteobacteria:Proteobacteria:Bacteria |
| Acidaminococcus_fermentans_DSM_20731 | NC_013740.1 | Acidaminococcus fermentans DSM 20731:Acidaminococcus:Acidaminococcaceae:Selenomonadales:Negativicutes:Firmicutes:Bacteria |
| Rubrobacter_xylanophilus_DSM_9941 | NC_008148.1 | Rubrobacter xylanophilus DSM 9941:Rubrobacter:Rubrobacteraceae:Rubrobacterineae:Rubrobacterales:Rubrobacteridae:Actinobacteria:Bacteria |
| Coprothermobacter_proteolyticus_DSM_5265 | NC_011295.1 | Coprothermobacter proteolyticus DSM 5265:Coprothermobacter:Thermodesulfobiaceae:Thermoanaerobacterales:Clostridia:Firmicutes:Bacteria |
| Anoxybacillus_flavithermus_WK1 | NC_011567.1 | Anoxybacillus flavithermus WK1:Anoxybacillus:Bacillaceae:Bacillales:Firmicutes:Bacteria |
| Gallibacterium_anatis_UMN179 | NC_015460.1 | Gallibacterium anatis UMN179:Gallibacterium:Pasteurellaceae:Pasteurellales:Gammaproteobacteria:Proteobacteria:Bacteria |
| Chitinophaga_pinensis_DSM_2588 | NC_013132.1 | Chitinophaga pinensis DSM 2588:Chitinophaga:Chitinophagaceae:Sphingobacteriales:Sphingobacteriia:Bacteroidetes:Bacteria |
| Rahnella_aquatilis_HX2 | NC_017047.1 | Rahnella aquatilis HX2:Rahnella:Enterobacteriaceae:Enterobacteriales:Gammaproteobacteria:Proteobacteria:Bacteria |
| Leifsonia_xyli_subsp_xyli_str_CTCB07 | NC_006087.1 | Leifsonia xyli subsp. xyli str. CTCB07:Leifsonia:Microbacteriaceae:Micrococcineae:Actinomycetales:Actinobacteridae:Actinobacteria:Bacteria |
| Bacillus_licheniformis_DSM_13 | NC_006270.3 | Bacillus licheniformis DSM 13 = ATCC 14580:Bacillus:Bacillaceae:Bacillales:Firmicutes:Bacteria |
| Campylobacter_lari_RM2100 | NC_012039.1 | Campylobacter lari RM2100:Campylobacter:Campylobacteraceae:Campylobacterales:Epsilonproteobacteria:Proteobacteria:Bacteria |
| Baumannia_cicadellinicola_str_Hc_Homalodisca_coagulata | NC_007984.1 | Baumannia cicadellinicola str. Hc (Homalodisca coagulata):Candidatus Baumannia:Gammaproteobacteria:Proteobacteria:Bacteria |
| Prevotella_denticola_F0289 | NC_015311.1 | Prevotella denticola F0289:Prevotella:Prevotellaceae:Bacteroidales:Bacteroidia:Bacteroidetes:Bacteria |
| Staphylococcus_lugdunensis_HKU09_01 | NC_013893.1 | Staphylococcus lugdunensis HKU09-01:Staphylococcus:Bacillales:Firmicutes:Bacteria |
| Bartonella_quintana_str_Toulouse | NC_005955.1 | Bartonella quintana str. Toulouse:Bartonella:Bartonellaceae:Rhizobiales:Alphaproteobacteria:Proteobacteria:Bacteria |
| Atopobium_parvulum_DSM_20469 | NC_013203.1 | Atopobium parvulum DSM 20469:Atopobium:Coriobacteriaceae:Coriobacterineae:Coriobacteriales:Coriobacteridae:Actinobacteria:Bacteria |
| Thermosediminibacter_oceani_DSM_16646 | NC_014377.1 | Thermosediminibacter oceani DSM 16646:Thermosediminibacter:Incertae Sedis:Thermoanaerobacterales Family III:Thermoanaerobacterales:Clostridia:Firmicutes:Bacteria |
| Nitrosococcus_watsonii_C_113 | NC_014315.1 | Nitrosococcus watsonii C-113:Nitrosococcus:Chromatiaceae:Chromatiales:Gammaproteobacteria:Proteobacteria:Bacteria |
| Erwinia_amylovora_CFBP1430 | NC_013961.1 | Erwinia amylovora CFBP1430:Erwinia:Enterobacteriaceae:Enterobacteriales:Gammaproteobacteria:Proteobacteria:Bacteria |
| Desulfovibrio_magneticus_RS_1 | NC_012796.1 | Desulfovibrio magneticus RS-1:Desulfovibrio:Desulfovibrionaceae:Desulfovibrionales:Deltaproteobacteria:Proteobacteria:Bacteria |
| Rickettsia_felis_URRWXCal2 | NC_007109.1 | Rickettsia felis URRWXCal2:spotted fever group:Rickettsia:Rickettsieae:Rickettsiaceae:Rickettsiales:Alphaproteobacteria:Proteobacteria:Bacteria |
| Streptococcus_equi_subsp_zooepidemicus | NC_012470.1 | Streptococcus equi subsp. zooepidemicus:Streptococcus:Streptococcaceae:Lactobacillales:Firmicutes:Bacteria |
| Calditerrivibrio_nitroreducens_DSM_19672 | NC_014758.1 | Calditerrivibrio nitroreducens DSM 19672:Deferribacteraceae:Deferribacterales:Deferribacteres:Bacteria |
| Oligotropha_carboxidovorans_OM5 | NC_015684.1 | Oligotropha carboxidovorans OM5:Oligotropha:Bradyrhizobiaceae:Rhizobiales:Alphaproteobacteria:Proteobacteria:Bacteria |
| Robiginitalea_biformata_HTCC2501 | NC_013222.1 | Robiginitalea biformata HTCC2501:Robiginitalea:Flavobacteriaceae:Flavobacteriales:Flavobacteriia:Bacteroidetes:Bacteria |
| Helicobacter_cinaedi_PAGU611 | NC_017761.1 | Helicobacter cinaedi PAGU611:Helicobacter:Helicobacteraceae:Campylobacterales:Epsilonproteobacteria:Proteobacteria:Bacteria |
| Marivirga_tractuosa_DSM_4126 | NC_014759.1 | Marivirga tractuosa DSM 4126:Marivirga:Flammeovirgaceae:Cytophagales:Cytophagia:Bacteroidetes:Bacteria |
| Anaeromyxobacter_dehalogenans_2CP_1 | NC_011891.1 | Anaeromyxobacter dehalogenans 2CP-1:Anaeromyxobacter:Myxococcaceae:Cystobacterineae:Myxococcales:Deltaproteobacteria:Proteobacteria:Bacteria |
| Coxiella_burnetii_CbuG_Q212 | NC_011527.1 | Coxiella burnetii CbuG_Q212:Coxiella:Coxiellaceae:Legionellales:Gammaproteobacteria:Proteobacteria:Bacteria |
| Leptotrichia_buccalis_C_1013_b | NC_013192.1 | Leptotrichia buccalis C-1013-b:Leptotrichia:Leptotrichiaceae:Fusobacteriales:Fusobacteria:Bacteria |
| Syntrophobacter_fumaroxidans_MPOB | NC_008554.1 | Syntrophobacter fumaroxidans MPOB:Syntrophobacter:Syntrophobacteraceae:Syntrophobacterales:Deltaproteobacteria:Proteobacteria:Bacteria |
| Lactobacillus_delbrueckii_subsp_bulgaricus_ATCC_11842 | NC_008054.1 | Lactobacillus delbrueckii subsp. bulgaricus ATCC 11842:Lactobacillus:Lactobacillaceae:Lactobacillales:Firmicutes:Bacteria |
| Flavobacteriaceae_bacterium_3519_10 | NC_013062.1 | Flavobacteriaceae bacterium 3519-10:Flavobacteriaceae:Flavobacteriales:Flavobacteriia:Bacteroidetes:Bacteria |
| Cellulophaga_algicola_DSM_14237 | NC_014934.1 | Cellulophaga algicola DSM 14237:Cellulophaga:Flavobacteriaceae:Flavobacteriales:Flavobacteriia:Bacteroidetes:Bacteria |
| Chlorobium_luteolum_DSM_273 | NC_007512.1 | Chlorobium luteolum DSM 273:Pelodictyon:Chlorobium/Pelodictyon group:Chlorobiaceae:Chlorobiales:Chlorobia:Chlorobi:Bacteria |
| Nautilia_profundicola_AmH | NC_012115.1 | Nautilia profundicola AmH:Nautilia:Nautiliaceae:Nautiliales:Epsilonproteobacteria:Proteobacteria:Bacteria |
| Burkholderia_sp_CCGE1001 | NC_015137.1 | Burkholderia sp. CCGE1001:Burkholderia:Burkholderiaceae:Burkholderiales:Betaproteobacteria:Proteobacteria:Bacteria |
| Aggregatibacter_actinomycetemcomitans_ANH9381 | NC_016513.1 | Aggregatibacter actinomycetemcomitans ANH9381:Aggregatibacter:Pasteurellaceae:Pasteurellales:Gammaproteobacteria:Proteobacteria:Bacteria |
| Leuconostoc_kimchii_IMSNU_11154 | NC_014136.1 | Leuconostoc kimchii IMSNU 11154:Leuconostoc:Lactobacillales:Firmicutes:Bacteria |
| Herbaspirillum_seropedicae_SmR1 | NC_014323.1 | Herbaspirillum seropedicae SmR1:Herbaspirillum:Oxalobacteraceae:Burkholderiales:Betaproteobacteria:Proteobacteria:Bacteria |
| Streptococcus_gallolyticus_subsp_gallolyticus_ATCC_BAA_2069 | NC_015215.1 | Streptococcus gallolyticus subsp. gallolyticus ATCC BAA-2069:Streptococcus:Streptococcaceae:Lactobacillales:Firmicutes:Bacteria |
| Vibrio_fischeri_MJ11 | NC_011186.1 | Vibrio fischeri MJ11:Aliivibrio:Vibrionaceae:Vibrionales:Gammaproteobacteria:Proteobacteria:Bacteria |
| Francisella_sp_TX077308 | NC_015696.1 | Francisella sp. TX077308:Francisella:Francisellaceae:Thiotrichales:Gammaproteobacteria:Proteobacteria:Bacteria |
| Rhodospirillum_photometricum | NC_017059.1 | Rhodospirillum photometricum DSM 122:Rhodospirillum:Rhodospirillaceae:Rhodospirillales:Alphaproteobacteria:Proteobacteria:Bacteria |
| Methylobacterium_radiotolerans_JCM_2831 | NC_010505.1 | Methylobacterium radiotolerans JCM 2831:Methylobacterium:Methylobacteriaceae:Rhizobiales:Alphaproteobacteria:Proteobacteria:Bacteria |
| Legionella_longbeachae_NSW150 | NC_013861.1 | Legionella longbeachae NSW150:Legionella:Legionellaceae:Legionellales:Gammaproteobacteria:Proteobacteria:Bacteria |
| Pseudomonas_fulva_12_X | NC_015556.1 | Pseudomonas fulva 12-X:Pseudomonas:Pseudomonadaceae:Pseudomonadales:Gammaproteobacteria:Proteobacteria:Bacteria |
| Desulfovibrio_vulgaris_str_Hildenborough | NC_002937.3 | Desulfovibrio vulgaris str. Hildenborough:Desulfovibrio:Desulfovibrionaceae:Desulfovibrionales:Deltaproteobacteria:Proteobacteria:Bacteria |
| Borrelia_duttonii_Ly | NC_011229.1 | Borrelia duttonii Ly:Borrelia:Spirochaetaceae:Spirochaetales:Spirochaetes:Bacteria |
| Jannaschia_sp_CCS1 | NC_007802.1 | Jannaschia sp. CCS1:Jannaschia:Rhodobacteraceae:Rhodobacterales:Alphaproteobacteria:Proteobacteria:Bacteria |
| Butyrivibrio_proteoclasticus_B316 | NC_014388.1 | Butyrivibrio proteoclasticus B316:Butyrivibrio:Lachnospiraceae:Clostridiales:Clostridia:Firmicutes:Bacteria |
| Mycobacterium_marinum_M | NC_010612.1 | Mycobacterium marinum M:Mycobacterium:Mycobacteriaceae:Corynebacterineae:Actinomycetales:Actinobacteridae:Actinobacteria:Bacteria |
| Streptococcus_pneumoniae_670_6B | NC_014498.1 | Streptococcus pneumoniae 670-6B:Streptococcus:Streptococcaceae:Lactobacillales:Firmicutes:Bacteria |
| Providencia_stuartii_MRSN_2154 | NC_017731.1 | Providencia stuartii MRSN 2154:Providencia:Enterobacteriaceae:Enterobacteriales:Gammaproteobacteria:Proteobacteria:Bacteria |
| Mycobacterium_intracellulare_MOTT_02 | NC_016947.1 | Mycobacterium intracellulare MOTT-02:Mycobacterium avium complex (MAC):Mycobacterium:Mycobacteriaceae:Corynebacterineae:Actinomycetales:Actinobacteridae:Actinobacteria:Bacteria |
| Pseudomonas_entomophila_L48 | NC_008027.1 | Pseudomonas entomophila L48:Pseudomonas:Pseudomonadaceae:Pseudomonadales:Gammaproteobacteria:Proteobacteria:Bacteria |
| Ehrlichia_chaffeensis_str_Arkansas | NC_007799.1 | Ehrlichia chaffeensis str. Arkansas:Ehrlichia:Anaplasmataceae:Rickettsiales:Alphaproteobacteria:Proteobacteria:Bacteria |
| Leptospirillum_ferrooxidans_C2_3 | NC_017094.1 | Leptospirillum ferrooxidans C2-3:Leptospirillum:Nitrospiraceae:Nitrospirales:Nitrospirae:Bacteria |
| Pseudoalteromonas_atlantica_T6c | NC_008228.1 | Pseudoalteromonas atlantica T6c:Pseudoalteromonas:Pseudoalteromonadaceae:Alteromonadales:Gammaproteobacteria:Proteobacteria:Bacteria |
| Paenibacillus_mucilaginosus_3016 | NC_016935.1 | Paenibacillus mucilaginosus 3016:Paenibacillus:Paenibacillaceae:Bacillales:Firmicutes:Bacteria |
| Burkholderia_gladioli_BSR3 | NC_015381.1 | Burkholderia gladioli BSR3:Burkholderia:Burkholderiaceae:Burkholderiales:Betaproteobacteria:Proteobacteria:Bacteria |
| Oenococcus_oeni_PSU_1 | NC_008528.1 | Oenococcus oeni PSU-1:Oenococcus:Lactobacillales:Firmicutes:Bacteria |
| Serratia_sp_AS12 | NC_015566.1 | Serratia sp. AS12:Serratia:Enterobacteriaceae:Enterobacteriales:Gammaproteobacteria:Proteobacteria:Bacteria |
| Caulobacter_crescentus_CB15 | NC_002696.2 | Caulobacter crescentus CB15:Caulobacter:Caulobacteraceae:Caulobacterales:Alphaproteobacteria:Proteobacteria:Bacteria |
| Lactobacillus_reuteri_DSM_20016 | NC_009513.1 | Lactobacillus reuteri DSM 20016:Lactobacillus:Lactobacillaceae:Lactobacillales:Firmicutes:Bacteria |
| Pelobacter_carbinolicus_DSM_2380 | NC_007498.2 | Pelobacter carbinolicus DSM 2380:Pelobacter:Pelobacteraceae:Desulfuromonadales:Deltaproteobacteria:Proteobacteria:Bacteria |
| Kineococcus_radiotolerans_SRS30216 | NC_009664.2 | Kineococcus radiotolerans SRS30216:Kineococcus:Kineosporiaceae:Kineosporiineae:Actinomycetales:Actinobacteridae:Actinobacteria:Bacteria |
| Campylobacter_jejuni_RM1221 | NC_003912.7 | Campylobacter jejuni RM1221:Campylobacter:Campylobacteraceae:Campylobacterales:Epsilonproteobacteria:Proteobacteria:Bacteria |
| Aggregatibacter_actinomycetemcomitans_D11S_1 | NC_013597.1 | Aggregatibacter phage S1249:Myoviridae:Caudovirales:dsDNA viruses, no RNA stage:Viruses |
| Vibrio_vulnificus_MO6_24_O | NC_014966.1 | Vibrio vulnificus MO6-24/O:Vibrio:Vibrionaceae:Vibrionales:Gammaproteobacteria:Proteobacteria:Bacteria |
| Pseudogulbenkiania_sp_NH8B | NC_016002.1 | Pseudogulbenkiania sp. NH8B:Pseudogulbenkiania:Neisseriaceae:Neisseriales:Betaproteobacteria:Proteobacteria:Bacteria |
| Bacteriovorax_marinus_SJ | NC_016620.1 | Bacteriovorax marinus SJ:Bacteriovorax:Bacteriovoracaceae:Bdellovibrionales:Deltaproteobacteria:Proteobacteria:Bacteria |
| Caldicellulosiruptor_lactoaceticus_6A | NC_015949.1 | Caldicellulosiruptor lactoaceticus 6A:Caldicellulosiruptor:Incertae Sedis:Thermoanaerobacterales Family III:Thermoanaerobacterales:Clostridia:Firmicutes:Bacteria |
| Caldicellulosiruptor_obsidiansis_OB47 | NC_014392.1 | Caldicellulosiruptor obsidiansis OB47:Caldicellulosiruptor:Incertae Sedis:Thermoanaerobacterales Family III:Thermoanaerobacterales:Clostridia:Firmicutes:Bacteria |
| Anaeromyxobacter_dehalogenans_2CP_C | NC_007760.1 | Anaeromyxobacter dehalogenans 2CP-C:Anaeromyxobacter:Myxococcaceae:Cystobacterineae:Myxococcales:Deltaproteobacteria:Proteobacteria:Bacteria |
| Serratia_sp_AS13 | NC_017573.1 | Serratia sp. AS13:Serratia:Enterobacteriaceae:Enterobacteriales:Gammaproteobacteria:Proteobacteria:Bacteria |
| Methylobacterium_extorquens_DM4 | NC_012988.1 | Methylobacterium extorquens DM4:Methylobacterium:Methylobacteriaceae:Rhizobiales:Alphaproteobacteria:Proteobacteria:Bacteria |
| Geobacter_uraniireducens_Rf4 | NC_009483.1 | Geobacter uraniireducens Rf4:Geobacter:Geobacteraceae:Desulfuromonadales:Deltaproteobacteria:Proteobacteria:Bacteria |
| Porphyromonas_gingivalis_TDC60 | NC_015571.1 | Porphyromonas gingivalis TDC60:Porphyromonas:Porphyromonadaceae:Bacteroidales:Bacteroidia:Bacteroidetes:Bacteria |
| Klebsiella_variicola_At_22 | NC_013850.1 | Klebsiella variicola At-22:Klebsiella:Enterobacteriaceae:Enterobacteriales:Gammaproteobacteria:Proteobacteria:Bacteria |
| Rickettsia_rickettsii_str_Arizona | NC_016909.1 | Rickettsia rickettsii str. Arizona:spotted fever group:Rickettsia:Rickettsieae:Rickettsiaceae:Rickettsiales:Alphaproteobacteria:Proteobacteria:Bacteria |
| Staphylococcus_saprophyticus_subsp_saprophyticus_ATCC_15305 | NC_007350.1 | Staphylococcus saprophyticus subsp. saprophyticus ATCC 15305:Staphylococcus:Bacillales:Firmicutes:Bacteria |
| Rickettsia_bellii_OSU_85_389 | NC_009883.1 | Rickettsia bellii OSU 85-389:belli group:Rickettsia:Rickettsieae:Rickettsiaceae:Rickettsiales:Alphaproteobacteria:Proteobacteria:Bacteria |
| Azotobacter_vinelandii_DJ | NC_012560.1 | Azotobacter vinelandii DJ:Azotobacter:Pseudomonadaceae:Pseudomonadales:Gammaproteobacteria:Proteobacteria:Bacteria |
| Salinispora_arenicola_CNS_205 | NC_009953.1 | Salinispora arenicola CNS-205:Salinispora:Micromonosporaceae:Micromonosporineae:Actinomycetales:Actinobacteridae:Actinobacteria:Bacteria |
| Rhodobacter_sphaeroides_2.4.1 | NC_007494.1 | Rhodobacter sphaeroides 2.4.1:Rhodobacter:Rhodobacteraceae:Rhodobacterales:Alphaproteobacteria:Proteobacteria:Bacteria |
| Asticcacaulis_excentricus_CB_48 | NC_014817.1 | Asticcacaulis excentricus CB 48:Asticcacaulis:Caulobacteraceae:Caulobacterales:Alphaproteobacteria:Proteobacteria:Bacteria |
| Pelobacter_propionicus_DSM_2379 | NC_008609.1 | Pelobacter propionicus DSM 2379:Pelobacter:Pelobacteraceae:Desulfuromonadales:Deltaproteobacteria:Proteobacteria:Bacteria |
| Corynebacterium_ulcerans_BR_AD22 | NC_015683.1 | Corynebacterium ulcerans BR-AD22:Corynebacterium:Corynebacteriaceae:Corynebacterineae:Actinomycetales:Actinobacteridae:Actinobacteria:Bacteria |
| Frankia_sp_CcI3 | NC_007777.1 | Frankia sp. CcI3:Frankia:Frankiaceae:Frankineae:Actinomycetales:Actinobacteridae:Actinobacteria:Bacteria |
| Shewanella_oneidensis_MR_1 | NC_004347.1 | Shewanella oneidensis MR-1:Shewanella:Shewanellaceae:Alteromonadales:Gammaproteobacteria:Proteobacteria:Bacteria |
| Zunongwangia_profunda_SM_A87 | NC_014041.1 | Zunongwangia profunda SM-A87:Zunongwangia:Flavobacteriaceae:Flavobacteriales:Flavobacteriia:Bacteroidetes:Bacteria |
| Desulfotalea_psychrophila_LSv54 | NC_006138.1 | Desulfotalea psychrophila LSv54:Desulfotalea:Desulfobulbaceae:Desulfobacterales:Deltaproteobacteria:Proteobacteria:Bacteria |
| Paludibacter_propionicigenes_WB4 | NC_014734.1 | Paludibacter propionicigenes WB4:Paludibacter:Porphyromonadaceae:Bacteroidales:Bacteroidia:Bacteroidetes:Bacteria |
| Candidatus_Sulcia_muelleri_DMIN | NC_014004.1 | Candidatus Sulcia muelleri DMIN:Candidatus Sulcia:Flavobacteriales:Flavobacteriia:Bacteroidetes:Bacteria |
| Arcobacter_butzleri_RM4018 | NC_009850.1 | Arcobacter butzleri RM4018:Arcobacter:Campylobacteraceae:Campylobacterales:Epsilonproteobacteria:Proteobacteria:Bacteria |
| Fervidobacterium_pennivorans_DSM_9078 | NC_017095.1 | Fervidobacterium pennivorans DSM 9078:Fervidobacterium:Thermotogaceae:Thermotogales:Thermotogae:Bacteria |
| Nitrosomonas_sp_Is79A3 | NC_015731.1 | Nitrosomonas sp. Is79A3:Nitrosomonas:Nitrosomonadaceae:Nitrosomonadales:Betaproteobacteria:Proteobacteria:Bacteria |
| Fluviicola_taffensis_DSM_16823 | NC_015321.1 | Fluviicola taffensis DSM 16823:Fluviicola:Cryomorphaceae:Flavobacteriales:Flavobacteriia:Bacteroidetes:Bacteria |
| Lactobacillus_kefiranofaciens_ZW3 | NC_015602.1 | Lactobacillus kefiranofaciens ZW3:Lactobacillus:Lactobacillaceae:Lactobacillales:Firmicutes:Bacteria |
| Sinorhizobium_fredii | NC_016812.1 | Sinorhizobium fredii HH103:Sinorhizobium:Sinorhizobium/Ensifer group:Rhizobiaceae:Rhizobiales:Alphaproteobacteria:Proteobacteria:Bacteria |
| Thermobaculum_terrenum_ATCC_BAA_798 | NC_013526.1 | Thermobaculum terrenum ATCC BAA-798:Thermobaculum:Bacteria |
| Micromonospora_aurantiaca_ATCC_27029 | NC_014391.1 | Micromonospora aurantiaca ATCC 27029:Micromonospora:Micromonosporaceae:Micromonosporineae:Actinomycetales:Actinobacteridae:Actinobacteria:Bacteria |
| Acetohalobium_arabaticum_DSM_5501 | NC_014378.1 | Acetohalobium arabaticum DSM 5501:Acetohalobium:Halobacteroidaceae:Halanaerobiales:Clostridia:Firmicutes:Bacteria |
| Marinomonas_mediterranea_MMB_1 | NC_015276.1 | Marinomonas mediterranea MMB-1:Marinomonas:Oceanospirillales:Gammaproteobacteria:Proteobacteria:Bacteria |
| Dehalococcoides_sp_VS | NC_013552.1 | Dehalococcoides sp. VS:Dehalococcoides:Dehalococcoidetes:Chloroflexi:Bacteria |
| Mycobacterium_canettii_CIPT_140010059 | NC_015848.1 | Mycobacterium canettii CIPT 140010059:Mycobacterium tuberculosis complex:Mycobacterium:Mycobacteriaceae:Corynebacterineae:Actinomycetales:Actinobacteridae:Actinobacteria:Bacteria |
| Clostridium_beijerinckii_NCIMB_8052 | NC_009617.1 | Clostridium beijerinckii NCIMB 8052:Clostridium:Clostridiaceae:Clostridiales:Clostridia:Firmicutes:Bacteria |
| Neorickettsia_sennetsu_str_Miyayama | NC_007798.1 | Neorickettsia sennetsu str. Miyayama:Neorickettsia:Anaplasmataceae:Rickettsiales:Alphaproteobacteria:Proteobacteria:Bacteria |
| Wigglesworthia_glossinidia_endosymbiont_of_Glossina_morsitans_morsitans_Yale_colony | NC_016893.1 | Wigglesworthia glossinidia endosymbiont of Glossina morsitans morsitans (Yale colony):Wigglesworthia:Enterobacteriaceae:Enterobacteriales:Gammaproteobacteria:Proteobacteria:Bacteria |
| Propionibacterium_freudenreichii_subsp_shermanii_CIRM_BIA1 | NC_014215.1 | Propionibacterium freudenreichii subsp. shermanii CIRM-BIA1:Propionibacterium:Propionibacteriaceae:Propionibacterineae:Actinomycetales:Actinobacteridae:Actinobacteria:Bacteria |
| Lactobacillus_amylovorus_GRL_1112 | NC_014724.1 | Lactobacillus amylovorus GRL 1112:Lactobacillus:Lactobacillaceae:Lactobacillales:Firmicutes:Bacteria |
| Jonesia_denitrificans_DSM_20603 | NC_013174.1 | Jonesia denitrificans DSM 20603:Jonesia:Jonesiaceae:Micrococcineae:Actinomycetales:Actinobacteridae:Actinobacteria:Bacteria |
| Shewanella_denitrificans_OS217 | NC_007954.1 | Shewanella denitrificans OS217:Shewanella:Shewanellaceae:Alteromonadales:Gammaproteobacteria:Proteobacteria:Bacteria |
| Mycobacterium_leprae_Br4923 | NC_011896.1 | Mycobacterium leprae Br4923:Mycobacterium:Mycobacteriaceae:Corynebacterineae:Actinomycetales:Actinobacteridae:Actinobacteria:Bacteria |
| Desulfobulbus_propionicus_DSM_2032 | NC_014972.1 | Desulfobulbus propionicus DSM 2032:Desulfobulbus:Desulfobulbaceae:Desulfobacterales:Deltaproteobacteria:Proteobacteria:Bacteria |
| Bacillus_cereus_03BB102 | NC_012472.1 | Bacillus cereus 03BB102:Bacillus cereus group:Bacillus:Bacillaceae:Bacillales:Firmicutes:Bacteria |
| Pectobacterium_carotovorum_subsp_carotovorum_PC1 | NC_012917.1 | Pectobacterium carotovorum subsp. carotovorum PC1:Pectobacterium:Enterobacteriaceae:Enterobacteriales:Gammaproteobacteria:Proteobacteria:Bacteria |
| Cronobacter_sakazakii_ATCC_BAA_894 | NC_009778.1 | Cronobacter sakazakii ATCC BAA-894:Cronobacter:Enterobacteriaceae:Enterobacteriales:Gammaproteobacteria:Proteobacteria:Bacteria |
| Lactobacillus_fermentum_IFO_3956 | NC_010610.1 | Lactobacillus fermentum IFO 3956:Lactobacillus:Lactobacillaceae:Lactobacillales:Firmicutes:Bacteria |
| Francisella_cf_novicida_Fx1 | NC_017450.1 | Francisella cf. novicida Fx1:Francisella:Francisellaceae:Thiotrichales:Gammaproteobacteria:Proteobacteria:Bacteria |
| Psychromonas_ingrahamii_37 | NC_008709.1 | Psychromonas ingrahamii 37:Psychromonas:Psychromonadaceae:Alteromonadales:Gammaproteobacteria:Proteobacteria:Bacteria |
| Allochromatium_vinosum_DSM_180 | NC_013851.1 | Allochromatium vinosum DSM 180:Allochromatium:Chromatiaceae:Chromatiales:Gammaproteobacteria:Proteobacteria:Bacteria |
| Actinobacillus_succinogenes_130Z | NC_009655.1 | Actinobacillus succinogenes 130Z:Actinobacillus:Pasteurellaceae:Pasteurellales:Gammaproteobacteria:Proteobacteria:Bacteria |
| Vibrio_vulnificus_CMCP6 | NC_004460.2 | Vibrio vulnificus CMCP6:Vibrio:Vibrionaceae:Vibrionales:Gammaproteobacteria:Proteobacteria:Bacteria |
| Campylobacter_fetus_subsp_fetus_82_40 | NC_008599.1 | Campylobacter fetus subsp. fetus 82-40:Campylobacter:Campylobacteraceae:Campylobacterales:Epsilonproteobacteria:Proteobacteria:Bacteria |
| Pseudoxanthomonas_suwonensis_11_1 | NC_014924.1 | Pseudoxanthomonas suwonensis 11-1:Pseudoxanthomonas:Xanthomonadaceae:Xanthomonadales:Gammaproteobacteria:Proteobacteria:Bacteria |
| Mycobacterium_smegmatis_str_MC2_155 | NC_008596.1 | Mycobacterium smegmatis str. MC2 155:Mycobacterium:Mycobacteriaceae:Corynebacterineae:Actinomycetales:Actinobacteridae:Actinobacteria:Bacteria |
| Syntrophomonas_wolfei_subsp_wolfei_str_Goettingen | NC_008346.1 | Syntrophomonas wolfei subsp. wolfei str. Goettingen:Syntrophomonas:Syntrophomonadaceae:Clostridiales:Clostridia:Firmicutes:Bacteria |
| Slackia_heliotrinireducens_DSM_20476 | NC_013165.1 | Slackia heliotrinireducens DSM 20476:Slackia:Coriobacteriaceae:Coriobacterineae:Coriobacteriales:Coriobacteridae:Actinobacteria:Bacteria |
| Corynebacterium_glutamicum_ATCC_13032 | NC_003450.3 | Corynebacterium glutamicum ATCC 13032:Corynebacterium:Corynebacteriaceae:Corynebacterineae:Actinomycetales:Actinobacteridae:Actinobacteria:Bacteria |
| Bacillus_halodurans_C_125 | NC_002570.2 | Bacillus halodurans C-125:Bacillus:Bacillaceae:Bacillales:Firmicutes:Bacteria |
| Vibrio_cholerae_IEC224 | NC_016945.1 | Vibrio cholerae IEC224:Vibrio:Vibrionaceae:Vibrionales:Gammaproteobacteria:Proteobacteria:Bacteria |
| Muricauda_ruestringensis_DSM_13258 | NC_015945.1 | Muricauda ruestringensis DSM 13258:Muricauda:Flavobacteriaceae:Flavobacteriales:Flavobacteriia:Bacteroidetes:Bacteria |
| Brucella_melitensis_ATCC_23457 | NC_012442.1 | Brucella melitensis ATCC 23457:Brucella:Brucellaceae:Rhizobiales:Alphaproteobacteria:Proteobacteria:Bacteria |
| Rickettsia_heilongjiangensis_054 | NC_015866.1 | Rickettsia heilongjiangensis 054:spotted fever group:Rickettsia:Rickettsieae:Rickettsiaceae:Rickettsiales:Alphaproteobacteria:Proteobacteria:Bacteria |
| Taylorella_equigenitalis_MCE9 | NC_014914.1 | Taylorella equigenitalis MCE9:Taylorella:Alcaligenaceae:Burkholderiales:Betaproteobacteria:Proteobacteria:Bacteria |
| Mesorhizobium_loti_MAFF303099 | NC_002678.2 | Mesorhizobium loti MAFF303099:Mesorhizobium:Phyllobacteriaceae:Rhizobiales:Alphaproteobacteria:Proteobacteria:Bacteria |
| Yersinia_pestis_Angola | NC_010159.1 | Yersinia pestis Angola:Yersinia:Enterobacteriaceae:Enterobacteriales:Gammaproteobacteria:Proteobacteria:Bacteria |
| Orientia_tsutsugamushi_str_Boryong | NC_009488.1 | Orientia tsutsugamushi str. Boryong:Orientia:Rickettsieae:Rickettsiaceae:Rickettsiales:Alphaproteobacteria:Proteobacteria:Bacteria |
| Streptococcus_mutans_UA159 | NC_004350.2 | Streptococcus mutans UA159:Streptococcus:Streptococcaceae:Lactobacillales:Firmicutes:Bacteria |
| Clostridium_tetani_E88 | NC_004557.1 | Clostridium tetani E88:Clostridium:Clostridiaceae:Clostridiales:Clostridia:Firmicutes:Bacteria |
| Geobacter_sulfurreducens_KN400 | NC_017454.1 | Geobacter sulfurreducens KN400:Geobacter:Geobacteraceae:Desulfuromonadales:Deltaproteobacteria:Proteobacteria:Bacteria |
| Sulfurihydrogenibium_azorense_Az_Fu1 | NC_012438.1 | Sulfurihydrogenibium azorense Az-Fu1:Sulfurihydrogenibium:Hydrogenothermaceae:Aquificales:Aquificae:Bacteria |
| Geobacillus_kaustophilus_HTA426 | NC_006510.1 | Geobacillus kaustophilus HTA426:Geobacillus:Bacillaceae:Bacillales:Firmicutes:Bacteria |
| Melissococcus_plutonius_ATCC_35311 | NC_015516.1 | Melissococcus plutonius ATCC 35311:Melissococcus:Enterococcaceae:Lactobacillales:Firmicutes:Bacteria |
| Enterobacter_cloacae_SCF1 | NC_014618.1 | Enterobacter cloacae SCF1:Enterobacter cloacae complex:Enterobacter:Enterobacteriaceae:Enterobacteriales:Gammaproteobacteria:Proteobacteria:Bacteria |
| Pirellula_staleyi_DSM_6068 | NC_013720.1 | Pirellula staleyi DSM 6068:Pirellula:Planctomycetaceae:Planctomycetales:Planctomycetia:Planctomycetes:Bacteria |
| Corynebacterium_efficiens_YS_314 | NC_004369.1 | Corynebacterium efficiens YS-314:Corynebacterium:Corynebacteriaceae:Corynebacterineae:Actinomycetales:Actinobacteridae:Actinobacteria:Bacteria |
| Candidatus_Blochmannia_vafer_str_BVAF | NC_014909.1 | Candidatus Blochmannia vafer str. BVAF:Candidatus Blochmannia:ant endosymbionts:Enterobacteriaceae:Enterobacteriales:Gammaproteobacteria:Proteobacteria:Bacteria |
| Spirosoma_linguale_DSM_74 | NC_013730.1 | Spirosoma linguale DSM 74:Spirosoma:Cytophagaceae:Cytophagales:Cytophagia:Bacteroidetes:Bacteria |
| Sinorhizobium_medicae_WSM419 | NC_009636.1 | Sinorhizobium medicae WSM419:Sinorhizobium:Sinorhizobium/Ensifer group:Rhizobiaceae:Rhizobiales:Alphaproteobacteria:Proteobacteria:Bacteria |
| Rickettsia_akari_str_Hartford | NC_009881.1 | Rickettsia akari str. Hartford:spotted fever group:Rickettsia:Rickettsieae:Rickettsiaceae:Rickettsiales:Alphaproteobacteria:Proteobacteria:Bacteria |
| Pantoea_ananatis | NC_016816.1 | Pantoea ananatis LMG 5342:Pantoea:Enterobacteriaceae:Enterobacteriales:Gammaproteobacteria:Proteobacteria:Bacteria |
| Psychrobacter_sp_PRwf_1 | NC_009524.1 | Psychrobacter sp. PRwf-1:Psychrobacter:Moraxellaceae:Pseudomonadales:Gammaproteobacteria:Proteobacteria:Bacteria |
| Staphylococcus_pseudintermedius_ED99 | NC_017568.1 | Staphylococcus pseudintermedius ED99:Staphylococcus:Bacillales:Firmicutes:Bacteria |
| Rhodococcus_opacus_B4 | NC_012522.1 | Rhodococcus opacus B4:Rhodococcus:Nocardiaceae:Corynebacterineae:Actinomycetales:Actinobacteridae:Actinobacteria:Bacteria |
| Hydrogenobacter_thermophilus_TK_6 | NC_017161.1 | Hydrogenobacter thermophilus TK-6:Hydrogenobacter:Aquificaceae:Aquificales:Aquificae:Bacteria |
| Magnetospirillum_magneticum_AMB_1 | NC_007626.1 | Magnetospirillum magneticum AMB-1:Magnetospirillum:Rhodospirillaceae:Rhodospirillales:Alphaproteobacteria:Proteobacteria:Bacteria |
| Pseudomonas_fluorescens_Pf0_1 | NC_007492.2 | Pseudomonas fluorescens Pf0-1:Pseudomonas:Pseudomonadaceae:Pseudomonadales:Gammaproteobacteria:Proteobacteria:Bacteria |
| Microlunatus_phosphovorus_NM_1 | NC_015635.1 | Microlunatus phosphovorus NM-1:Microlunatus:Propionibacteriaceae:Propionibacterineae:Actinomycetales:Actinobacteridae:Actinobacteria:Bacteria |
| Staphylococcus_aureus_subsp_aureus_71193 | NC_017673.1 | Staphylococcus aureus subsp. aureus 71193:Staphylococcus:Bacillales:Firmicutes:Bacteria |
| Flavobacterium_indicum_GPTSA100_9 | NC_017025.1 | Flavobacterium indicum GPTSA100-9:Flavobacterium:Flavobacteriaceae:Flavobacteriales:Flavobacteriia:Bacteroidetes:Bacteria |
| Lactobacillus_rhamnosus_GG | NC_017482.1 | Lactobacillus rhamnosus GG:Lactobacillus:Lactobacillaceae:Lactobacillales:Firmicutes:Bacteria |
| Rhizobium_leguminosarum_bv_trifolii_WSM1325 | NC_012850.1 | Rhizobium leguminosarum bv. trifolii WSM1325:Rhizobium:Rhizobium/Agrobacterium group:Rhizobiaceae:Rhizobiales:Alphaproteobacteria:Proteobacteria:Bacteria |
| Phenylobacterium_zucineum_HLK1 | NC_011144.1 | Phenylobacterium zucineum HLK1:Phenylobacterium:Caulobacteraceae:Caulobacterales:Alphaproteobacteria:Proteobacteria:Bacteria |
| Geobacter_metallireducens_GS_15 | NC_007517.1 | Geobacter metallireducens GS-15:Geobacter:Geobacteraceae:Desulfuromonadales:Deltaproteobacteria:Proteobacteria:Bacteria |
| Persephonella_marina_EX_H1 | NC_012440.1 | Persephonella marina EX-H1:Persephonella:Hydrogenothermaceae:Aquificales:Aquificae:Bacteria |
| Mycobacterium_intracellulare_ATCC_13950 | NC_016946.1 | Mycobacterium intracellulare ATCC 13950:Mycobacterium avium complex (MAC):Mycobacterium:Mycobacteriaceae:Corynebacterineae:Actinomycetales:Actinobacteridae:Actinobacteria:Bacteria |
| Salinispora_tropica_CNB_440 | NC_009380.1 | Salinispora tropica CNB-440:Salinispora:Micromonosporaceae:Micromonosporineae:Actinomycetales:Actinobacteridae:Actinobacteria:Bacteria |
| Pasteurella_multocida_subsp_multocida_str_Pm70 | NC_002663.1 | Pasteurella multocida subsp. multocida str. Pm70:Pasteurella:Pasteurellaceae:Pasteurellales:Gammaproteobacteria:Proteobacteria:Bacteria |
| Methylobacterium_chloromethanicum_CM4 | NC_011757.1 | Methylobacterium chloromethanicum CM4:Methylobacterium:Methylobacteriaceae:Rhizobiales:Alphaproteobacteria:Proteobacteria:Bacteria |
| Chlorobium_phaeovibrioides_DSM_265 | NC_009337.1 | Chlorobium phaeovibrioides DSM 265:Chlorobium:Chlorobium/Pelodictyon group:Chlorobiaceae:Chlorobiales:Chlorobia:Chlorobi:Bacteria |
| Arcobacter_nitrofigilis_DSM_7299 | NC_014166.1 | Arcobacter nitrofigilis DSM 7299:Arcobacter:Campylobacteraceae:Campylobacterales:Epsilonproteobacteria:Proteobacteria:Bacteria |
| Mycobacterium_africanum_GM041182 | NC_015758.1 | Mycobacterium africanum GM041182:Mycobacterium tuberculosis complex:Mycobacterium:Mycobacteriaceae:Corynebacterineae:Actinomycetales:Actinobacteridae:Actinobacteria:Bacteria |
| Desulfohalobium_retbaense_DSM_5692 | NC_013223.1 | Desulfohalobium retbaense DSM 5692:Desulfohalobium:Desulfohalobiaceae:Desulfovibrionales:Deltaproteobacteria:Proteobacteria:Bacteria |
| Paenibacillus_sp_Y412MC10 | NC_013406.1 | Paenibacillus sp. Y412MC10:Paenibacillus:Paenibacillaceae:Bacillales:Firmicutes:Bacteria |
| Terriglobus_saanensis_SP1PR4 | NC_014963.1 | Terriglobus saanensis SP1PR4:Terriglobus:Acidobacteriaceae:Acidobacteriales:Acidobacteria:Bacteria |
| Lactobacillus_crispatus_ST1 | NC_014106.1 | Lactobacillus crispatus ST1:Lactobacillus:Lactobacillaceae:Lactobacillales:Firmicutes:Bacteria |
| Kocuria_rhizophila_DC2201 | NC_010617.1 | Kocuria rhizophila DC2201:Kocuria:Micrococcaceae:Micrococcineae:Actinomycetales:Actinobacteridae:Actinobacteria:Bacteria |
| Chlorobium_phaeobacteroides_BS1 | NC_010831.1 | Chlorobium phaeobacteroides BS1:Chlorobium:Chlorobium/Pelodictyon group:Chlorobiaceae:Chlorobiales:Chlorobia:Chlorobi:Bacteria |
| Weissella_koreensis_KACC_15510 | NC_015759.1 | Weissella koreensis KACC 15510:Weissella:Lactobacillales:Firmicutes:Bacteria |
| Corynebacterium_urealyticum_DSM_7109 | NC_010545.1 | Corynebacterium urealyticum DSM 7109:Corynebacterium:Corynebacteriaceae:Corynebacterineae:Actinomycetales:Actinobacteridae:Actinobacteria:Bacteria |
| Erwinia_billingiae_Eb661 | NC_014306.1 | Erwinia billingiae Eb661:Erwinia:Enterobacteriaceae:Enterobacteriales:Gammaproteobacteria:Proteobacteria:Bacteria |
| Streptomyces_scabiei_87.22 | NC_013929.1 | Streptomyces scabiei 87.22:Streptomyces:Streptomycetaceae:Streptomycineae:Actinomycetales:Actinobacteridae:Actinobacteria:Bacteria |
| Serratia_proteamaculans_568 | NC_009832.1 | Serratia proteamaculans 568:Serratia:Enterobacteriaceae:Enterobacteriales:Gammaproteobacteria:Proteobacteria:Bacteria |
| Symbiobacterium_thermophilum_IAM_14863 | NC_006177.1 | Symbiobacterium thermophilum IAM 14863:Symbiobacterium:Incertae Sedis:Clostridiales Family XVIII:Clostridiales:Clostridia:Firmicutes:Bacteria |
| Streptococcus_salivarius_JIM8780 | NC_015760.1 | Streptococcus salivarius CCHSS3:Streptococcus:Streptococcaceae:Lactobacillales:Firmicutes:Bacteria |
| Aliivibrio_salmonicida_LFI1238 | NC_011313.1 | Aliivibrio salmonicida LFI1238:Aliivibrio:Vibrionaceae:Vibrionales:Gammaproteobacteria:Proteobacteria:Bacteria |
| Francisella_philomiragia_subsp_philomiragia_ATCC_25017 | NC_010336.1 | Francisella philomiragia subsp. philomiragia ATCC 25017:Francisella:Francisellaceae:Thiotrichales:Gammaproteobacteria:Proteobacteria:Bacteria |
| Brucella_canis_ATCC_23365 | NC_010104.1 | Brucella canis ATCC 23365:Brucella:Brucellaceae:Rhizobiales:Alphaproteobacteria:Proteobacteria:Bacteria |
| Caldicellulosiruptor_owensensis_OL | NC_014657.1 | Caldicellulosiruptor owensensis OL:Caldicellulosiruptor:Incertae Sedis:Thermoanaerobacterales Family III:Thermoanaerobacterales:Clostridia:Firmicutes:Bacteria |
| Clostridium_saccharolyticum_WM1 | NC_014376.1 | Clostridium saccharolyticum WM1:Clostridium:Clostridiaceae:Clostridiales:Clostridia:Firmicutes:Bacteria |
| Mycoplasma_agalactiae | NC_013948.1 | Mycoplasma agalactiae:Mycoplasma:Mycoplasmataceae:Mollicutes:Tenericutes:Bacteria |
| Arthrobacter_aurescens_TC1 | NC_008711.1 | Arthrobacter aurescens TC1:Arthrobacter:Micrococcaceae:Micrococcineae:Actinomycetales:Actinobacteridae:Actinobacteria:Bacteria |
| Caulobacter_sp_K31 | NC_010338.1 | Caulobacter sp. K31:Caulobacter:Caulobacteraceae:Caulobacterales:Alphaproteobacteria:Proteobacteria:Bacteria |
| Pseudomonas_stutzeri_A1501 | NC_009434.1 | Pseudomonas stutzeri A1501:Pseudomonas:Pseudomonadaceae:Pseudomonadales:Gammaproteobacteria:Proteobacteria:Bacteria |
| Methylotenera_mobilis_JLW8 | NC_012968.1 | Methylotenera mobilis JLW8:Methylotenera:Methylophilaceae:Methylophilales:Betaproteobacteria:Proteobacteria:Bacteria |
| Candidatus_Arthromitus_sp_SFB_rat_Yit | NC_016012.1 | Candidatus Arthromitus sp. SFB-rat-Yit:Candidatus Arthromitus:Clostridiaceae:Clostridiales:Clostridia:Firmicutes:Bacteria |
| Haliangium_ochraceum_DSM_14365 | NC_013440.1 | Haliangium ochraceum DSM 14365:Haliangium:Kofleriaceae:Nannocystineae:Myxococcales:Deltaproteobacteria:Proteobacteria:Bacteria |
| Pseudoalteromonas_haloplanktis_TAC125 | NC_007482.1 | Pseudoalteromonas haloplanktis TAC125:Pseudoalteromonas:Pseudoalteromonadaceae:Alteromonadales:Gammaproteobacteria:Proteobacteria:Bacteria |
| Planctomyces_brasiliensis_DSM_5305 | NC_015174.1 | Planctomyces brasiliensis DSM 5305:Planctomyces:Planctomycetaceae:Planctomycetales:Planctomycetia:Planctomycetes:Bacteria |
| Myxococcus_fulvus_HW_1 | NC_015711.1 | Myxococcus fulvus HW-1:Myxococcus:Myxococcaceae:Cystobacterineae:Myxococcales:Deltaproteobacteria:Proteobacteria:Bacteria |
| Candidatus_Puniceispirillum_marinum_IMCC1322 | NC_014010.1 | Candidatus Puniceispirillum marinum IMCC1322:Candidatus Puniceispirillum:SAR116 cluster:Alphaproteobacteria:Proteobacteria:Bacteria |
| Acidovorax_citrulli_AAC00_1 | NC_008752.1 | Acidovorax citrulli AAC00-1:Acidovorax:Comamonadaceae:Burkholderiales:Betaproteobacteria:Proteobacteria:Bacteria |
| Niastella_koreensis_GR20_10 | NC_016609.1 | Niastella koreensis GR20-10:Niastella:Chitinophagaceae:Sphingobacteriales:Sphingobacteriia:Bacteroidetes:Bacteria |
| Thiomonas_intermedia_K12 | NC_014153.1 | Thiomonas intermedia K12:Thiomonas:Burkholderiales:Betaproteobacteria:Proteobacteria:Bacteria |
| Moraxella_catarrhalis_RH4 | NC_014147.1 | Moraxella catarrhalis RH4:Moraxella:Moraxellaceae:Pseudomonadales:Gammaproteobacteria:Proteobacteria:Bacteria |
| Chromohalobacter_salexigens_DSM_3043 | NC_007963.1 | Chromohalobacter salexigens DSM 3043:Chromohalobacter:Halomonadaceae:Oceanospirillales:Gammaproteobacteria:Proteobacteria:Bacteria |
| Actinobacillus_pleuropneumoniae_serovar_3_str_JL03 | NC_010278.1 | Actinobacillus pleuropneumoniae serovar 3 str. JL03:Actinobacillus:Pasteurellaceae:Pasteurellales:Gammaproteobacteria:Proteobacteria:Bacteria |
| Sulfurimonas_denitrificans_DSM_1251 | NC_007575.1 | Sulfurimonas denitrificans DSM 1251:Sulfurimonas:Helicobacteraceae:Campylobacterales:Epsilonproteobacteria:Proteobacteria:Bacteria |
| Gluconacetobacter_diazotrophicus_PAl_5 | NC_010125.1 | Gluconacetobacter diazotrophicus PAl 5:Gluconacetobacter:Acetobacteraceae:Rhodospirillales:Alphaproteobacteria:Proteobacteria:Bacteria |
| Burkholderia_sp_383 | NC_007511.1 | Burkholderia sp. 383:Burkholderia cepacia complex:Burkholderia:Burkholderiaceae:Burkholderiales:Betaproteobacteria:Proteobacteria:Bacteria |
| Psychrobacter_arcticus_273_4 | NC_007204.1 | Psychrobacter arcticus 273-4:Psychrobacter:Moraxellaceae:Pseudomonadales:Gammaproteobacteria:Proteobacteria:Bacteria |
| Wolbachia_endosymbiont_of_Culex_quinquefasciatus_Pel | NC_010981.1 | Wolbachia endosymbiont of Culex quinquefasciatus Pel:Wolbachia:Wolbachieae:Anaplasmataceae:Rickettsiales:Alphaproteobacteria:Proteobacteria:Bacteria |
| Tetragenococcus_halophilus | NC_016052.1 | Tetragenococcus halophilus NBRC 12172:Tetragenococcus:Enterococcaceae:Lactobacillales:Firmicutes:Bacteria |
| Chromobacterium_violaceum_ATCC_12472 | NC_005085.1 | Chromobacterium violaceum ATCC 12472:Chromobacterium:Neisseriaceae:Neisseriales:Betaproteobacteria:Proteobacteria:Bacteria |
| Pseudovibrio_sp_FO_BEG1 | NC_016642.1 | Pseudovibrio sp. FO-BEG1:Pseudovibrio:Rhodobacteraceae:Rhodobacterales:Alphaproteobacteria:Proteobacteria:Bacteria |
| Rickettsia_slovaca_13_B | NC_016639.1 | Rickettsia slovaca 13-B:spotted fever group:Rickettsia:Rickettsieae:Rickettsiaceae:Rickettsiales:Alphaproteobacteria:Proteobacteria:Bacteria |
| Deinococcus_deserti_VCD115 | NC_012526.1 | Deinococcus deserti VCD115:Deinococcus:Deinococcaceae:Deinococcales:Deinococci:Deinococcus-Thermus:Bacteria |
| Rickettsia_japonica_YH | NC_016050.1 | Rickettsia japonica YH:spotted fever group:Rickettsia:Rickettsieae:Rickettsiaceae:Rickettsiales:Alphaproteobacteria:Proteobacteria:Bacteria |
| Hyphomicrobium_denitrificans_ATCC_51888 | NC_014313.1 | Hyphomicrobium denitrificans ATCC 51888:Hyphomicrobium:Hyphomicrobiaceae:Rhizobiales:Alphaproteobacteria:Proteobacteria:Bacteria |
| Brucella_ovis_ATCC_25840 | NC_009505.1 | Brucella ovis ATCC 25840:Brucella:Brucellaceae:Rhizobiales:Alphaproteobacteria:Proteobacteria:Bacteria |
| Erythrobacter_litoralis_HTCC2594 | NC_007722.1 | Erythrobacter litoralis HTCC2594:Erythrobacter:Erythrobacteraceae:Sphingomonadales:Alphaproteobacteria:Proteobacteria:Bacteria |
| Shewanella_putrefaciens_CN_32 | NC_009438.1 | Shewanella putrefaciens CN-32:Shewanella:Shewanellaceae:Alteromonadales:Gammaproteobacteria:Proteobacteria:Bacteria |
| Clostridium_acetobutylicum_DSM_1731 | NC_015687.1 | Clostridium acetobutylicum DSM 1731:Clostridium:Clostridiaceae:Clostridiales:Clostridia:Firmicutes:Bacteria |
| Bacillus_atrophaeus_1942 | NC_014639.1 | Bacillus atrophaeus 1942:Bacillus:Bacillaceae:Bacillales:Firmicutes:Bacteria |
| Alteromonas_sp_SN2 | NC_015554.1 | Alteromonas sp. SN2:Alteromonas:Alteromonadaceae:Alteromonadales:Gammaproteobacteria:Proteobacteria:Bacteria |
| Natranaerobius_thermophilus_JW_NM_WN_LF | NC_010718.1 | Natranaerobius thermophilus JW/NM-WN-LF:Natranaerobius:Natranaerobiaceae:Natranaerobiales:Clostridia:Firmicutes:Bacteria |
| Lactobacillus_gasseri_ATCC_33323 | NC_008530.1 | Lactobacillus gasseri ATCC 33323:Lactobacillus:Lactobacillaceae:Lactobacillales:Firmicutes:Bacteria |
| Escherichia_coli_536 | NC_008253.1 | Escherichia coli 536:Escherichia:Enterobacteriaceae:Enterobacteriales:Gammaproteobacteria:Proteobacteria:Bacteria |
| Chlorobium_phaeobacteroides_DSM_266 | NC_008639.1 | Chlorobium phaeobacteroides DSM 266:Chlorobium:Chlorobium/Pelodictyon group:Chlorobiaceae:Chlorobiales:Chlorobia:Chlorobi:Bacteria |
| Frankia_sp_EAN1pec | NC_009921.1 | Frankia sp. EAN1pec:Frankia:Frankiaceae:Frankineae:Actinomycetales:Actinobacteridae:Actinobacteria:Bacteria |
| Sphingobium_sp_SYK_6 | NC_015976.1 | Sphingobium sp. SYK-6:Sphingobium:Sphingomonadaceae:Sphingomonadales:Alphaproteobacteria:Proteobacteria:Bacteria |
| Isoptericola_variabilis_225 | NC_015588.1 | Isoptericola variabilis 225:Isoptericola:Promicromonosporaceae:Micrococcineae:Actinomycetales:Actinobacteridae:Actinobacteria:Bacteria |
| Vibrio_furnissii_NCTC_11218 | NC_016628.1 | Vibrio furnissii NCTC 11218:Vibrio:Vibrionaceae:Vibrionales:Gammaproteobacteria:Proteobacteria:Bacteria |
| Flavobacterium_branchiophilum_FL_15 | NC_016001.1 | Flavobacterium branchiophilum FL-15:Flavobacterium:Flavobacteriaceae:Flavobacteriales:Flavobacteriia:Bacteroidetes:Bacteria |
| Streptococcus_equi_subsp_equi_4047 | NC_012471.1 | Streptococcus equi subsp. equi 4047:Streptococcus:Streptococcaceae:Lactobacillales:Firmicutes:Bacteria |
| Desulfobacca_acetoxidans_DSM_11109 | NC_015388.1 | Desulfobacca acetoxidans DSM 11109:Desulfobacca:Syntrophaceae:Syntrophobacterales:Deltaproteobacteria:Proteobacteria:Bacteria |
| Streptococcus_pseudopneumoniae_IS7493 | NC_015875.1 | Streptococcus pseudopneumoniae IS7493:Streptococcus:Streptococcaceae:Lactobacillales:Firmicutes:Bacteria |
| Lactobacillus_brevis_ATCC_367 | NC_008497.1 | Lactobacillus brevis ATCC 367:Lactobacillus:Lactobacillaceae:Lactobacillales:Firmicutes:Bacteria |
| Achromobacter_xylosoxidans_A8 | NC_014640.1 | Achromobacter xylosoxidans A8:Achromobacter:Alcaligenaceae:Burkholderiales:Betaproteobacteria:Proteobacteria:Bacteria |
| Escherichia_coli_55989 | NC_011748.1 | Escherichia coli 55989:Escherichia:Enterobacteriaceae:Enterobacteriales:Gammaproteobacteria:Proteobacteria:Bacteria |
| Geobacter_sp_FRC_32 | NC_011979.1 | Geobacter sp. FRC-32:Geobacter:Geobacteraceae:Desulfuromonadales:Deltaproteobacteria:Proteobacteria:Bacteria |
| Eubacterium_limosum_KIST612 | NC_014624.1 | Eubacterium limosum KIST612:Eubacterium:Eubacteriaceae:Clostridiales:Clostridia:Firmicutes:Bacteria |
| Riemerella_anatipestifer_RA_GD | NC_017569.1 | Riemerella anatipestifer RA-GD:Riemerella:Flavobacteriaceae:Flavobacteriales:Flavobacteriia:Bacteroidetes:Bacteria |
| Cellulomonas_flavigena_DSM_20109 | NC_014151.1 | Cellulomonas flavigena DSM 20109:Cellulomonas:Cellulomonadaceae:Micrococcineae:Actinomycetales:Actinobacteridae:Actinobacteria:Bacteria |
| Alicyclobacillus_acidocaldarius_subsp_acidocaldarius_Tc_4_1 | NC_017167.1 | Alicyclobacillus acidocaldarius subsp. acidocaldarius Tc-4-1:Alicyclobacillus:Alicyclobacillaceae:Bacillales:Firmicutes:Bacteria |
| Streptococcus_parauberis_KCTC_11537 | NC_015558.1 | Streptococcus parauberis KCTC 11537:Streptococcus:Streptococcaceae:Lactobacillales:Firmicutes:Bacteria |
| Campylobacter_curvus_525.92 | NC_009715.1 | Campylobacter curvus 525.92:Campylobacter:Campylobacteraceae:Campylobacterales:Epsilonproteobacteria:Proteobacteria:Bacteria |
| Ignavibacterium_album_JCM_16511 | NC_017464.1 | Ignavibacterium album JCM 16511:Ignavibacterium:Ignavibacteriaceae:Ignavibacteriales:Ignavibacteria:Ignavibacteria:Bacteria |
| Haemophilus_influenzae_10810 | NC_016809.1 | Haemophilus influenzae 10810:Haemophilus:Pasteurellaceae:Pasteurellales:Gammaproteobacteria:Proteobacteria:Bacteria |
| Blattabacterium_sp_Mastotermes_darwiniensis_str_MADAR | NC_016146.1 | Blattabacterium sp. (Mastotermes darwiniensis) str. MADAR:Blattabacterium:Blattabacteriaceae:Flavobacteriales:Flavobacteriia:Bacteroidetes:Bacteria |
| Staphylococcus_haemolyticus_JCSC1435 | NC_007168.1 | Staphylococcus haemolyticus JCSC1435:Staphylococcus:Bacillales:Firmicutes:Bacteria |
| Colwellia_psychrerythraea_34H | NC_003910.7 | Colwellia psychrerythraea 34H:Colwellia:Colwelliaceae:Alteromonadales:Gammaproteobacteria:Proteobacteria:Bacteria |
| uncultured_Termite_group_1_bacterium_phylotype_Rs_D17 | NS_000191.1 | uncultured Termite group 1 bacterium phylotype Rs-D17:environmental samples:Elusimicrobia:Bacteria |
| Comamonas_testosteroni_CNB_2 | NC_013446.1 | Comamonas testosteroni CNB-2:Comamonas:Comamonadaceae:Burkholderiales:Betaproteobacteria:Proteobacteria:Bacteria |
| Neisseria_meningitidis_053442 | NC_010120.1 | Neisseria meningitidis 053442:Neisseria:Neisseriaceae:Neisseriales:Betaproteobacteria:Proteobacteria:Bacteria |
| Helicobacter_hepaticus_ATCC_51449 | NC_004917.1 | Helicobacter hepaticus ATCC 51449:Helicobacter:Helicobacteraceae:Campylobacterales:Epsilonproteobacteria:Proteobacteria:Bacteria |
| Granulicella_mallensis_MP5ACTX8 | NC_016631.1 | Granulicella mallensis MP5ACTX8:Granulicella:Acidobacteriaceae:Acidobacteriales:Acidobacteria:Bacteria |
| Propionibacterium_acnes_266 | NC_017534.1 | Propionibacterium acnes 266:Propionibacterium:Propionibacteriaceae:Propionibacterineae:Actinomycetales:Actinobacteridae:Actinobacteria:Bacteria |
| Capnocytophaga_ochracea_DSM_7271 | NC_013162.1 | Capnocytophaga ochracea DSM 7271:Capnocytophaga:Flavobacteriaceae:Flavobacteriales:Flavobacteriia:Bacteroidetes:Bacteria |
| Dichelobacter_nodosus_VCS1703A | NC_009446.1 | Dichelobacter nodosus VCS1703A:Dichelobacter:Cardiobacteriaceae:Cardiobacteriales:Gammaproteobacteria:Proteobacteria:Bacteria |
| Desulfurobacterium_thermolithotrophum_DSM_11699 | NC_015185.1 | Desulfurobacterium thermolithotrophum DSM 11699:Desulfurobacterium:Desulfurobacteriaceae:Aquificales:Aquificae:Bacteria |
| Oscillibacter_valericigenes | NC_016048.1 | Oscillibacter valericigenes Sjm18-20:Oscillibacter:Oscillospiraceae:Clostridiales:Clostridia:Firmicutes:Bacteria |
| Helicobacter_mustelae_12198 | NC_013949.1 | Helicobacter mustelae 12198:Helicobacter:Helicobacteraceae:Campylobacterales:Epsilonproteobacteria:Proteobacteria:Bacteria |
| Selenomonas_ruminantium_subsp_lactilytica_TAM6421 | NC_017068.1 | Selenomonas ruminantium subsp. lactilytica TAM6421:Selenomonas:Veillonellaceae:Selenomonadales:Negativicutes:Firmicutes:Bacteria |
| Escherichia_fergusonii_ATCC_35469 | NC_011740.1 | Escherichia fergusonii ATCC 35469:Escherichia:Enterobacteriaceae:Enterobacteriales:Gammaproteobacteria:Proteobacteria:Bacteria |
| Streptococcus_gallolyticus_subsp_gallolyticus_ATCC_43143 | NC_017576.1 | Streptococcus gallolyticus subsp. gallolyticus ATCC 43143:Streptococcus:Streptococcaceae:Lactobacillales:Firmicutes:Bacteria |
| Brucella_abortus_bv_1_str_9_941 | NC_006933.1 | Brucella abortus bv. 1 str. 9-941:Brucella:Brucellaceae:Rhizobiales:Alphaproteobacteria:Proteobacteria:Bacteria |
| Sphingobium_japonicum_UT26S | NC_014013.1 | Sphingobium japonicum UT26S:Sphingobium:Sphingomonadaceae:Sphingomonadales:Alphaproteobacteria:Proteobacteria:Bacteria |
| Vibrio_sp_EJY3 | NC_016614.1 | Vibrio sp. EJY3:Vibrio:Vibrionaceae:Vibrionales:Gammaproteobacteria:Proteobacteria:Bacteria |
| Granulicella_tundricola | NC_015064.1 | Granulicella tundricola:Granulicella:Acidobacteriaceae:Acidobacteriales:Acidobacteria:Bacteria |
| Aggregatibacter_aphrophilus_NJ8700 | NC_012913.1 | Aggregatibacter aphrophilus NJ8700:Aggregatibacter:Pasteurellaceae:Pasteurellales:Gammaproteobacteria:Proteobacteria:Bacteria |
| Ramlibacter_tataouinensis_TTB310 | NC_015677.1 | Ramlibacter tataouinensis TTB310:Ramlibacter:Comamonadaceae:Burkholderiales:Betaproteobacteria:Proteobacteria:Bacteria |
| Rickettsia_australis_str_Cutlack | NC_017058.1 | Rickettsia australis str. Cutlack:spotted fever group:Rickettsia:Rickettsieae:Rickettsiaceae:Rickettsiales:Alphaproteobacteria:Proteobacteria:Bacteria |
| Beijerinckia_indica_subsp_indica_ATCC_9039 | NC_010581.1 | Beijerinckia indica subsp. indica ATCC 9039:Beijerinckia:Beijerinckiaceae:Rhizobiales:Alphaproteobacteria:Proteobacteria:Bacteria |
| Polynucleobacter_necessarius_subsp_necessarius_STIR1 | NC_010531.1 | Polynucleobacter necessarius subsp. necessarius STIR1:Polynucleobacter:Burkholderiaceae:Burkholderiales:Betaproteobacteria:Proteobacteria:Bacteria |
| Flavobacterium_psychrophilum_JIP02_86 | NC_009613.1 | Flavobacterium psychrophilum JIP02/86:Flavobacterium:Flavobacteriaceae:Flavobacteriales:Flavobacteriia:Bacteroidetes:Bacteria |
| Shewanella_pealeana_ATCC_700345 | NC_009901.1 | Shewanella pealeana ATCC 700345:Shewanella:Shewanellaceae:Alteromonadales:Gammaproteobacteria:Proteobacteria:Bacteria |
| Microbacterium_testaceum_StLB037 | NC_015125.1 | Microbacterium testaceum StLB037:Microbacterium:Microbacteriaceae:Micrococcineae:Actinomycetales:Actinobacteridae:Actinobacteria:Bacteria |
| Segniliparus_rotundus_DSM_44985 | NC_014168.1 | Segniliparus rotundus DSM 44985:Segniliparus:Segniliparaceae:Corynebacterineae:Actinomycetales:Actinobacteridae:Actinobacteria:Bacteria |
| Neisseria_gonorrhoeae_NCCP11945 | NC_011035.1 | Neisseria gonorrhoeae NCCP11945:Neisseria:Neisseriaceae:Neisseriales:Betaproteobacteria:Proteobacteria:Bacteria |
| Ochrobactrum_anthropi_ATCC_49188 | NC_009668.1 | Ochrobactrum anthropi ATCC 49188:Ochrobactrum:Brucellaceae:Rhizobiales:Alphaproteobacteria:Proteobacteria:Bacteria |
| Rhodothermus_marinus_SG0.5JP17_172 | NC_015966.1 | Rhodothermus marinus SG0.5JP17-172:Rhodothermus:Rhodothermaceae:Incertae sedis:Bacteroidetes Order II:Bacteroidetes:Bacteria |
| Acidithiobacillus_ferrooxidans_ATCC_23270 | NC_011761.1 | Acidithiobacillus ferrooxidans ATCC 23270:Acidithiobacillus:Acidithiobacillaceae:Acidithiobacillales:Gammaproteobacteria:Proteobacteria:Bacteria |
| Trichodesmium_erythraeum_IMS101 | NC_008312.1 | Trichodesmium erythraeum IMS101:Trichodesmium:Oscillatoriales:Cyanobacteria:Bacteria |
| Lacinutrix_sp_5H_3_7_4 | NC_015638.1 | Lacinutrix sp. 5H-3-7-4:Lacinutrix:Flavobacteriaceae:Flavobacteriales:Flavobacteriia:Bacteroidetes:Bacteria |
| Chlorobium_tepidum_TLS | NC_002932.3 | Chlorobium tepidum TLS:Chlorobaculum:Chlorobiaceae:Chlorobiales:Chlorobia:Chlorobi:Bacteria |
| Bradyrhizobium_sp_ORS_278 | NC_009445.1 | Bradyrhizobium sp. ORS 278:Bradyrhizobium:Bradyrhizobiaceae:Rhizobiales:Alphaproteobacteria:Proteobacteria:Bacteria |
| Cellulomonas_fimi_ATCC_484 | NC_015514.1 | Cellulomonas fimi ATCC 484:Cellulomonas:Cellulomonadaceae:Micrococcineae:Actinomycetales:Actinobacteridae:Actinobacteria:Bacteria |
| Corynebacterium_variabile_DSM_44702 | NC_015859.1 | Corynebacterium variabile DSM 44702:Corynebacterium:Corynebacteriaceae:Corynebacterineae:Actinomycetales:Actinobacteridae:Actinobacteria:Bacteria |
| Sebaldella_termitidis_ATCC_33386 | NC_013517.1 | Sebaldella termitidis ATCC 33386:Sebaldella:Leptotrichiaceae:Fusobacteriales:Fusobacteria:Bacteria |
| Burkholderia_glumae_BGR1 | NC_012724.2 | Burkholderia glumae BGR1:Burkholderia:Burkholderiaceae:Burkholderiales:Betaproteobacteria:Proteobacteria:Bacteria |
| Hyphomonas_neptunium_ATCC_15444 | NC_008358.1 | Hyphomonas neptunium ATCC 15444:Hyphomonas:Hyphomonadaceae:Rhodobacterales:Alphaproteobacteria:Proteobacteria:Bacteria |
| Geobacter_sp_M18 | NC_014973.1 | Geobacter sp. M18:Geobacter:Geobacteraceae:Desulfuromonadales:Deltaproteobacteria:Proteobacteria:Bacteria |
| Sphingomonas_wittichii_RW1 | NC_009511.1 | Sphingomonas wittichii RW1:Sphingomonas:Sphingomonadaceae:Sphingomonadales:Alphaproteobacteria:Proteobacteria:Bacteria |
| Francisella_tularensis_subsp_holarctica_FTNF002_00 | NC_009749.1 | Francisella tularensis subsp. holarctica FTNF002-00:Francisella:Francisellaceae:Thiotrichales:Gammaproteobacteria:Proteobacteria:Bacteria |
| Alkaliphilus_oremlandii_OhILAs | NC_009922.1 | Alkaliphilus oremlandii OhILAs:Alkaliphilus:Clostridiaceae:Clostridiales:Clostridia:Firmicutes:Bacteria |
| Lactobacillus_helveticus_DPC_4571 | NC_010080.1 | Lactobacillus helveticus DPC 4571:Lactobacillus:Lactobacillaceae:Lactobacillales:Firmicutes:Bacteria |
| Staphylococcus_carnosus_subsp_carnosus_TM300 | NC_012121.1 | Staphylococcus carnosus subsp. carnosus TM300:Staphylococcus:Bacillales:Firmicutes:Bacteria |
| Sulfurihydrogenibium_sp_YO3AOP1 | NC_010730.1 | Sulfurihydrogenibium sp. YO3AOP1:Sulfurihydrogenibium:Hydrogenothermaceae:Aquificales:Aquificae:Bacteria |
| Shigella_flexneri_2a_str_2457T | NC_004741.1 | Shigella flexneri 2a str. 2457T:Shigella:Enterobacteriaceae:Enterobacteriales:Gammaproteobacteria:Proteobacteria:Bacteria |
| Alkaliphilus_metalliredigens_QYMF | NC_009633.1 | Alkaliphilus metalliredigens QYMF:Alkaliphilus:Clostridiaceae:Clostridiales:Clostridia:Firmicutes:Bacteria |
| Clostridium_thermocellum_DSM_1313 | NC_017304.1 | Clostridium thermocellum DSM 1313:Clostridium:Clostridiaceae:Clostridiales:Clostridia:Firmicutes:Bacteria |
| Burkholderia_multivorans_ATCC_17616 | NC_010805.1 | Burkholderia multivorans ATCC 17616:Burkholderia cepacia complex:Burkholderia:Burkholderiaceae:Burkholderiales:Betaproteobacteria:Proteobacteria:Bacteria |
| Tropheryma_whipplei_str_Twist | NC_004572.3 | Tropheryma whipplei str. Twist:Tropheryma:Micrococcineae:Actinomycetales:Actinobacteridae:Actinobacteria:Bacteria |
| Pectobacterium_atrosepticum_SCRI1043 | NC_004547.2 | Pectobacterium atrosepticum SCRI1043:Pectobacterium:Enterobacteriaceae:Enterobacteriales:Gammaproteobacteria:Proteobacteria:Bacteria |
| Rhodococcus_erythropolis_PR4 | NC_012490.1 | Rhodococcus erythropolis PR4:Rhodococcus:Nocardiaceae:Corynebacterineae:Actinomycetales:Actinobacteridae:Actinobacteria:Bacteria |
| Ruegeria_pomeroyi_DSS_3 | NC_003911.11 | Ruegeria pomeroyi DSS-3:Ruegeria:Rhodobacteraceae:Rhodobacterales:Alphaproteobacteria:Proteobacteria:Bacteria |
| Ralstonia_eutropha_JMP134 | NC_007348.1 | Ralstonia eutropha JMP134:Cupriavidus:Burkholderiaceae:Burkholderiales:Betaproteobacteria:Proteobacteria:Bacteria |
| Rickettsia_canadensis_str_CA410 | NC_016929.1 | Rickettsia canadensis str. CA410:typhus group:Rickettsia:Rickettsieae:Rickettsiaceae:Rickettsiales:Alphaproteobacteria:Proteobacteria:Bacteria |
| Chlorobium_limicola_DSM_245 | NC_010803.1 | Chlorobium limicola DSM 245:Chlorobium:Chlorobium/Pelodictyon group:Chlorobiaceae:Chlorobiales:Chlorobia:Chlorobi:Bacteria |
| Bacillus_pseudofirmus_OF4 | NC_013791.2 | Bacillus pseudofirmus OF4:Bacillus:Bacillaceae:Bacillales:Firmicutes:Bacteria |
| Corynebacterium_ulcerans_809 | NC_017317.1 | Corynebacterium ulcerans 809:Corynebacterium:Corynebacteriaceae:Corynebacterineae:Actinomycetales:Actinobacteridae:Actinobacteria:Bacteria |
| Acidovorax_avenae_subsp_avenae_ATCC_19860 | NC_015138.1 | Acidovorax avenae subsp. avenae ATCC 19860:Acidovorax:Comamonadaceae:Burkholderiales:Betaproteobacteria:Proteobacteria:Bacteria |
| Neorickettsia_risticii_str_Illinois | NC_013009.1 | Neorickettsia risticii str. Illinois:Neorickettsia:Anaplasmataceae:Rickettsiales:Alphaproteobacteria:Proteobacteria:Bacteria |
| Stigmatella_aurantiaca_DW4_3_1 | NC_014623.1 | Stigmatella aurantiaca DW4/3-1:Stigmatella:Cystobacteraceae:Cystobacterineae:Myxococcales:Deltaproteobacteria:Proteobacteria:Bacteria |
| Eubacterium_rectale_ATCC_33656 | NC_012781.1 | Eubacterium rectale ATCC 33656:Eubacterium:Eubacteriaceae:Clostridiales:Clostridia:Firmicutes:Bacteria |
| Burkholderia_pseudomallei_1106a | NC_009078.1 | Burkholderia pseudomallei 1106a:pseudomallei group:Burkholderia:Burkholderiaceae:Burkholderiales:Betaproteobacteria:Proteobacteria:Bacteria |
| Azospirillum_lipoferum_4B | NC_016622.1 | Azospirillum lipoferum 4B:Azospirillum:Rhodospirillaceae:Rhodospirillales:Alphaproteobacteria:Proteobacteria:Bacteria |
| Ethanoligenens_harbinense_YUAN_3 | NC_014828.1 | Ethanoligenens harbinense YUAN-3:Ethanoligenens:Ruminococcaceae:Clostridiales:Clostridia:Firmicutes:Bacteria |
| Rickettsia_typhi_str_B9991CWPP | NC_017062.1 | Rickettsia typhi str. B9991CWPP:typhus group:Rickettsia:Rickettsieae:Rickettsiaceae:Rickettsiales:Alphaproteobacteria:Proteobacteria:Bacteria |
| Hydrogenobaculum_sp_Y04AAS1 | NC_011126.1 | Hydrogenobaculum sp. Y04AAS1:Hydrogenobaculum:Aquificaceae:Aquificales:Aquificae:Bacteria |
| Caldicellulosiruptor_hydrothermalis_108 | NC_014652.1 | Caldicellulosiruptor hydrothermalis 108:Caldicellulosiruptor:Incertae Sedis:Thermoanaerobacterales Family III:Thermoanaerobacterales:Clostridia:Firmicutes:Bacteria |
| Treponema_succinifaciens_DSM_2489 | NC_015385.1 | Treponema succinifaciens DSM 2489:Treponema:Spirochaetaceae:Spirochaetales:Spirochaetes:Bacteria |
| Staphylococcus_aureus_subsp_aureus_11819_97 | NC_017351.1 | Staphylococcus aureus subsp. aureus 11819-97:Staphylococcus:Bacillales:Firmicutes:Bacteria |
| Candidatus_Pelagibacter_sp_IMCC9063 | NC_015380.1 | Candidatus Pelagibacter sp. IMCC9063:Candidatus Pelagibacter:SAR11 cluster:Alphaproteobacteria:Proteobacteria:Bacteria |
| Finegoldia_magna_ATCC_29328 | NC_010376.1 | Finegoldia magna ATCC 29328:Finegoldia:Incertae Sedis:Clostridiales Family XI:Clostridiales:Clostridia:Firmicutes:Bacteria |
| Erysipelothrix_rhusiopathiae | NC_015601.1 | Erysipelothrix rhusiopathiae str. Fujisawa:Erysipelothrix:Erysipelotrichaceae:Erysipelotrichales:Erysipelotrichi:Firmicutes:Bacteria |
| Clostridium_botulinum_A_str_ATCC_19397 | NC_009697.1 | Clostridium botulinum A str. ATCC 19397:Clostridium:Clostridiaceae:Clostridiales:Clostridia:Firmicutes:Bacteria |
| Salinibacter_ruber_M8 | NC_014032.1 | Salinibacter ruber M8:Salinibacter:Rhodothermaceae:Incertae sedis:Bacteroidetes Order II:Bacteroidetes:Bacteria |
| Flavobacterium_johnsoniae_UW101 | NC_009441.1 | Flavobacterium johnsoniae UW101:Flavobacterium:Flavobacteriaceae:Flavobacteriales:Flavobacteriia:Bacteroidetes:Bacteria |
| Thermoanaerobacter_sp_X514 | NC_010320.1 | Thermoanaerobacter sp. X514:Thermoanaerobacter:Thermoanaerobacteraceae:Thermoanaerobacterales:Clostridia:Firmicutes:Bacteria |
| Dechloromonas_aromatica_RCB | NC_007298.1 | Dechloromonas aromatica RCB:Dechloromonas:Rhodocyclaceae:Rhodocyclales:Betaproteobacteria:Proteobacteria:Bacteria |
| Anaeromyxobacter_sp_K | NC_011145.1 | Anaeromyxobacter sp. K:Anaeromyxobacter:Myxococcaceae:Cystobacterineae:Myxococcales:Deltaproteobacteria:Proteobacteria:Bacteria |
| Candidatus_Vesicomyosocius_okutanii_HA | NC_009465.1 | Candidatus Vesicomyosocius okutanii HA:sulfur-oxidizing symbionts:Gammaproteobacteria:Proteobacteria:Bacteria |
| Clostridium_perfringens_SM101 | NC_008265.1 | Clostridium phage phiSM101:unclassified dsDNA phages:dsDNA viruses, no RNA stage:Viruses |
| Thauera_sp_MZ1T | NC_011662.2 | Thauera sp. MZ1T:Thauera:Rhodocyclaceae:Rhodocyclales:Betaproteobacteria:Proteobacteria:Bacteria |
| Candidatus_Solibacter_usitatus_Ellin6076 | NC_008536.1 | Candidatus Solibacter usitatus Ellin6076:Candidatus Solibacter:Solibacteraceae:Solibacterales:Solibacteres:Acidobacteria:Bacteria |
| Haemophilus_parainfluenzae_T3T1 | NC_015964.1 | Haemophilus parainfluenzae T3T1:Haemophilus:Pasteurellaceae:Pasteurellales:Gammaproteobacteria:Proteobacteria:Bacteria |
| Streptosporangium_roseum_DSM_43021 | NC_013595.1 | Streptosporangium roseum DSM 43021:Streptosporangium:Streptosporangiaceae:Streptosporangineae:Actinomycetales:Actinobacteridae:Actinobacteria:Bacteria |
| Rhodopseudomonas_palustris_BisB18 | NC_007925.1 | Rhodopseudomonas palustris BisB18:Rhodopseudomonas:Bradyrhizobiaceae:Rhizobiales:Alphaproteobacteria:Proteobacteria:Bacteria |
| Sphingopyxis_alaskensis_RB2256 | NC_008048.1 | Sphingopyxis alaskensis RB2256:Sphingopyxis:Sphingomonadaceae:Sphingomonadales:Alphaproteobacteria:Proteobacteria:Bacteria |
| Porphyromonas_asaccharolytica_DSM_20707 | NC_015501.1 | Porphyromonas asaccharolytica DSM 20707:Porphyromonas:Porphyromonadaceae:Bacteroidales:Bacteroidia:Bacteroidetes:Bacteria |
| Brucella_melitensis_biovar_Abortus_2308 | NC_007624.1 | Brucella melitensis biovar Abortus 2308:Brucella:Brucellaceae:Rhizobiales:Alphaproteobacteria:Proteobacteria:Bacteria |
| Lactobacillus_plantarum_JDM1 | NC_012984.1 | Lactobacillus plantarum JDM1:Lactobacillus:Lactobacillaceae:Lactobacillales:Firmicutes:Bacteria |
| Leuconostoc_mesenteroides_subsp_mesenteroides_ATCC_8293 | NC_008531.1 | Leuconostoc mesenteroides subsp. mesenteroides ATCC 8293:Leuconostoc:Lactobacillales:Firmicutes:Bacteria |
| Bacillus_amyloliquefaciens_subsp_plantarum_YAU_B9601_Y2 | NC_017061.1 | Bacillus amyloliquefaciens subsp. plantarum YAU B9601-Y2:Bacillus:Bacillaceae:Bacillales:Firmicutes:Bacteria |
| Cyclobacterium_marinum_DSM_745 | NC_015914.1 | Cyclobacterium marinum DSM 745:Cyclobacterium:Cyclobacteriaceae:Cytophagales:Cytophagia:Bacteroidetes:Bacteria |
| Bifidobacterium_breve_ACS_071_V_Sch8b | NC_017218.1 | Bifidobacterium breve ACS-071-V-Sch8b:Bifidobacterium:Bifidobacteriaceae:Bifidobacteriales:Actinobacteridae:Actinobacteria:Bacteria |
| Burkholderia_mallei_ATCC_23344 | NC_006349.2 | Burkholderia mallei ATCC 23344:pseudomallei group:Burkholderia:Burkholderiaceae:Burkholderiales:Betaproteobacteria:Proteobacteria:Bacteria |
| Mycobacterium_tuberculosis_CCDC5180 | NC_017522.1 | Mycobacterium tuberculosis CCDC5180:Mycobacterium tuberculosis complex:Mycobacterium:Mycobacteriaceae:Corynebacterineae:Actinomycetales:Actinobacteridae:Actinobacteria:Bacteria |
| Mobiluncus_curtisii_ATCC_43063 | NC_014246.1 | Mobiluncus curtisii ATCC 43063:Mobiluncus:Mobiluncus/Falcivibrio group:Actinomycetaceae:Actinomycineae:Actinomycetales:Actinobacteridae:Actinobacteria:Bacteria |
| Paenibacillus_polymyxa_E681 | NC_014483.1 | Paenibacillus polymyxa E681:Paenibacillus:Paenibacillaceae:Bacillales:Firmicutes:Bacteria |
| Idiomarina_loihiensis_L2TR | NC_006512.1 | Idiomarina loihiensis L2TR:Idiomarina:Idiomarinaceae:Alteromonadales:Gammaproteobacteria:Proteobacteria:Bacteria |
| Thioalkalivibrio_sulfidophilus_HL_EbGr7 | NC_011901.1 | Thioalkalivibrio sulfidophilus HL-EbGr7:Thioalkalivibrio:Ectothiorhodospiraceae:Chromatiales:Gammaproteobacteria:Proteobacteria:Bacteria |
| Candidatus_Pelagibacter_ubique_HTCC1062 | NC_007205.1 | Candidatus Pelagibacter ubique HTCC1062:Candidatus Pelagibacter:SAR11 cluster:Alphaproteobacteria:Proteobacteria:Bacteria |
| Enterobacter_cloacae_EcWSU1 | NC_016514.1 | Enterobacter cloacae EcWSU1:Enterobacter cloacae complex:Enterobacter:Enterobacteriaceae:Enterobacteriales:Gammaproteobacteria:Proteobacteria:Bacteria |
| Ehrlichia_canis_str_Jake | NC_007354.1 | Ehrlichia canis str. Jake:Ehrlichia:Anaplasmataceae:Rickettsiales:Alphaproteobacteria:Proteobacteria:Bacteria |
| Lactococcus_garvieae_Lg2 | NC_017490.1 | Lactococcus garvieae Lg2:Lactococcus:Streptococcaceae:Lactobacillales:Firmicutes:Bacteria |
| Methylocella_silvestris_BL2 | NC_011666.1 | Methylocella silvestris BL2:Methylocella:Beijerinckiaceae:Rhizobiales:Alphaproteobacteria:Proteobacteria:Bacteria |
| Agrobacterium_tumefaciens_str_C58 | NC_003063.2 | Agrobacterium tumefaciens str. C58:Agrobacterium tumefaciens complex:Agrobacterium:Rhizobium/Agrobacterium group:Rhizobiaceae:Rhizobiales:Alphaproteobacteria:Proteobacteria:Bacteria |
| Haemophilus_somnus_129PT | NC_008309.1 | Haemophilus somnus 129PT:Histophilus:Pasteurellaceae:Pasteurellales:Gammaproteobacteria:Proteobacteria:Bacteria |
| Agrobacterium_radiobacter_K84 | NC_011985.1 | Agrobacterium radiobacter K84:Agrobacterium tumefaciens complex:Agrobacterium:Rhizobium/Agrobacterium group:Rhizobiaceae:Rhizobiales:Alphaproteobacteria:Proteobacteria:Bacteria |
| Streptobacillus_moniliformis_DSM_12112 | NC_013515.1 | Streptobacillus moniliformis DSM 12112:Streptobacillus:Leptotrichiaceae:Fusobacteriales:Fusobacteria:Bacteria |
| Mesorhizobium_opportunistum_WSM2075 | NC_015675.1 | Mesorhizobium opportunistum WSM2075:Mesorhizobium:Phyllobacteriaceae:Rhizobiales:Alphaproteobacteria:Proteobacteria:Bacteria |
| Bacillus_selenitireducens_MLS10 | NC_014219.1 | Bacillus selenitireducens MLS10:Bacillus:Bacillaceae:Bacillales:Firmicutes:Bacteria |
| Streptococcus_mitis_B6 | NC_013853.1 | Streptococcus mitis B6:Streptococcus:Streptococcaceae:Lactobacillales:Firmicutes:Bacteria |
| Burkholderia_cenocepacia_HI2424 | NC_008544.1 | Burkholderia cenocepacia HI2424:Burkholderia cepacia complex:Burkholderia:Burkholderiaceae:Burkholderiales:Betaproteobacteria:Proteobacteria:Bacteria |
| Rhizobium_leguminosarum_bv_trifolii_WSM2304 | NC_011369.1 | Rhizobium leguminosarum bv. trifolii WSM2304:Rhizobium:Rhizobium/Agrobacterium group:Rhizobiaceae:Rhizobiales:Alphaproteobacteria:Proteobacteria:Bacteria |
| Bacteroides_fragilis_NCTC_9343 | NC_003228.3 | Bacteroides fragilis NCTC 9343:Bacteroides:Bacteroidaceae:Bacteroidales:Bacteroidia:Bacteroidetes:Bacteria |
| Brucella_abortus_A13334 | NC_016795.1 | Brucella abortus A13334:Brucella:Brucellaceae:Rhizobiales:Alphaproteobacteria:Proteobacteria:Bacteria |
| Listeria_welshimeri_serovar_6b_str_SLCC5334 | NC_008555.1 | Listeria welshimeri serovar 6b str. SLCC5334:Listeria:Listeriaceae:Bacillales:Firmicutes:Bacteria |
| Desulfococcus_oleovorans_Hxd3 | NC_009943.1 | Desulfococcus oleovorans Hxd3:Desulfococcus:Desulfobacteraceae:Desulfobacterales:Deltaproteobacteria:Proteobacteria:Bacteria |
| Nitrosomonas_eutropha_C91 | NC_008344.1 | Nitrosomonas eutropha C91:Nitrosomonas:Nitrosomonadaceae:Nitrosomonadales:Betaproteobacteria:Proteobacteria:Bacteria |
| Candidatus_Blochmannia_pennsylvanicus_str_BPEN | NC_007292.1 | Candidatus Blochmannia pennsylvanicus str. BPEN:Candidatus Blochmannia:ant endosymbionts:Enterobacteriaceae:Enterobacteriales:Gammaproteobacteria:Proteobacteria:Bacteria |
| Rickettsia_philipii_str_364D | NC_016930.1 | Rickettsia philipii str. 364D:spotted fever group:Rickettsia:Rickettsieae:Rickettsiaceae:Rickettsiales:Alphaproteobacteria:Proteobacteria:Bacteria |
| Bifidobacterium_animalis_subsp_lactis_AD011 | NC_011835.1 | Bifidobacterium animalis subsp. lactis AD011:Bifidobacterium:Bifidobacteriaceae:Bifidobacteriales:Actinobacteridae:Actinobacteria:Bacteria |
| Denitrovibrio_acetiphilus_DSM_12809 | NC_013943.1 | Denitrovibrio acetiphilus DSM 12809:Denitrovibrio:Deferribacteraceae:Deferribacterales:Deferribacteres:Bacteria |
| Chloroherpeton_thalassium_ATCC_35110 | NC_011026.1 | Chloroherpeton thalassium ATCC 35110:Chloroherpeton:Chlorobiaceae:Chlorobiales:Chlorobia:Chlorobi:Bacteria |
| Candidatus_Koribacter_versatilis_Ellin345 | NC_008009.1 | Candidatus Koribacter versatilis Ellin345:Candidatus Koribacter:Acidobacteria:Bacteria |
| Ralstonia_pickettii_12J | NC_010682.1 | Ralstonia pickettii 12J:Ralstonia:Burkholderiaceae:Burkholderiales:Betaproteobacteria:Proteobacteria:Bacteria |
| Shewanella_piezotolerans_WP3 | NC_011566.1 | Shewanella piezotolerans WP3:Shewanella:Shewanellaceae:Alteromonadales:Gammaproteobacteria:Proteobacteria:Bacteria |
| Nitrosomonas_sp_AL212 | NC_015222.1 | Nitrosomonas sp. AL212:Nitrosomonas:Nitrosomonadaceae:Nitrosomonadales:Betaproteobacteria:Proteobacteria:Bacteria |
| Geobacillus_sp_WCH70 | NC_012793.1 | Geobacillus sp. WCH70:Geobacillus:Bacillaceae:Bacillales:Firmicutes:Bacteria |
| Helicobacter_pylori | NC_017354.1 | Helicobacter pylori 52:Helicobacter:Helicobacteraceae:Campylobacterales:Epsilonproteobacteria:Proteobacteria:Bacteria |
| Chlorobium_chlorochromatii_CaD3 | NC_007514.1 | Chlorobium chlorochromatii CaD3:Chlorobium:Chlorobium/Pelodictyon group:Chlorobiaceae:Chlorobiales:Chlorobia:Chlorobi:Bacteria |
| Ilyobacter_polytropus_DSM_2926 | NC_014632.1 | Ilyobacter polytropus DSM 2926:Ilyobacter:Fusobacteriaceae:Fusobacteriales:Fusobacteria:Bacteria |
| Clostridium_phytofermentans_ISDg | NC_010001.1 | Clostridium phytofermentans ISDg:Clostridium:Clostridiaceae:Clostridiales:Clostridia:Firmicutes:Bacteria |
| Desulfosporosinus_orientis_DSM_765 | NC_016584.1 | Desulfosporosinus orientis DSM 765:Desulfosporosinus:Peptococcaceae:Clostridiales:Clostridia:Firmicutes:Bacteria |
| Xylella_fastidiosa_9a5c | NC_002488.3 | Xylella fastidiosa 9a5c:Xylella:Xanthomonadaceae:Xanthomonadales:Gammaproteobacteria:Proteobacteria:Bacteria |
| Ketogulonigenium_vulgarum_WSH_001 | NC_017384.1 | Ketogulonigenium vulgarum WSH-001:Ketogulonicigenium:Rhodobacteraceae:Rhodobacterales:Alphaproteobacteria:Proteobacteria:Bacteria |
| Brucella_canis_HSK_A52141 | NC_016796.1 | Brucella canis HSK A52141:Brucella:Brucellaceae:Rhizobiales:Alphaproteobacteria:Proteobacteria:Bacteria |
| Phycisphaera_mikurensis_NBRC_102666 | NC_017080.1 | Phycisphaera mikurensis NBRC 102666:Phycisphaera:Phycisphaeraceae:Phycisphaerales:Phycisphaerae:Planctomycetes:Bacteria |
| Streptococcus_thermophilus_CNRZ1066 | NC_006449.1 | Streptococcus thermophilus CNRZ1066:Streptococcus:Streptococcaceae:Lactobacillales:Firmicutes:Bacteria |
| Lactobacillus_helveticus_H10 | NC_017467.1 | Lactobacillus helveticus H10:Lactobacillus:Lactobacillaceae:Lactobacillales:Firmicutes:Bacteria |
| Legionella_pneumophila_2300_99_Alcoy | NC_014125.1 | Legionella pneumophila 2300/99 Alcoy:Legionella:Legionellaceae:Legionellales:Gammaproteobacteria:Proteobacteria:Bacteria |
| Candidatus_Nitrospira_defluvii | NC_014355.1 | Candidatus Nitrospira defluvii:Nitrospira:Nitrospiraceae:Nitrospirales:Nitrospirae:Bacteria |
| Candidatus_Hodgkinia_cicadicola_Dsem | NC_012960.1 | Candidatus Hodgkinia cicadicola Dsem:Candidatus Hodgkinia:Rhizobiales:Alphaproteobacteria:Proteobacteria:Bacteria |
| Gluconobacter_oxydans_621H | NC_006677.1 | Gluconobacter oxydans 621H:Gluconobacter:Acetobacteraceae:Rhodospirillales:Alphaproteobacteria:Proteobacteria:Bacteria |
| Rhodopseudomonas_palustris_BisA53 | NC_008435.1 | Rhodopseudomonas palustris BisA53:Rhodopseudomonas:Bradyrhizobiaceae:Rhizobiales:Alphaproteobacteria:Proteobacteria:Bacteria |
| Hirschia_baltica_ATCC_49814 | NC_012982.1 | Hirschia baltica ATCC 49814:Hirschia:Hyphomonadaceae:Rhodobacterales:Alphaproteobacteria:Proteobacteria:Bacteria |
| Dickeya_dadantii_3937 | NC_014500.1 | Dickeya dadantii 3937:Dickeya:Enterobacteriaceae:Enterobacteriales:Gammaproteobacteria:Proteobacteria:Bacteria |
| Desulfitobacterium_hafniense_DCB_2 | NC_011830.1 | Desulfitobacterium hafniense DCB-2:Desulfitobacterium:Peptococcaceae:Clostridiales:Clostridia:Firmicutes:Bacteria |
| Acetobacterium_woodii_DSM_1030 | NC_016894.1 | Acetobacterium woodii DSM 1030:Acetobacterium:Eubacteriaceae:Clostridiales:Clostridia:Firmicutes:Bacteria |
| Streptomyces_cattleya_NRRL_8057 | NC_017586.1 | Streptomyces cattleya NRRL 8057 = DSM 46488:Streptomyces:Streptomycetaceae:Streptomycineae:Actinomycetales:Actinobacteridae:Actinobacteria:Bacteria |
| Roseobacter_litoralis_Och_149 | NC_015730.1 | Roseobacter litoralis Och 149:Roseobacter:Rhodobacteraceae:Rhodobacterales:Alphaproteobacteria:Proteobacteria:Bacteria |
| Chloroflexus_aggregans_DSM_9485 | NC_011831.1 | Chloroflexus aggregans DSM 9485:Chloroflexus:Chloroflexaceae:Chloroflexales:Chloroflexi:Bacteria |
| Pedobacter_heparinus_DSM_2366 | NC_013061.1 | Pedobacter heparinus DSM 2366:Pedobacter:Sphingobacteriaceae:Sphingobacteriales:Sphingobacteriia:Bacteroidetes:Bacteria |
| Polynucleobacter_necessarius_subsp_asymbioticus_QLW_P1DMWA_1 | NC_009379.1 | Polynucleobacter necessarius subsp. asymbioticus QLW-P1DMWA-1:Polynucleobacter:Burkholderiaceae:Burkholderiales:Betaproteobacteria:Proteobacteria:Bacteria |
| Brachyspira_intermedia_PWS_A | NC_017243.1 | Brachyspira intermedia PWS/A:Brachyspira:Brachyspiraceae:Spirochaetales:Spirochaetes:Bacteria |
| Corynebacterium_jeikeium_K411 | NC_007164.1 | Corynebacterium jeikeium K411:Corynebacterium:Corynebacteriaceae:Corynebacterineae:Actinomycetales:Actinobacteridae:Actinobacteria:Bacteria |
| Lactobacillus_sakei_subsp_sakei_23K | NC_007576.1 | Lactobacillus sakei subsp. sakei 23K:Lactobacillus:Lactobacillaceae:Lactobacillales:Firmicutes:Bacteria |
| Ammonifex_degensii_KC4 | NC_013385.1 | Ammonifex degensii KC4:Ammonifex:Moorella group:Thermoanaerobacteraceae:Thermoanaerobacterales:Clostridia:Firmicutes:Bacteria |
| Corynebacterium_aurimucosum_ATCC_700975 | NC_012590.1 | Corynebacterium aurimucosum ATCC 700975:Corynebacterium:Corynebacteriaceae:Corynebacterineae:Actinomycetales:Actinobacteridae:Actinobacteria:Bacteria |
| Bordetella_petrii_DSM_12804 | NC_010170.1 | Bordetella petrii DSM 12804:Bordetella:Alcaligenaceae:Burkholderiales:Betaproteobacteria:Proteobacteria:Bacteria |
| Pasteurella_multocida_36950 | NC_016808.1 | Pasteurella multocida 36950:Pasteurella:Pasteurellaceae:Pasteurellales:Gammaproteobacteria:Proteobacteria:Bacteria |
| Olsenella_uli_DSM_7084 | NC_014363.1 | Olsenella uli DSM 7084:Olsenella:Coriobacteriaceae:Coriobacterineae:Coriobacteriales:Coriobacteridae:Actinobacteria:Bacteria |
| Shewanella_frigidimarina_NCIMB_400 | NC_008345.1 | Shewanella frigidimarina NCIMB 400:Shewanella:Shewanellaceae:Alteromonadales:Gammaproteobacteria:Proteobacteria:Bacteria |
| Lactococcus_lactis_subsp_cremoris_A76 | NC_017492.1 | Lactococcus lactis subsp. cremoris A76:Lactococcus:Streptococcaceae:Lactobacillales:Firmicutes:Bacteria |
| Oceanimonas_sp_GK1 | NC_016745.1 | Oceanimonas sp. GK1:Oceanimonas:Aeromonadaceae:Aeromonadales:Gammaproteobacteria:Proteobacteria:Bacteria |
| Candidatus_Phytoplasma_mali | NC_011047.1 | Candidatus Phytoplasma mali:16SrX (Apple proliferation group):Candidatus Phytoplasma:Acholeplasmataceae:Acholeplasmatales:Mollicutes:Tenericutes:Bacteria |
| Listeria_seeligeri_serovar_1_2b_str_SLCC3954 | NC_013891.1 | Listeria seeligeri serovar 1/2b str. SLCC3954:Listeria:Listeriaceae:Bacillales:Firmicutes:Bacteria |
| Bifidobacterium_longum_subsp_infantis_157F | NC_015052.1 | Bifidobacterium longum subsp. infantis 157F:Bifidobacterium:Bifidobacteriaceae:Bifidobacteriales:Actinobacteridae:Actinobacteria:Bacteria |
| Erwinia_tasmaniensis_Et1_99 | NC_010694.1 | Erwinia tasmaniensis Et1/99:Erwinia:Enterobacteriaceae:Enterobacteriales:Gammaproteobacteria:Proteobacteria:Bacteria |
| Leuconostoc_citreum_KM20 | NC_010471.1 | Leuconostoc citreum KM20:Leuconostoc:Lactobacillales:Firmicutes:Bacteria |
| Burkholderia_rhizoxinica_HKI_454 | NC_014722.1 | Burkholderia rhizoxinica HKI 454:Burkholderia:Burkholderiaceae:Burkholderiales:Betaproteobacteria:Proteobacteria:Bacteria |
| Bifidobacterium_longum_DJO10A | NC_010816.1 | Bifidobacterium longum DJO10A:Bifidobacterium:Bifidobacteriaceae:Bifidobacteriales:Actinobacteridae:Actinobacteria:Bacteria |
| Shewanella_halifaxensis_HAW_EB4 | NC_010334.1 | Shewanella halifaxensis HAW-EB4:Shewanella:Shewanellaceae:Alteromonadales:Gammaproteobacteria:Proteobacteria:Bacteria |
| Yersinia_enterocolitica_subsp_palearctica_105.5Rr | NC_015224.1 | Yersinia enterocolitica subsp. palearctica 105.5R(r):Yersinia:Enterobacteriaceae:Enterobacteriales:Gammaproteobacteria:Proteobacteria:Bacteria |
| Bartonella_clarridgeiae_73 | NC_014932.1 | Bartonella clarridgeiae 73:Bartonella:Bartonellaceae:Rhizobiales:Alphaproteobacteria:Proteobacteria:Bacteria |
| Desulfovibrio_vulgaris_DP4 | NC_008751.1 | Desulfovibrio vulgaris DP4:Desulfovibrio:Desulfovibrionaceae:Desulfovibrionales:Deltaproteobacteria:Proteobacteria:Bacteria |
| Azospirillum_sp_B510 | NC_013854.1 | Azospirillum sp. B510:Azospirillum:Rhodospirillaceae:Rhodospirillales:Alphaproteobacteria:Proteobacteria:Bacteria |
| Lactobacillus_johnsonii_DPC_6026 | NC_017477.1 | Lactobacillus johnsonii DPC 6026:Lactobacillus:Lactobacillaceae:Lactobacillales:Firmicutes:Bacteria |
| Desulfovibrio_desulfuricans_ND132 | NC_016803.1 | Desulfovibrio desulfuricans ND132:Desulfovibrio:Desulfovibrionaceae:Desulfovibrionales:Deltaproteobacteria:Proteobacteria:Bacteria |
| Lactobacillus_rhamnosus_ATCC_8530 | NC_017491.1 | Lactobacillus rhamnosus ATCC 8530:Lactobacillus:Lactobacillaceae:Lactobacillales:Firmicutes:Bacteria |
| Thermoanaerobacterium_xylanolyticum_LX_11 | NC_015555.1 | Thermoanaerobacterium xylanolyticum LX-11:Thermoanaerobacterium:Incertae Sedis:Thermoanaerobacterales Family III:Thermoanaerobacterales:Clostridia:Firmicutes:Bacteria |
| Gordonia_bronchialis_DSM_43247 | NC_013441.1 | Gordonia bronchialis DSM 43247:Gordonia:Gordoniaceae:Corynebacterineae:Actinomycetales:Actinobacteridae:Actinobacteria:Bacteria |
| Burkholderia_xenovorans_LB400 | NC_007953.1 | Burkholderia xenovorans LB400:Burkholderia:Burkholderiaceae:Burkholderiales:Betaproteobacteria:Proteobacteria:Bacteria |
| Thermoanaerobacter_tengcongensis_MB4 | NC_003869.1 | Thermoanaerobacter tengcongensis MB4:Caldanaerobacter:Thermoanaerobacteraceae:Thermoanaerobacterales:Clostridia:Firmicutes:Bacteria |
| Anaplasma_marginale_str_Florida | NC_012026.1 | Anaplasma marginale str. Florida:Anaplasma:Anaplasmataceae:Rickettsiales:Alphaproteobacteria:Proteobacteria:Bacteria |
| Cupriavidus_taiwanensis_LMG_19424 | NC_010530.1 | Cupriavidus taiwanensis LMG 19424:Cupriavidus:Burkholderiaceae:Burkholderiales:Betaproteobacteria:Proteobacteria:Bacteria |
| Rhodospirillum_centenum_SW | NC_011420.2 | Rhodospirillum centenum SW:Rhodospirillum:Rhodospirillaceae:Rhodospirillales:Alphaproteobacteria:Proteobacteria:Bacteria |
| Gallionella_capsiferriformans_ES_2 | NC_014394.1 | Gallionella capsiferriformans ES-2:Gallionella:Gallionellaceae:Gallionellales:Betaproteobacteria:Proteobacteria:Bacteria |
| Vibrio_harveyi_ATCC_BAA_1116 | NC_009784.1 | Vibrio harveyi ATCC BAA-1116:Vibrio:Vibrionaceae:Vibrionales:Gammaproteobacteria:Proteobacteria:Bacteria |
| Corynebacterium_resistens_DSM_45100 | NC_015673.1 | Corynebacterium resistens DSM 45100:Corynebacterium:Corynebacteriaceae:Corynebacterineae:Actinomycetales:Actinobacteridae:Actinobacteria:Bacteria |
| Leuconostoc_sp_C2 | NC_015734.1 | Leuconostoc sp. C2:Leuconostoc:Lactobacillales:Firmicutes:Bacteria |
| Buchnera_aphidicola_str_Ak_Acyrthosiphon_kondoi | NC_017256.1 | Buchnera aphidicola str. Ak (Acyrthosiphon kondoi):Buchnera:Enterobacteriaceae:Enterobacteriales:Gammaproteobacteria:Proteobacteria:Bacteria |
| Anabaena_variabilis_ATCC_29413 | NC_014000.1 | Anabaena variabilis ATCC 29413:Anabaena:Nostocaceae:Nostocales:Cyanobacteria:Bacteria |
| Acidovorax_ebreus_TPSY | NC_011992.1 | Acidovorax ebreus TPSY:Acidovorax:Comamonadaceae:Burkholderiales:Betaproteobacteria:Proteobacteria:Bacteria |
| Kytococcus_sedentarius_DSM_20547 | NC_013169.1 | Kytococcus sedentarius DSM 20547:Kytococcus:Dermacoccaceae:Micrococcineae:Actinomycetales:Actinobacteridae:Actinobacteria:Bacteria |
| Caldisericum_exile_AZM16c01 | NC_017096.1 | Caldisericum exile AZM16c01:Caldisericum:Caldisericaceae:Caldisericales:Caldisericia:Caldiserica:Bacteria |
| Runella_slithyformis_DSM_19594 | NC_015703.1 | Runella slithyformis DSM 19594:Runella:Cytophagaceae:Cytophagales:Cytophagia:Bacteroidetes:Bacteria |
| Erwinia_sp_Ejp617 | NC_017445.1 | Erwinia sp. Ejp617:Erwinia:Enterobacteriaceae:Enterobacteriales:Gammaproteobacteria:Proteobacteria:Bacteria |
| Streptococcus_dysgalactiae_subsp_equisimilis_ATCC_12394 | NC_017567.1 | Streptococcus dysgalactiae subsp. equisimilis ATCC 12394:Streptococcus:Streptococcaceae:Lactobacillales:Firmicutes:Bacteria |
| Campylobacter_concisus_13826 | NC_009802.1 | Campylobacter concisus 13826:Campylobacter:Campylobacteraceae:Campylobacterales:Epsilonproteobacteria:Proteobacteria:Bacteria |
| Flexistipes_sinusarabici_DSM_4947 | NC_015672.1 | Flexistipes sinusarabici DSM 4947:Flexistipes:Deferribacteraceae:Deferribacterales:Deferribacteres:Bacteria |
| Eubacterium_eligens_ATCC_27750 | NC_012778.1 | Eubacterium eligens ATCC 27750:Eubacterium:Eubacteriaceae:Clostridiales:Clostridia:Firmicutes:Bacteria |
| Streptococcus_pasteurianus_ATCC_43144 | NC_015600.1 | Streptococcus pasteurianus ATCC 43144:Streptococcus:Streptococcaceae:Lactobacillales:Firmicutes:Bacteria |
| Herminiimonas_arsenicoxydans | NC_009138.1 | Herminiimonas arsenicoxydans:Herminiimonas:Oxalobacteraceae:Burkholderiales:Betaproteobacteria:Proteobacteria:Bacteria |
| Methylococcus_capsulatus_str_Bath | NC_002977.6 | Methylococcus capsulatus str. Bath:Methylococcus:Methylococcaceae:Methylococcales:Gammaproteobacteria:Proteobacteria:Bacteria |
| Ehrlichia_ruminantium_str_Gardel | NC_006831.1 | Ehrlichia ruminantium str. Gardel:Ehrlichia:Anaplasmataceae:Rickettsiales:Alphaproteobacteria:Proteobacteria:Bacteria |
| Shewanella_sp_MR_4 | NC_008321.1 | Shewanella sp. MR-4:Shewanella:Shewanellaceae:Alteromonadales:Gammaproteobacteria:Proteobacteria:Bacteria |
| Gemmatimonas_aurantiaca_T_27 | NC_012489.1 | Gemmatimonas aurantiaca T-27:Gemmatimonas:Gemmatimonadaceae:Gemmatimonadales:Gemmatimonadetes:Bacteria |
| Streptomyces_avermitilis_MA_4680 | NC_003155.4 | Streptomyces avermitilis MA-4680:Streptomyces:Streptomycetaceae:Streptomycineae:Actinomycetales:Actinobacteridae:Actinobacteria:Bacteria |
| Janthinobacterium_sp_Marseille | NC_009659.1 | Janthinobacterium sp. Marseille:Janthinobacterium:Oxalobacteraceae:Burkholderiales:Betaproteobacteria:Proteobacteria:Bacteria |
| Acidithiobacillus_ferrivorans_SS3 | NC_015942.1 | Acidithiobacillus ferrivorans SS3:Acidithiobacillus:Acidithiobacillaceae:Acidithiobacillales:Gammaproteobacteria:Proteobacteria:Bacteria |
| Burkholderia_phytofirmans_PsJN | NC_010681.1 | Burkholderia phytofirmans PsJN:Burkholderia:Burkholderiaceae:Burkholderiales:Betaproteobacteria:Proteobacteria:Bacteria |
| Thermoanaerobacter_pseudethanolicus_ATCC_33223 | NC_010321.1 | Thermoanaerobacter pseudethanolicus ATCC 33223:Thermoanaerobacter:Thermoanaerobacteraceae:Thermoanaerobacterales:Clostridia:Firmicutes:Bacteria |
| Pseudoxanthomonas_spadix_BD_a59 | NC_016147.1 | Pseudoxanthomonas spadix BD-a59:Pseudoxanthomonas:Xanthomonadaceae:Xanthomonadales:Gammaproteobacteria:Proteobacteria:Bacteria |
| Rahnella_aquatilis_CIP_78.65 | NC_016818.1 | Rahnella aquatilis CIP 78.65 = ATCC 33071:Rahnella:Enterobacteriaceae:Enterobacteriales:Gammaproteobacteria:Proteobacteria:Bacteria |
| Roseiflexus_castenholzii_DSM_13941 | NC_009767.1 | Roseiflexus castenholzii DSM 13941:Roseiflexus:Chloroflexaceae:Chloroflexales:Chloroflexi:Bacteria |
| Streptococcus_thermophilus_JIM_8232 | NC_017581.1 | Streptococcus thermophilus JIM 8232:Streptococcus:Streptococcaceae:Lactobacillales:Firmicutes:Bacteria |
| Carnobacterium_sp_17_4 | NC_015391.1 | Carnobacterium sp. 17-4:Carnobacterium:Carnobacteriaceae:Lactobacillales:Firmicutes:Bacteria |
| Francisella_tularensis_subsp_holarctica_LVS | NC_007880.1 | Francisella tularensis subsp. holarctica LVS:Francisella:Francisellaceae:Thiotrichales:Gammaproteobacteria:Proteobacteria:Bacteria |
| Aquifex_aeolicus_VF5 | NC_000918.1 | Aquifex aeolicus VF5:Aquifex:Aquificaceae:Aquificales:Aquificae:Bacteria |
| Buchnera_aphidicola_BCc | NC_008513.1 | Buchnera aphidicola BCc:Buchnera:Enterobacteriaceae:Enterobacteriales:Gammaproteobacteria:Proteobacteria:Bacteria |
| Ehrlichia_ruminantium_str_Welgevonden | NC_006832.1 | Ehrlichia ruminantium str. Welgevonden:Ehrlichia:Anaplasmataceae:Rickettsiales:Alphaproteobacteria:Proteobacteria:Bacteria |
| Hahella_chejuensis_KCTC_2396 | NC_007645.1 | Hahella chejuensis KCTC 2396:Hahella:Hahellaceae:Oceanospirillales:Gammaproteobacteria:Proteobacteria:Bacteria |
| Halanaerobium_hydrogeniformans | NC_014654.1 | Halanaerobium hydrogeniformans:Halanaerobium:Halanaerobiaceae:Halanaerobiales:Clostridia:Firmicutes:Bacteria |
| Xenorhabdus_nematophila_ATCC_19061 | NC_014228.1 | Xenorhabdus nematophila ATCC 19061:Xenorhabdus:Enterobacteriaceae:Enterobacteriales:Gammaproteobacteria:Proteobacteria:Bacteria |
| Azorhizobium_caulinodans_ORS_571 | NC_009937.1 | Azorhizobium caulinodans ORS 571:Azorhizobium:Xanthobacteraceae:Rhizobiales:Alphaproteobacteria:Proteobacteria:Bacteria |
| Helicobacter_felis_ATCC_49179 | NC_014810.2 | Helicobacter felis ATCC 49179:Helicobacter:Helicobacteraceae:Campylobacterales:Epsilonproteobacteria:Proteobacteria:Bacteria |
| Bradyrhizobium_japonicum_USDA_6 | NC_017249.1 | Bradyrhizobium japonicum USDA 6:Bradyrhizobium:Bradyrhizobiaceae:Rhizobiales:Alphaproteobacteria:Proteobacteria:Bacteria |
| Leptothrix_cholodnii_SP_6 | NC_010524.1 | Leptothrix cholodnii SP-6:Leptothrix:Burkholderiales:Betaproteobacteria:Proteobacteria:Bacteria |
| Methylobacterium_nodulans_ORS_2060 | NC_011894.1 | Methylobacterium nodulans ORS 2060:Methylobacterium:Methylobacteriaceae:Rhizobiales:Alphaproteobacteria:Proteobacteria:Bacteria |
| Shewanella_putrefaciens_200 | NC_017566.1 | Shewanella putrefaciens 200:Shewanella:Shewanellaceae:Alteromonadales:Gammaproteobacteria:Proteobacteria:Bacteria |
| Macrococcus_caseolyticus_JCSC5402 | NC_011999.1 | Macrococcus caseolyticus JCSC5402:Macrococcus:Bacillales:Firmicutes:Bacteria |
| Oligotropha_carboxidovorans_OM4 | NC_017538.1 | Oligotropha carboxidovorans OM4:Oligotropha:Bradyrhizobiaceae:Rhizobiales:Alphaproteobacteria:Proteobacteria:Bacteria |
| Pseudomonas_brassicacearum_subsp_brassicacearum_NFM421 | NC_015379.1 | Pseudomonas brassicacearum subsp. brassicacearum NFM421:Pseudomonas:Pseudomonadaceae:Pseudomonadales:Gammaproteobacteria:Proteobacteria:Bacteria |
| Cellvibrio_gilvus_ATCC_13127 | NC_015671.1 | [Cellvibrio] gilvus ATCC 13127:Cellulomonas:Cellulomonadaceae:Micrococcineae:Actinomycetales:Actinobacteridae:Actinobacteria:Bacteria |
| Sinorhizobium_meliloti_1021 | NC_003047.1 | Sinorhizobium meliloti 1021:Sinorhizobium:Sinorhizobium/Ensifer group:Rhizobiaceae:Rhizobiales:Alphaproteobacteria:Proteobacteria:Bacteria |
| Shigella_boydii_Sb227 | NC_007613.1 | Shigella boydii Sb227:Shigella:Enterobacteriaceae:Enterobacteriales:Gammaproteobacteria:Proteobacteria:Bacteria |
| Alicyclobacillus_acidocaldarius_subsp_acidocaldarius_DSM_446 | NC_013205.1 | Alicyclobacillus acidocaldarius subsp. acidocaldarius DSM 446:Alicyclobacillus:Alicyclobacillaceae:Bacillales:Firmicutes:Bacteria |
| Nocardiopsis_dassonvillei_subsp_dassonvillei_DSM_43111 | NC_014210.1 | Nocardiopsis dassonvillei subsp. dassonvillei DSM 43111:Nocardiopsis:Nocardiopsaceae:Streptosporangineae:Actinomycetales:Actinobacteridae:Actinobacteria:Bacteria |
| Rickettsia_parkeri_str_Portsmouth | NC_017044.1 | Rickettsia parkeri str. Portsmouth:spotted fever group:Rickettsia:Rickettsieae:Rickettsiaceae:Rickettsiales:Alphaproteobacteria:Proteobacteria:Bacteria |
| Rickettsia_canadensis_str_McKiel | NC_009879.1 | Rickettsia canadensis str. McKiel:typhus group:Rickettsia:Rickettsieae:Rickettsiaceae:Rickettsiales:Alphaproteobacteria:Proteobacteria:Bacteria |
| Methylibium_petroleiphilum_PM1 | NC_008825.1 | Methylibium petroleiphilum PM1:Methylibium:Burkholderiales:Betaproteobacteria:Proteobacteria:Bacteria |
| Methylomicrobium_alcaliphilum | NC_016112.1 | Methylomicrobium alcaliphilum:Methylomicrobium:Methylococcaceae:Methylococcales:Gammaproteobacteria:Proteobacteria:Bacteria |
| Lawsonia_intracellularis_PHE_MN1_00 | NC_008011.1 | Lawsonia intracellularis PHE/MN1-00:Lawsonia:Desulfovibrionaceae:Desulfovibrionales:Deltaproteobacteria:Proteobacteria:Bacteria |
| Listeria_monocytogenes_07PF0776 | NC_017728.1 | Listeria monocytogenes 07PF0776:Listeria:Listeriaceae:Bacillales:Firmicutes:Bacteria |
| Anaplasma_centrale_str_Israel | NC_013532.1 | Anaplasma centrale str. Israel:Anaplasma:Anaplasmataceae:Rickettsiales:Alphaproteobacteria:Proteobacteria:Bacteria |
| Halorhodospira_halophila_SL1 | NC_008789.1 | Halorhodospira halophila SL1:Halorhodospira:Ectothiorhodospiraceae:Chromatiales:Gammaproteobacteria:Proteobacteria:Bacteria |
| Streptomyces_violaceusniger_Tu_4113 | NC_015957.1 | Streptomyces violaceusniger Tu 4113:Streptomyces:Streptomycetaceae:Streptomycineae:Actinomycetales:Actinobacteridae:Actinobacteria:Bacteria |
| Moorella_thermoacetica_ATCC_39073 | NC_007644.1 | Moorella thermoacetica ATCC 39073:Moorella:Moorella group:Thermoanaerobacteraceae:Thermoanaerobacterales:Clostridia:Firmicutes:Bacteria |
| Candidatus_Ruthia_magnifica_str_Cm_Calyptogena_magnifica | NC_008610.1 | Candidatus Ruthia magnifica str. Cm (Calyptogena magnifica):sulfur-oxidizing symbionts:Gammaproteobacteria:Proteobacteria:Bacteria |
| Arthrobacter_arilaitensis_Re117 | NC_014550.1 | Arthrobacter arilaitensis Re117:Arthrobacter:Micrococcaceae:Micrococcineae:Actinomycetales:Actinobacteridae:Actinobacteria:Bacteria |
| Sanguibacter_keddieii_DSM_10542 | NC_013521.1 | Sanguibacter keddieii DSM 10542:Sanguibacter:Sanguibacteraceae:Micrococcineae:Actinomycetales:Actinobacteridae:Actinobacteria:Bacteria |
| Glaciecola_sp_4H_3_7+YE_5 | NC_015497.1 | Glaciecola sp. 4H-3-7+YE-5:Glaciecola:Alteromonadaceae:Alteromonadales:Gammaproteobacteria:Proteobacteria:Bacteria |
| Pusillimonas_sp_T7_7 | NC_015458.1 | Pusillimonas sp. T7-7:Pusillimonas:Alcaligenaceae:Burkholderiales:Betaproteobacteria:Proteobacteria:Bacteria |
| Salmonella_enterica_subsp_enterica_serovar_Agona_str_SL483 | NC_011149.1 | Salmonella enterica subsp. enterica serovar Agona str. SL483:enterica serovar Agona:Salmonella enterica subsp:Salmonella:Enterobacteriaceae:Enterobacteriales:Gammaproteobacteria:Proteobacteria:Bacteria |
| Bacteroides_thetaiotaomicron_VPI_5482 | NC_004663.1 | Bacteroides thetaiotaomicron VPI-5482:Bacteroides:Bacteroidaceae:Bacteroidales:Bacteroidia:Bacteroidetes:Bacteria |
| Clostridium_cellulovorans_743B | NC_014393.1 | Clostridium cellulovorans 743B:Clostridium:Clostridiaceae:Clostridiales:Clostridia:Firmicutes:Bacteria |
| Mesoplasma_florum_L1 | NC_006055.1 | Mesoplasma florum L1:Mesoplasma:Entomoplasmataceae:Entomoplasmatales:Mollicutes:Tenericutes:Bacteria |
| Prochlorococcus_marinus_str_NATL2A | NC_007335.2 | Prochlorococcus marinus str. NATL2A:Prochlorococcus:Prochlorococcaceae:Prochlorales:Cyanobacteria:Bacteria |
| Rhodobacter_capsulatus_SB_1003 | NC_014034.1 | Rhodobacter capsulatus SB 1003:Rhodobacter:Rhodobacteraceae:Rhodobacterales:Alphaproteobacteria:Proteobacteria:Bacteria |
| Clostridium_acetobutylicum_ATCC_824 | NC_003030.1 | Clostridium acetobutylicum ATCC 824:Clostridium:Clostridiaceae:Clostridiales:Clostridia:Firmicutes:Bacteria |
| Azoarcus_sp_BH72 | NC_008702.1 | Azoarcus sp. BH72:Azoarcus:Rhodocyclaceae:Rhodocyclales:Betaproteobacteria:Proteobacteria:Bacteria |
| Candidatus_Amoebophilus_asiaticus_5a2 | NC_010830.1 | Candidatus Amoebophilus asiaticus 5a2:Candidatus Amoebophilus:Bacteroidetes:Bacteria |
| Thermoanaerobacter_sp_X513 | NC_014538.1 | Thermoanaerobacter sp. X513:Thermoanaerobacter:Thermoanaerobacteraceae:Thermoanaerobacterales:Clostridia:Firmicutes:Bacteria |
| Cupriavidus_necator_N_1 | NC_015726.1 | Cupriavidus necator N-1:Cupriavidus:Burkholderiaceae:Burkholderiales:Betaproteobacteria:Proteobacteria:Bacteria |
| Pseudomonas_syringae_pv_phaseolicola_1448A | NC_005773.3 | Pseudomonas syringae pv. phaseolicola 1448A:Pseudomonas savastanoi:Pseudomonas:Pseudomonadaceae:Pseudomonadales:Gammaproteobacteria:Proteobacteria:Bacteria |
| Isosphaera_pallida_ATCC_43644 | NC_014962.1 | Isosphaera pallida ATCC 43644:Isosphaera:Planctomycetaceae:Planctomycetales:Planctomycetia:Planctomycetes:Bacteria |
| Pseudomonas_stutzeri_ATCC_17588 | NC_015740.1 | Pseudomonas stutzeri ATCC 17588 = LMG 11199:Pseudomonas:Pseudomonadaceae:Pseudomonadales:Gammaproteobacteria:Proteobacteria:Bacteria |
| Bifidobacterium_adolescentis_ATCC_15703 | NC_008618.1 | Bifidobacterium adolescentis ATCC 15703:Bifidobacterium:Bifidobacteriaceae:Bifidobacteriales:Actinobacteridae:Actinobacteria:Bacteria |
| Xanthomonas_oryzae_pv_oryzae_KACC_10331 | NC_006834.1 | Xanthomonas oryzae pv. oryzae KACC 10331:Xanthomonas:Xanthomonadaceae:Xanthomonadales:Gammaproteobacteria:Proteobacteria:Bacteria |
| Ureaplasma_parvum_serovar_3_str_ATCC_27815 | NC_010503.1 | Ureaplasma parvum serovar 3 str. ATCC 27815:Ureaplasma:Mycoplasmataceae:Mollicutes:Tenericutes:Bacteria |
| Shewanella_sediminis_HAW_EB3 | NC_009831.1 | Shewanella sediminis HAW-EB3:Shewanella:Shewanellaceae:Alteromonadales:Gammaproteobacteria:Proteobacteria:Bacteria |
| Xanthomonas_oryzae_pv_oryzae_MAFF_311018 | NC_007705.1 | Xanthomonas oryzae pv. oryzae MAFF 311018:Xanthomonas:Xanthomonadaceae:Xanthomonadales:Gammaproteobacteria:Proteobacteria:Bacteria |
| Anaeromyxobacter_sp_Fw109_5 | NC_009675.1 | Anaeromyxobacter sp. Fw109-5:Anaeromyxobacter:Myxococcaceae:Cystobacterineae:Myxococcales:Deltaproteobacteria:Proteobacteria:Bacteria |
| Streptococcus_parasanguinis_ATCC_15912 | NC_015678.1 | Streptococcus parasanguinis ATCC 15912:Streptococcus:Streptococcaceae:Lactobacillales:Firmicutes:Bacteria |
| Candidatus_Arthromitus_sp_SFB_mouse_Japan | NC_015913.1 | Candidatus Arthromitus sp. SFB-mouse-Japan:Candidatus Arthromitus:Clostridiaceae:Clostridiales:Clostridia:Firmicutes:Bacteria |
| Streptomyces_griseus_subsp_griseus_NBRC_13350 | NC_010572.1 | Streptomyces griseus subsp. griseus NBRC 13350:Streptomyces:Streptomycetaceae:Streptomycineae:Actinomycetales:Actinobacteridae:Actinobacteria:Bacteria |
| Prevotella_melaninogenica_ATCC_25845 | NC_014371.1 | Prevotella melaninogenica ATCC 25845:Prevotella:Prevotellaceae:Bacteroidales:Bacteroidia:Bacteroidetes:Bacteria |
| Caulobacter_crescentus_NA1000 | NC_011916.1 | Caulobacter crescentus NA1000:Caulobacter:Caulobacteraceae:Caulobacterales:Alphaproteobacteria:Proteobacteria:Bacteria |
| Hippea_maritima_DSM_10411 | NC_015318.1 | Hippea maritima DSM 10411:Hippea:Desulfurellaceae:Desulfurellales:Deltaproteobacteria:Proteobacteria:Bacteria |
| Shewanella_baltica_OS117 | NC_017579.1 | Shewanella baltica OS117:Shewanella:Shewanellaceae:Alteromonadales:Gammaproteobacteria:Proteobacteria:Bacteria |
| Sulfurimonas_autotrophica_DSM_16294 | NC_014506.1 | Sulfurimonas autotrophica DSM 16294:Sulfurimonas:Helicobacteraceae:Campylobacterales:Epsilonproteobacteria:Proteobacteria:Bacteria |
| Heliobacterium_modesticaldum_Ice1 | NC_010337.2 | Heliobacterium modesticaldum Ice1:Heliobacterium:Heliobacteriaceae:Clostridiales:Clostridia:Firmicutes:Bacteria |
| Corynebacterium_diphtheriae_31A | NC_016799.1 | Corynebacterium diphtheriae 31A:Corynebacterium:Corynebacteriaceae:Corynebacterineae:Actinomycetales:Actinobacteridae:Actinobacteria:Bacteria |
| Thiomicrospira_crunogena_XCL_2 | NC_007520.2 | Thiomicrospira crunogena XCL-2:Thiomicrospira:Piscirickettsiaceae:Thiotrichales:Gammaproteobacteria:Proteobacteria:Bacteria |
| Burkholderia_ambifaria_AMMD | NC_008392.1 | Burkholderia ambifaria AMMD:Burkholderia cepacia complex:Burkholderia:Burkholderiaceae:Burkholderiales:Betaproteobacteria:Proteobacteria:Bacteria |
| Candidatus_Phytoplasma_australiense | NC_010544.1 | Candidatus Phytoplasma australiense:16SrXII (Stolbur group):Candidatus Phytoplasma:Acholeplasmataceae:Acholeplasmatales:Mollicutes:Tenericutes:Bacteria |
| Zobellia_galactanivorans | NC_015844.1 | Zobellia galactanivorans:Zobellia:Flavobacteriaceae:Flavobacteriales:Flavobacteriia:Bacteroidetes:Bacteria |
| Yersinia_enterocolitica_subsp_enterocolitica_8081 | NC_008800.1 | Yersinia enterocolitica subsp. enterocolitica 8081:Yersinia:Enterobacteriaceae:Enterobacteriales:Gammaproteobacteria:Proteobacteria:Bacteria |
| Arcobacter_sp_L | NC_017192.1 | Arcobacter sp. L:Arcobacter:Campylobacteraceae:Campylobacterales:Epsilonproteobacteria:Proteobacteria:Bacteria |
| Brevibacillus_brevis_NBRC_100599 | NC_012491.1 | Brevibacillus brevis NBRC 100599:Brevibacillus:Paenibacillaceae:Bacillales:Firmicutes:Bacteria |
| Thermoanaerobacter_mathranii_subsp_mathranii_str_A3 | NC_014209.1 | Thermoanaerobacter mathranii subsp. mathranii str. A3:Thermoanaerobacter:Thermoanaerobacteraceae:Thermoanaerobacterales:Clostridia:Firmicutes:Bacteria |
| Aeromonas_salmonicida_subsp_salmonicida_A449 | NC_009348.1 | Aeromonas salmonicida subsp. salmonicida A449:Aeromonas:Aeromonadaceae:Aeromonadales:Gammaproteobacteria:Proteobacteria:Bacteria |
| Brucella_microti_CCM_4915 | NC_013119.1 | Brucella microti CCM 4915:Brucella:Brucellaceae:Rhizobiales:Alphaproteobacteria:Proteobacteria:Bacteria |
| Frateuria_aurantia_DSM_6220 | NC_017033.1 | Frateuria aurantia DSM 6220:Frateuria:Xanthomonadaceae:Xanthomonadales:Gammaproteobacteria:Proteobacteria:Bacteria |
| Shewanella_sp_ANA_3 | NC_008577.1 | Shewanella sp. ANA-3:Shewanella:Shewanellaceae:Alteromonadales:Gammaproteobacteria:Proteobacteria:Bacteria |
| Vibrio_sp_Ex25 | NC_013457.1 | Vibrio sp. Ex25:Vibrio:Vibrionaceae:Vibrionales:Gammaproteobacteria:Proteobacteria:Bacteria |
| Neisseria_meningitidis_8013 | NC_017501.1 | Neisseria meningitidis 8013:Neisseria:Neisseriaceae:Neisseriales:Betaproteobacteria:Proteobacteria:Bacteria |
| Candidatus_Midichloria_mitochondrii_IricVA | NC_015722.1 | Candidatus Midichloria mitochondrii IricVA:Candidatus Midichloria:Rickettsiales:Alphaproteobacteria:Proteobacteria:Bacteria |
| Mycobacterium_abscessus_ATCC_19977 | NC_010397.1 | Mycobacterium abscessus ATCC 19977:Mycobacterium abscessus:Mycobacterium:Mycobacteriaceae:Corynebacterineae:Actinomycetales:Actinobacteridae:Actinobacteria:Bacteria |
| Dinoroseobacter_shibae_DFL_12 | NC_009952.1 | Dinoroseobacter shibae DFL 12:Dinoroseobacter:Rhodobacteraceae:Rhodobacterales:Alphaproteobacteria:Proteobacteria:Bacteria |
| Photorhabdus_asymbiotica_subsp_asymbiotica_ATCC_43949 | NC_012962.1 | Photorhabdus asymbiotica subsp. asymbiotica ATCC 43949:Photorhabdus:Enterobacteriaceae:Enterobacteriales:Gammaproteobacteria:Proteobacteria:Bacteria |
| Rothia_dentocariosa_ATCC_17931 | NC_014643.1 | Rothia dentocariosa ATCC 17931:Rothia:Micrococcaceae:Micrococcineae:Actinomycetales:Actinobacteridae:Actinobacteria:Bacteria |
| Acidimicrobium_ferrooxidans_DSM_10331 | NC_013124.1 | Acidimicrobium ferrooxidans DSM 10331:Acidimicrobium:Acidimicrobiaceae:Acidimicrobineae:Acidimicrobiales:Acidimicrobidae:Actinobacteria:Bacteria |
| Dehalogenimonas_lykanthroporepellens_BL_DC_9 | NC_014314.1 | Dehalogenimonas lykanthroporepellens BL-DC-9:Dehalogenimonas:Dehalococcoidetes:Chloroflexi:Bacteria |
| Zymomonas_mobilis_subsp_mobilis_NCIMB_11163 | NC_013355.1 | Zymomonas mobilis subsp. mobilis NCIMB 11163:Zymomonas:Sphingomonadaceae:Sphingomonadales:Alphaproteobacteria:Proteobacteria:Bacteria |
| Corallococcus_coralloides_DSM_2259 | NC_017030.1 | Corallococcus coralloides DSM 2259:Corallococcus:Myxococcaceae:Cystobacterineae:Myxococcales:Deltaproteobacteria:Proteobacteria:Bacteria |
| Thermus_thermophilus_SG0.5JP17_16 | NC_017272.1 | Thermus thermophilus SG0.5JP17-16:Thermus:Thermaceae:Thermales:Deinococci:Deinococcus-Thermus:Bacteria |
| Micrococcus_luteus_NCTC_2665 | NC_012803.1 | Micrococcus luteus NCTC 2665:Micrococcus:Micrococcaceae:Micrococcineae:Actinomycetales:Actinobacteridae:Actinobacteria:Bacteria |
| Mycobacterium_tuberculosis_CCDC5079 | NC_017523.1 | Mycobacterium tuberculosis CCDC5079:Mycobacterium tuberculosis complex:Mycobacterium:Mycobacteriaceae:Corynebacterineae:Actinomycetales:Actinobacteridae:Actinobacteria:Bacteria |
| Ralstonia_solanacearum_GMI1000 | NC_003295.1 | Ralstonia solanacearum GMI1000:Ralstonia:Burkholderiaceae:Burkholderiales:Betaproteobacteria:Proteobacteria:Bacteria |
| Rickettsia_prowazekii_str_BuV67_CWPP | NC_017056.1 | Rickettsia prowazekii str. BuV67-CWPP:typhus group:Rickettsia:Rickettsieae:Rickettsiaceae:Rickettsiales:Alphaproteobacteria:Proteobacteria:Bacteria |
| Streptomyces_sp_SirexAA_E | NC_015953.1 | Streptomyces sp. SirexAA-E:Streptomyces:Streptomycetaceae:Streptomycineae:Actinomycetales:Actinobacteridae:Actinobacteria:Bacteria |
| Bacillus_coagulans_2_6 | NC_015634.1 | Bacillus coagulans 2-6:Bacillus:Bacillaceae:Bacillales:Firmicutes:Bacteria |
| Vibrio_anguillarum_775 | NC_015637.1 | Vibrio anguillarum 775:Listonella:Vibrionaceae:Vibrionales:Gammaproteobacteria:Proteobacteria:Bacteria |
| Bifidobacterium_animalis_subsp_lactis_BB_12 | NC_017214.1 | Bifidobacterium animalis subsp. lactis BB-12:Bifidobacterium:Bifidobacteriaceae:Bifidobacteriales:Actinobacteridae:Actinobacteria:Bacteria |
| Streptomyces_bingchenggensis_BCW_1 | NC_016582.1 | Streptomyces bingchenggensis BCW-1:Streptomyces:Streptomycetaceae:Streptomycineae:Actinomycetales:Actinobacteridae:Actinobacteria:Bacteria |
| Frankia_alni_ACN14a | NC_008278.1 | Frankia alni ACN14a:Frankia:Frankiaceae:Frankineae:Actinomycetales:Actinobacteridae:Actinobacteria:Bacteria |
| Marinomonas_posidonica_IVIA_Po_181 | NC_015559.1 | Marinomonas posidonica IVIA-Po-181:Marinomonas:Oceanospirillales:Gammaproteobacteria:Proteobacteria:Bacteria |
| Rubrivivax_gelatinosus_IL144 | NC_017075.1 | Rubrivivax gelatinosus IL144:Rubrivivax:Burkholderiales:Betaproteobacteria:Proteobacteria:Bacteria |
| Sulfobacillus_acidophilus_TPY | NC_015757.1 | Sulfobacillus acidophilus TPY:Sulfobacillus:Incertae Sedis:Clostridiales Family XVII:Clostridiales:Clostridia:Firmicutes:Bacteria |
| Brucella_pinnipedialis_B2_94 | NC_015858.1 | Brucella pinnipedialis B2/94:Brucella:Brucellaceae:Rhizobiales:Alphaproteobacteria:Proteobacteria:Bacteria |
| Deferribacter_desulfuricans_SSM1 | NC_013939.1 | Deferribacter desulfuricans SSM1:Deferribacter:Deferribacteraceae:Deferribacterales:Deferribacteres:Bacteria |
| Neisseria_gonorrhoeae_FA_1090 | NC_002946.2 | Neisseria gonorrhoeae FA 1090:Neisseria:Neisseriaceae:Neisseriales:Betaproteobacteria:Proteobacteria:Bacteria |
| Ralstonia_pickettii_12D | NC_012857.1 | Ralstonia pickettii 12D:Ralstonia:Burkholderiaceae:Burkholderiales:Betaproteobacteria:Proteobacteria:Bacteria |
| Streptococcus_mutans_NN2025 | NC_013928.1 | Streptococcus mutans NN2025:Streptococcus:Streptococcaceae:Lactobacillales:Firmicutes:Bacteria |
| Streptococcus_uberis_0140J | NC_012004.1 | Streptococcus uberis 0140J:Streptococcus:Streptococcaceae:Lactobacillales:Firmicutes:Bacteria |
| Enterobacter_aerogenes_KCTC_2190 | NC_015663.1 | Enterobacter aerogenes KCTC 2190:Enterobacter:Enterobacteriaceae:Enterobacteriales:Gammaproteobacteria:Proteobacteria:Bacteria |
| Geobacillus_thermodenitrificans_NG80_2 | NC_009328.1 | Geobacillus thermodenitrificans NG80-2:Geobacillus:Bacillaceae:Bacillales:Firmicutes:Bacteria |
| Riemerella_anatipestifer_ATCC_11845 | NC_017045.1 | Riemerella anatipestifer ATCC 11845 = DSM 15868:Riemerella:Flavobacteriaceae:Flavobacteriales:Flavobacteriia:Bacteroidetes:Bacteria |
| Burkholderia_thailandensis_E264 | NC_007651.1 | Burkholderia thailandensis E264:pseudomallei group:Burkholderia:Burkholderiaceae:Burkholderiales:Betaproteobacteria:Proteobacteria:Bacteria |
| Brevundimonas_subvibrioides_ATCC_15264 | NC_014375.1 | Brevundimonas subvibrioides ATCC 15264:Brevundimonas:Caulobacteraceae:Caulobacterales:Alphaproteobacteria:Proteobacteria:Bacteria |
| Agrobacterium_vitis_S4 | NC_011989.1 | Agrobacterium vitis S4:Agrobacterium:Rhizobium/Agrobacterium group:Rhizobiaceae:Rhizobiales:Alphaproteobacteria:Proteobacteria:Bacteria |
| Burkholderia_multivorans_ATCC_17616 | NC_010087.1 | Burkholderia multivorans ATCC 17616:Burkholderia cepacia complex:Burkholderia:Burkholderiaceae:Burkholderiales:Betaproteobacteria:Proteobacteria:Bacteria |
| Xanthomonas_axonopodis_pv_citrumelo_F1 | NC_016010.1 | Xanthomonas axonopodis pv. citrumelo F1:Xanthomonas:Xanthomonadaceae:Xanthomonadales:Gammaproteobacteria:Proteobacteria:Bacteria |
| Leadbetterella_byssophila_DSM_17132 | NC_014655.1 | Leadbetterella byssophila DSM 17132:Leadbetterella:Cytophagaceae:Cytophagales:Cytophagia:Bacteroidetes:Bacteria |
| Kitasatospora_setae_KM_6054 | NC_016109.1 | Kitasatospora setae KM-6054:Kitasatospora:Streptomycetaceae:Streptomycineae:Actinomycetales:Actinobacteridae:Actinobacteria:Bacteria |
| Edwardsiella_tarda_EIB202 | NC_013508.1 | Edwardsiella tarda EIB202:Edwardsiella:Enterobacteriaceae:Enterobacteriales:Gammaproteobacteria:Proteobacteria:Bacteria |
| Intrasporangium_calvum_DSM_43043 | NC_014830.1 | Intrasporangium calvum DSM 43043:Intrasporangium:Intrasporangiaceae:Micrococcineae:Actinomycetales:Actinobacteridae:Actinobacteria:Bacteria |
| Onion_yellows_phytoplasma_OY_M | NC_005303.2 | Onion yellows phytoplasma OY-M:Candidatus Phytoplasma asteris:Candidatus Phytoplasma:Acholeplasmataceae:Acholeplasmatales:Mollicutes:Tenericutes:Bacteria |
| Streptococcus_oralis_Uo5 | NC_015291.1 | Streptococcus oralis Uo5:Streptococcus:Streptococcaceae:Lactobacillales:Firmicutes:Bacteria |
| Flavobacterium_columnare_ATCC_49512 | NC_016510.1 | Flavobacterium columnare ATCC 49512:Flavobacterium:Flavobacteriaceae:Flavobacteriales:Flavobacteriia:Bacteroidetes:Bacteria |
| Pseudomonas_aeruginosa_LESB58 | NC_011770.1 | Pseudomonas aeruginosa LESB58:Pseudomonas:Pseudomonadaceae:Pseudomonadales:Gammaproteobacteria:Proteobacteria:Bacteria |
| Halobacillus_halophilus_DSM_2266 | NC_017668.1 | Halobacillus halophilus DSM 2266:Halobacillus:Bacillaceae:Bacillales:Firmicutes:Bacteria |
| Selenomonas_sputigena_ATCC_35185 | NC_015437.1 | Selenomonas sputigena ATCC 35185:Selenomonas:Veillonellaceae:Selenomonadales:Negativicutes:Firmicutes:Bacteria |
| Chlorobaculum_parvum_NCIB_8327 | NC_011027.1 | Chlorobaculum parvum NCIB 8327:Chlorobaculum:Chlorobiaceae:Chlorobiales:Chlorobia:Chlorobi:Bacteria |
| Pantoea_ananatis_AJ13355 | NC_017531.1 | Pantoea ananatis AJ13355:Pantoea:Enterobacteriaceae:Enterobacteriales:Gammaproteobacteria:Proteobacteria:Bacteria |
| Serratia_plymuthica_AS9 | NC_015567.1 | Serratia plymuthica AS9:Serratia:Enterobacteriaceae:Enterobacteriales:Gammaproteobacteria:Proteobacteria:Bacteria |
| Streptococcus_infantarius_subsp_infantarius_CJ18 | NC_016826.1 | Streptococcus infantarius subsp. infantarius CJ18:Streptococcus:Streptococcaceae:Lactobacillales:Firmicutes:Bacteria |
| Amycolicicoccus_subflavus_DQS3_9A1 | NC_015564.1 | Amycolicicoccus subflavus DQS3-9A1:Amycolicicoccus:Mycobacteriaceae:Corynebacterineae:Actinomycetales:Actinobacteridae:Actinobacteria:Bacteria |
| Clostridium_cellulolyticum_H10 | NC_011898.1 | Clostridium cellulolyticum H10:Clostridium:Clostridiaceae:Clostridiales:Clostridia:Firmicutes:Bacteria |
| Aromatoleum_aromaticum_EbN1 | NC_006513.1 | Aromatoleum aromaticum EbN1:Aromatoleum:Rhodocyclaceae:Rhodocyclales:Betaproteobacteria:Proteobacteria:Bacteria |
| Chlamydia_trachomatis_434_Bu | NC_010287.1 | Chlamydia trachomatis 434/Bu:Chlamydia:Chlamydia/Chlamydophila group:Chlamydiaceae:Chlamydiales:Chlamydiae:Bacteria |
| Acinetobacter_calcoaceticus_PHEA_2 | NC_016603.1 | Acinetobacter calcoaceticus PHEA-2:Acinetobacter calcoaceticus/baumannii complex:Acinetobacter:Moraxellaceae:Pseudomonadales:Gammaproteobacteria:Proteobacteria:Bacteria |
| Shigella_sonnei_Ss046 | NC_007384.1 | Shigella sonnei Ss046:Shigella:Enterobacteriaceae:Enterobacteriales:Gammaproteobacteria:Proteobacteria:Bacteria |
| Nitrobacter_hamburgensis_X14 | NC_007964.1 | Nitrobacter hamburgensis X14:Nitrobacter:Bradyrhizobiaceae:Rhizobiales:Alphaproteobacteria:Proteobacteria:Bacteria |
| Acidithiobacillus_ferrooxidans_ATCC_53993 | NC_011206.1 | Acidithiobacillus ferrooxidans ATCC 53993:Acidithiobacillus:Acidithiobacillaceae:Acidithiobacillales:Gammaproteobacteria:Proteobacteria:Bacteria |
| Clostridium_clariflavum_DSM_19732 | NC_016627.1 | Clostridium clariflavum DSM 19732:Clostridium:Clostridiaceae:Clostridiales:Clostridia:Firmicutes:Bacteria |
| Enterobacter_asburiae_LF7a | NC_015968.1 | Enterobacter asburiae LF7a:Enterobacter cloacae complex:Enterobacter:Enterobacteriaceae:Enterobacteriales:Gammaproteobacteria:Proteobacteria:Bacteria |
| Streptococcus_agalactiae_2603V_R | NC_004116.1 | Streptococcus agalactiae 2603V/R:Streptococcus:Streptococcaceae:Lactobacillales:Firmicutes:Bacteria |
| Bacillus_coagulans_36D1 | NC_016023.1 | Bacillus coagulans 36D1:Bacillus:Bacillaceae:Bacillales:Firmicutes:Bacteria |
| Bacillus_amyloliquefaciens | NC_016784.1 | Bacillus amyloliquefaciens subsp. plantarum CAU B946:Bacillus:Bacillaceae:Bacillales:Firmicutes:Bacteria |
| Sphingobacterium_sp_21 | NC_015277.1 | Sphingobacterium sp. 21:Sphingobacterium:Sphingobacteriaceae:Sphingobacteriales:Sphingobacteriia:Bacteroidetes:Bacteria |
| Pantoea_sp_At_9b | NC_014837.1 | Pantoea sp. At-9b:Pantoea:Enterobacteriaceae:Enterobacteriales:Gammaproteobacteria:Proteobacteria:Bacteria |
| Sinorhizobium_meliloti_AK83 | NC_015596.1 | Sinorhizobium meliloti AK83:Sinorhizobium:Sinorhizobium/Ensifer group:Rhizobiaceae:Rhizobiales:Alphaproteobacteria:Proteobacteria:Bacteria |
| Bacillus_pumilus_SAFR_032 | NC_009848.1 | Bacillus pumilus SAFR-032:Bacillus:Bacillaceae:Bacillales:Firmicutes:Bacteria |
| Streptomyces_cattleya_NRRL_8057 | NC_016111.1 | Streptomyces cattleya NRRL 8057 = DSM 46488:Streptomyces:Streptomycetaceae:Streptomycineae:Actinomycetales:Actinobacteridae:Actinobacteria:Bacteria |
| Aerococcus_urinae_ACS_120_V_Col10a | NC_015278.1 | Aerococcus urinae ACS-120-V-Col10a:Aerococcus:Aerococcaceae:Lactobacillales:Firmicutes:Bacteria |
| Thermaerobacter_marianensis_DSM_12885 | NC_014831.1 | Thermaerobacter marianensis DSM 12885:Thermaerobacter:Incertae Sedis:Clostridiales Family XVII:Clostridiales:Clostridia:Firmicutes:Bacteria |
| Dyadobacter_fermentans_DSM_18053 | NC_013037.1 | Dyadobacter fermentans DSM 18053:Dyadobacter:Cytophagaceae:Cytophagales:Cytophagia:Bacteroidetes:Bacteria |
| Stenotrophomonas_maltophilia_D457 | NC_017671.1 | Stenotrophomonas maltophilia D457:Stenotrophomonas maltophilia group:Stenotrophomonas:Xanthomonadaceae:Xanthomonadales:Gammaproteobacteria:Proteobacteria:Bacteria |
| Tepidanaerobacter_sp_Re1 | NC_015519.1 | Tepidanaerobacter sp. Re1:Tepidanaerobacter:Thermoanaerobacteraceae:Thermoanaerobacterales:Clostridia:Firmicutes:Bacteria |
| Krokinobacter_sp_4H_3_7_5 | NC_015496.1 | Krokinobacter sp. 4H-3-7-5:Krokinobacter:Flavobacteriaceae:Flavobacteriales:Flavobacteriia:Bacteroidetes:Bacteria |
| Frankia_sp_EuI1c | NC_014666.1 | Frankia sp. EuI1c:Frankia:Frankiaceae:Frankineae:Actinomycetales:Actinobacteridae:Actinobacteria:Bacteria |
| Clostridium_difficile_630 | NC_009089.1 | Clostridium difficile 630:Peptostreptococcaceae:Clostridiales:Clostridia:Firmicutes:Bacteria |
| Neisseria_lactamica_020_06 | NC_014752.1 | Neisseria lactamica 020-06:Neisseria:Neisseriaceae:Neisseriales:Betaproteobacteria:Proteobacteria:Bacteria |
| Brachybacterium_faecium_DSM_4810 | NC_013172.1 | Brachybacterium faecium DSM 4810:Brachybacterium:Dermabacteraceae:Micrococcineae:Actinomycetales:Actinobacteridae:Actinobacteria:Bacteria |
| Bacteroides_vulgatus_ATCC_8482 | NC_009614.1 | Bacteroides vulgatus ATCC 8482:Bacteroides:Bacteroidaceae:Bacteroidales:Bacteroidia:Bacteroidetes:Bacteria |
| Citrobacter_rodentium_ICC168 | NC_013716.1 | Citrobacter rodentium ICC168:Citrobacter:Enterobacteriaceae:Enterobacteriales:Gammaproteobacteria:Proteobacteria:Bacteria |
| Pasteurella_multocida_subsp_multocida_str_HN06 | NC_017027.1 | Pasteurella multocida subsp. multocida str. HN06:Pasteurella:Pasteurellaceae:Pasteurellales:Gammaproteobacteria:Proteobacteria:Bacteria |
| Teredinibacter_turnerae_T7901 | NC_012997.1 | Teredinibacter turnerae T7901:Teredinibacter:Alteromonadales genera incertae sedis:Alteromonadales:Gammaproteobacteria:Proteobacteria:Bacteria |
| Thermovibrio_ammonificans_HB_1 | NC_014926.1 | Thermovibrio ammonificans HB-1:Thermovibrio:Desulfurobacteriaceae:Aquificales:Aquificae:Bacteria |
| Bifidobacterium_bifidum_S17 | NC_014616.1 | Bifidobacterium bifidum S17:Bifidobacterium:Bifidobacteriaceae:Bifidobacteriales:Actinobacteridae:Actinobacteria:Bacteria |
| Dictyoglomus_turgidum_DSM_6724 | NC_011661.1 | Dictyoglomus turgidum DSM 6724:Dictyoglomus:Dictyoglomaceae:Dictyoglomales:Dictyoglomi:Bacteria |
| Cytophaga_hutchinsonii_ATCC_33406 | NC_008255.1 | Cytophaga hutchinsonii ATCC 33406:Cytophaga:Cytophagaceae:Cytophagales:Cytophagia:Bacteroidetes:Bacteria |
| Klebsiella_pneumoniae_KCTC_2242 | NC_017540.1 | Klebsiella pneumoniae KCTC 2242:Klebsiella:Enterobacteriaceae:Enterobacteriales:Gammaproteobacteria:Proteobacteria:Bacteria |
| Nitrosospira_multiformis_ATCC_25196 | NC_007614.1 | Nitrosospira multiformis ATCC 25196:Nitrosospira:Nitrosomonadaceae:Nitrosomonadales:Betaproteobacteria:Proteobacteria:Bacteria |
| Acidiphilium_cryptum_JF_5 | NC_009484.1 | Acidiphilium cryptum JF-5:Acidiphilium:Acetobacteraceae:Rhodospirillales:Alphaproteobacteria:Proteobacteria:Bacteria |
| Desulfotomaculum_acetoxidans_DSM_771 | NC_013216.1 | Desulfotomaculum acetoxidans DSM 771:Desulfotomaculum:Peptococcaceae:Clostridiales:Clostridia:Firmicutes:Bacteria |
| Paenibacillus_terrae_HPL_003 | NC_016641.1 | Paenibacillus terrae HPL-003:Paenibacillus:Paenibacillaceae:Bacillales:Firmicutes:Bacteria |
| Pseudonocardia_dioxanivorans_CB1190 | NC_015312.1 | Pseudonocardia dioxanivorans CB1190:Pseudonocardia:Pseudonocardiaceae:Pseudonocardineae:Actinomycetales:Actinobacteridae:Actinobacteria:Bacteria |
| Spirochaeta_africana_DSM_8902 | NC_017098.1 | Spirochaeta africana DSM 8902:Spirochaeta:Spirochaetaceae:Spirochaetales:Spirochaetes:Bacteria |
| Pantoea_ananatis_LMG_20103 | NC_013956.1 | Pantoea ananatis LMG 20103:Pantoea:Enterobacteriaceae:Enterobacteriales:Gammaproteobacteria:Proteobacteria:Bacteria |
| Candidatus_Blochmannia_floridanus | NC_005061.1 | Candidatus Blochmannia floridanus:Candidatus Blochmannia:ant endosymbionts:Enterobacteriaceae:Enterobacteriales:Gammaproteobacteria:Proteobacteria:Bacteria |
| Exiguobacterium_sibiricum_255_15 | NC_010556.1 | Exiguobacterium sibiricum 255-15:Exiguobacterium:Incertae Sedis:Bacillales Family XII:Bacillales:Firmicutes:Bacteria |
| Xanthomonas_campestris_pv_campestris_str_8004 | NC_007086.1 | Xanthomonas campestris pv. campestris str. 8004:Xanthomonas:Xanthomonadaceae:Xanthomonadales:Gammaproteobacteria:Proteobacteria:Bacteria |
| Hydrogenobaculum_sp_3684 | NC_015557.1 | Hydrogenobaculum sp. 3684:Hydrogenobaculum:Aquificaceae:Aquificales:Aquificae:Bacteria |
| Campylobacter_jejuni_subsp_doylei_269.97 | NC_009707.1 | Campylobacter jejuni subsp. doylei 269.97:Campylobacter:Campylobacteraceae:Campylobacterales:Epsilonproteobacteria:Proteobacteria:Bacteria |
| Thermodesulfatator_indicus_DSM_15286 | NC_015681.1 | Thermodesulfatator indicus DSM 15286:Thermodesulfatator:Thermodesulfobacteriaceae:Thermodesulfobacteriales:Thermodesulfobacteria:Bacteria |
| Arthrobacter_sp_FB24 | NC_008541.1 | Arthrobacter sp. FB24:Arthrobacter:Micrococcaceae:Micrococcineae:Actinomycetales:Actinobacteridae:Actinobacteria:Bacteria |
| Helicobacter_acinonychis_str_Sheeba | NC_008229.1 | Helicobacter acinonychis str. Sheeba:Helicobacter:Helicobacteraceae:Campylobacterales:Epsilonproteobacteria:Proteobacteria:Bacteria |
| Chloroflexus_aurantiacus_J_10_fl | NC_010175.1 | Chloroflexus aurantiacus J-10-fl:Chloroflexus:Chloroflexaceae:Chloroflexales:Chloroflexi:Bacteria |
| Xylanimonas_cellulosilytica_DSM_15894 | NC_013530.1 | Xylanimonas cellulosilytica DSM 15894:Xylanimonas:Promicromonosporaceae:Micrococcineae:Actinomycetales:Actinobacteridae:Actinobacteria:Bacteria |
| Enterococcus_faecalis_62 | NC_017732.1 | Enterococcus phage EF62phi:unclassified phages:Viruses |
| Thermoanaerobacter_wiegelii_Rt8.B1 | NC_015958.1 | Thermoanaerobacter wiegelii Rt8.B1:Thermoanaerobacter:Thermoanaerobacteraceae:Thermoanaerobacterales:Clostridia:Firmicutes:Bacteria |
| Acidithiobacillus_caldus_SM_1 | NC_015850.1 | Acidithiobacillus caldus SM-1:Acidithiobacillus:Acidithiobacillaceae:Acidithiobacillales:Gammaproteobacteria:Proteobacteria:Bacteria |
| Lactococcus_garvieae_ATCC_49156 | NC_015930.1 | Lactococcus garvieae ATCC 49156:Lactococcus:Streptococcaceae:Lactobacillales:Firmicutes:Bacteria |
| Shigella_flexneri_2002017 | NC_017328.1 | Shigella flexneri 2002017:Shigella:Enterobacteriaceae:Enterobacteriales:Gammaproteobacteria:Proteobacteria:Bacteria |
| Pseudomonas_mendocina_ymp | NC_009439.1 | Pseudomonas mendocina ymp:Pseudomonas:Pseudomonadaceae:Pseudomonadales:Gammaproteobacteria:Proteobacteria:Bacteria |
| Candidatus_Carsonella_ruddii_PV | NC_008512.1 | Candidatus Carsonella ruddii PV:Candidatus Carsonella:Gammaproteobacteria:Proteobacteria:Bacteria |
| Thioalkalimicrobium_cyclicum_ALM1 | NC_015581.1 | Thioalkalimicrobium cyclicum ALM1:Thioalkalimicrobium:Piscirickettsiaceae:Thiotrichales:Gammaproteobacteria:Proteobacteria:Bacteria |
| Corynebacterium_pseudotuberculosis_1002 | NC_017300.1 | Corynebacterium pseudotuberculosis 1002:Corynebacterium:Corynebacteriaceae:Corynebacterineae:Actinomycetales:Actinobacteridae:Actinobacteria:Bacteria |
| Thioalkalivibrio_sp_K90mix | NC_013889.1 | Thioalkalivibrio sp. K90mix:Thioalkalivibrio:Ectothiorhodospiraceae:Chromatiales:Gammaproteobacteria:Proteobacteria:Bacteria |
| Shewanella_loihica_PV_4 | NC_009092.1 | Shewanella loihica PV-4:Shewanella:Shewanellaceae:Alteromonadales:Gammaproteobacteria:Proteobacteria:Bacteria |
| Candidatus_Cloacamonas_acidaminovorans | NS_000195.1 | Candidatus Cloacamonas acidaminovorans:Candidatus Cloacamonas:candidate division WWE1:Bacteria |
| Staphylococcus_aureus_RF122 | NC_007622.1 | Staphylococcus aureus RF122:Staphylococcus:Bacillales:Firmicutes:Bacteria |
| Rhizobium_etli_CIAT_652 | NC_010994.1 | Rhizobium etli CIAT 652:Rhizobium:Rhizobium/Agrobacterium group:Rhizobiaceae:Rhizobiales:Alphaproteobacteria:Proteobacteria:Bacteria |
| Clavibacter_michiganensis_subsp_sepedonicus | NC_010407.1 | Clavibacter michiganensis subsp. sepedonicus:Clavibacter:Microbacteriaceae:Micrococcineae:Actinomycetales:Actinobacteridae:Actinobacteria:Bacteria |
| Burkholderia_mallei_NCTC_10229 | NC_008836.1 | Burkholderia mallei NCTC 10229:pseudomallei group:Burkholderia:Burkholderiaceae:Burkholderiales:Betaproteobacteria:Proteobacteria:Bacteria |
| Megasphaera_elsdenii | NC_015873.1 | Megasphaera elsdenii DSM 20460:Megasphaera:Veillonellaceae:Selenomonadales:Negativicutes:Firmicutes:Bacteria |
| Nitrosococcus_halophilus_Nc4 | NC_013960.1 | Nitrosococcus halophilus Nc4:Nitrosococcus:Chromatiaceae:Chromatiales:Gammaproteobacteria:Proteobacteria:Bacteria |
| Maribacter_sp_HTCC2170 | NC_014472.1 | Maribacter sp. HTCC2170:Maribacter:Flavobacteriaceae:Flavobacteriales:Flavobacteriia:Bacteroidetes:Bacteria |
| Candidatus_Arthromitus_sp_SFB_mouse_Yit | NC_017294.1 | Candidatus Arthromitus sp. SFB-mouse-Yit:Candidatus Arthromitus:Clostridiaceae:Clostridiales:Clostridia:Firmicutes:Bacteria |
| Thermanaerovibrio_acidaminovorans_DSM_6589 | NC_013522.1 | Thermanaerovibrio acidaminovorans DSM 6589:Thermanaerovibrio:Synergistaceae:Synergistales:Synergistia:Synergistetes:Bacteria |
| Salinibacter_ruber_DSM_13855 | NC_007677.1 | Salinibacter ruber DSM 13855:Salinibacter:Rhodothermaceae:Incertae sedis:Bacteroidetes Order II:Bacteroidetes:Bacteria |
| Syntrophobotulus_glycolicus_DSM_8271 | NC_015172.1 | Syntrophobotulus glycolicus DSM 8271:Syntrophobotulus:Peptococcaceae:Clostridiales:Clostridia:Firmicutes:Bacteria |
| Bacteroides_helcogenes_P_36_108 | NC_014933.1 | Bacteroides helcogenes P 36-108:Bacteroides:Bacteroidaceae:Bacteroidales:Bacteroidia:Bacteroidetes:Bacteria |
| Aeromonas_hydrophila_subsp_hydrophila_ATCC_7966 | NC_008570.1 | Aeromonas hydrophila subsp. hydrophila ATCC 7966:Aeromonas:Aeromonadaceae:Aeromonadales:Gammaproteobacteria:Proteobacteria:Bacteria |
| Alkalilimnicola_ehrlichii_MLHE_1 | NC_008340.1 | Alkalilimnicola ehrlichii MLHE-1:Alkalilimnicola:Ectothiorhodospiraceae:Chromatiales:Gammaproteobacteria:Proteobacteria:Bacteria |
| Bradyrhizobium_japonicum_USDA_110 | NC_004463.1 | Bradyrhizobium japonicum USDA 110:Bradyrhizobium:Bradyrhizobiaceae:Rhizobiales:Alphaproteobacteria:Proteobacteria:Bacteria |
| Shewanella_violacea_DSS12 | NC_014012.1 | Shewanella violacea DSS12:Shewanella:Shewanellaceae:Alteromonadales:Gammaproteobacteria:Proteobacteria:Bacteria |
| Pelodictyon_phaeoclathratiforme_BU_1 | NC_011060.1 | Pelodictyon phaeoclathratiforme BU-1:Pelodictyon:Chlorobium/Pelodictyon group:Chlorobiaceae:Chlorobiales:Chlorobia:Chlorobi:Bacteria |
| Acholeplasma_laidlawii_PG_8A | NC_010163.1 | Acholeplasma laidlawii PG-8A:Acholeplasma:Acholeplasmataceae:Acholeplasmatales:Mollicutes:Tenericutes:Bacteria |
| Hyphomicrobium_sp | NC_015717.1 | Hyphomicrobium sp. MC1:Hyphomicrobium:Hyphomicrobiaceae:Rhizobiales:Alphaproteobacteria:Proteobacteria:Bacteria |
| Alteromonas_macleodii_str_Deep_ecotype | NC_011138.2 | Alteromonas macleodii str. 'Deep ecotype':Alteromonas:Alteromonadaceae:Alteromonadales:Gammaproteobacteria:Proteobacteria:Bacteria |
| Bradyrhizobium_sp_BTAi1 | NC_009485.1 | Bradyrhizobium sp. BTAi1:Bradyrhizobium:Bradyrhizobiaceae:Rhizobiales:Alphaproteobacteria:Proteobacteria:Bacteria |
| Acinetobacter_baumannii_1656_2 | NC_017162.1 | Acinetobacter baumannii 1656-2:Acinetobacter calcoaceticus/baumannii complex:Acinetobacter:Moraxellaceae:Pseudomonadales:Gammaproteobacteria:Proteobacteria:Bacteria |
| Aeromonas_veronii_B565 | NC_015424.1 | Aeromonas veronii B565:Aeromonas:Aeromonadaceae:Aeromonadales:Gammaproteobacteria:Proteobacteria:Bacteria |
| Rickettsia_africae_ESF_5 | NC_012633.1 | Rickettsia africae ESF-5:spotted fever group:Rickettsia:Rickettsieae:Rickettsiaceae:Rickettsiales:Alphaproteobacteria:Proteobacteria:Bacteria |
| Variovorax_paradoxus_EPS | NC_014931.1 | Variovorax paradoxus EPS:Variovorax:Comamonadaceae:Burkholderiales:Betaproteobacteria:Proteobacteria:Bacteria |
| Delftia_sp_Cs1_4 | NC_015563.1 | Delftia sp. Cs1-4:Delftia:Comamonadaceae:Burkholderiales:Betaproteobacteria:Proteobacteria:Bacteria |
| Magnetococcus_sp_MC_1 | NC_008576.1 | Magnetococcus marinus MC-1:Magnetococcus:Magnetococcaceae:Magnetococcales:Alphaproteobacteria:Proteobacteria:Bacteria |
| Capnocytophaga_canimorsus_Cc5 | NC_015846.1 | Capnocytophaga canimorsus Cc5:Capnocytophaga:Flavobacteriaceae:Flavobacteriales:Flavobacteriia:Bacteroidetes:Bacteria |
| Clostridium_kluyveri_DSM_555 | NC_009706.1 | Clostridium kluyveri DSM 555:Clostridium:Clostridiaceae:Clostridiales:Clostridia:Firmicutes:Bacteria |
| Croceibacter_atlanticus_HTCC2559 | NC_014230.1 | Croceibacter atlanticus HTCC2559:Croceibacter:Flavobacteriaceae:Flavobacteriales:Flavobacteriia:Bacteroidetes:Bacteria |
| Lactobacillus_buchneri_NRRL_B_30929 | NC_015428.1 | Lactobacillus buchneri NRRL B-30929:Lactobacillus:Lactobacillaceae:Lactobacillales:Firmicutes:Bacteria |
| Methylobacterium_populi_BJ001 | NC_010725.1 | Methylobacterium populi BJ001:Methylobacterium:Methylobacteriaceae:Rhizobiales:Alphaproteobacteria:Proteobacteria:Bacteria |
| Kangiella_koreensis_DSM_16069 | NC_013166.1 | Kangiella koreensis DSM 16069:Kangiella:Alcanivoracaceae:Oceanospirillales:Gammaproteobacteria:Proteobacteria:Bacteria |
| Roseburia_hominis_A2_183 | NC_015977.1 | Roseburia hominis A2-183:Roseburia:Lachnospiraceae:Clostridiales:Clostridia:Firmicutes:Bacteria |
| Lactobacillus_sanfranciscensis_TMW_1.1304 | NC_015978.1 | Lactobacillus sanfranciscensis TMW 1.1304:Lactobacillus:Lactobacillaceae:Lactobacillales:Firmicutes:Bacteria |
| Psychrobacter_cryohalolentis_K5 | NC_007969.1 | Psychrobacter cryohalolentis K5:Psychrobacter:Moraxellaceae:Pseudomonadales:Gammaproteobacteria:Proteobacteria:Bacteria |
| Eggerthella_sp_YY7918 | NC_015738.1 | Eggerthella sp. YY7918:Eggerthella:Coriobacteriaceae:Coriobacterineae:Coriobacteriales:Coriobacteridae:Actinobacteria:Bacteria |
| Lactobacillus_amylovorus_GRL1118 | NC_017470.1 | Lactobacillus amylovorus GRL1118:Lactobacillus:Lactobacillaceae:Lactobacillales:Firmicutes:Bacteria |
| Acinetobacter_sp_ADP1 | NC_005966.1 | Acinetobacter sp. ADP1:Acinetobacter:Moraxellaceae:Pseudomonadales:Gammaproteobacteria:Proteobacteria:Bacteria |
| Gluconacetobacter_xylinus_NBRC_3288 | NC_016027.1 | Gluconacetobacter xylinus NBRC 3288:Gluconacetobacter:Acetobacteraceae:Rhodospirillales:Alphaproteobacteria:Proteobacteria:Bacteria |
| Desulfitobacterium_hafniense_Y51 | NC_007907.1 | Desulfitobacterium hafniense Y51:Desulfitobacterium:Peptococcaceae:Clostridiales:Clostridia:Firmicutes:Bacteria |
| Clostridium_botulinum_A_str_ATCC_3502 | NC_009495.1 | Clostridium botulinum A str. ATCC 3502:Clostridium:Clostridiaceae:Clostridiales:Clostridia:Firmicutes:Bacteria |
| Prevotella_ruminicola_23 | NC_014033.1 | Prevotella ruminicola 23:Prevotella:Prevotellaceae:Bacteroidales:Bacteroidia:Bacteroidetes:Bacteria |
| Lactobacillus_acidophilus_30SC | NC_015214.1 | Lactobacillus acidophilus 30SC:Lactobacillus:Lactobacillaceae:Lactobacillales:Firmicutes:Bacteria |
| Corynebacterium_diphtheriae_241 | NC_016782.1 | Corynebacterium diphtheriae 241:Corynebacterium:Corynebacteriaceae:Corynebacterineae:Actinomycetales:Actinobacteridae:Actinobacteria:Bacteria |
| Thermocrinis_albus_DSM_14484 | NC_013894.1 | Thermocrinis albus DSM 14484:Thermocrinis:Aquificaceae:Aquificales:Aquificae:Bacteria |
| Ruegeria_sp_TM1040 | NC_008044.1 | Ruegeria sp. TM1040:Ruegeria:Rhodobacteraceae:Rhodobacterales:Alphaproteobacteria:Proteobacteria:Bacteria |
| Polaromonas_naphthalenivorans_CJ2 | NC_008781.1 | Polaromonas naphthalenivorans CJ2:Polaromonas:Comamonadaceae:Burkholderiales:Betaproteobacteria:Proteobacteria:Bacteria |
| Planctomyces_limnophilus_DSM_3776 | NC_014148.1 | Planctomyces limnophilus DSM 3776:Planctomyces:Planctomycetaceae:Planctomycetales:Planctomycetia:Planctomycetes:Bacteria |
| Zymomonas_mobilis_subsp_mobilis_ZM4 | NC_006526.2 | Zymomonas mobilis subsp. mobilis ZM4:Zymomonas:Sphingomonadaceae:Sphingomonadales:Alphaproteobacteria:Proteobacteria:Bacteria |
| Herpetosiphon_aurantiacus_DSM_785 | NC_009972.1 | Herpetosiphon aurantiacus DSM 785:Herpetosiphon:Herpetosiphonaceae:Herpetosiphonales:Chloroflexi:Bacteria |
| Pelotomaculum_thermopropionicum_SI | NC_009454.1 | Pelotomaculum thermopropionicum SI:Pelotomaculum:Peptococcaceae:Clostridiales:Clostridia:Firmicutes:Bacteria |
| Bacillus_subtilis_subsp_spizizenii_str_W23 | NC_014479.1 | Bacillus subtilis subsp. spizizenii str. W23:Bacillus:Bacillaceae:Bacillales:Firmicutes:Bacteria |
| Xanthomonas_campestris_pv_campestris_str_ATCC_33913 | NC_003902.1 | Xanthomonas campestris pv. campestris str. ATCC 33913:Xanthomonas:Xanthomonadaceae:Xanthomonadales:Gammaproteobacteria:Proteobacteria:Bacteria |
| Mycobacterium_rhodesiae_NBB3 | NC_016604.1 | Mycobacterium rhodesiae NBB3:Mycobacterium:Mycobacteriaceae:Corynebacterineae:Actinomycetales:Actinobacteridae:Actinobacteria:Bacteria |
| Pectobacterium_wasabiae_WPP163 | NC_013421.1 | Pectobacterium wasabiae WPP163:Pectobacterium:Enterobacteriaceae:Enterobacteriales:Gammaproteobacteria:Proteobacteria:Bacteria |
| Coriobacterium_glomerans_PW2 | NC_015389.1 | Coriobacterium glomerans PW2:Coriobacterium:Coriobacteriaceae:Coriobacterineae:Coriobacteriales:Coriobacteridae:Actinobacteria:Bacteria |
| Rickettsia_bellii_RML369_C | NC_007940.1 | Rickettsia bellii RML369-C:belli group:Rickettsia:Rickettsieae:Rickettsiaceae:Rickettsiales:Alphaproteobacteria:Proteobacteria:Bacteria |
| Staphylococcus_epidermidis_ATCC_12228 | NC_004461.1 | Staphylococcus epidermidis ATCC 12228:Staphylococcus:Bacillales:Firmicutes:Bacteria |
| Pedobacter_saltans_DSM_12145 | NC_015177.1 | Pedobacter saltans DSM 12145:Pedobacter:Sphingobacteriaceae:Sphingobacteriales:Sphingobacteriia:Bacteroidetes:Bacteria |
| Clostridium_sp_BNL1100 | NC_016791.1 | Clostridium sp. BNL1100:Clostridium:Clostridiaceae:Clostridiales:Clostridia:Firmicutes:Bacteria |
| Bordetella_pertussis_CS | NC_017223.1 | Bordetella pertussis CS:Bordetella:Alcaligenaceae:Burkholderiales:Betaproteobacteria:Proteobacteria:Bacteria |
| Nakamurella_multipartita_DSM_44233 | NC_013235.1 | Nakamurella multipartita DSM 44233:Nakamurella:Nakamurellaceae:Frankineae:Actinomycetales:Actinobacteridae:Actinobacteria:Bacteria |
| Anaplasma_marginale_str_St_Maries | NC_004842.2 | Anaplasma marginale str. St. Maries:Anaplasma:Anaplasmataceae:Rickettsiales:Alphaproteobacteria:Proteobacteria:Bacteria |
| Rickettsia_slovaca_str_D_CWPP | NC_017065.1 | Rickettsia slovaca str. D-CWPP:spotted fever group:Rickettsia:Rickettsieae:Rickettsiaceae:Rickettsiales:Alphaproteobacteria:Proteobacteria:Bacteria |
| Desulfovibrio_africanus_str_Walvis_Bay | NC_016629.1 | Desulfovibrio africanus str. Walvis Bay:Desulfovibrio:Desulfovibrionaceae:Desulfovibrionales:Deltaproteobacteria:Proteobacteria:Bacteria |
| Anaerococcus_prevotii_DSM_20548 | NC_013171.1 | Anaerococcus prevotii DSM 20548:Anaerococcus:Incertae Sedis:Clostridiales Family XI:Clostridiales:Clostridia:Firmicutes:Bacteria |
| Thermodesulfobacterium_sp_OPB45 | NC_015682.1 | Thermodesulfobacterium sp. OPB45:Thermodesulfobacterium:Thermodesulfobacteriaceae:Thermodesulfobacteriales:Thermodesulfobacteria:Bacteria |
| Alcanivorax_borkumensis_SK2 | NC_008260.1 | Alcanivorax borkumensis SK2:Alcanivorax:Alcanivoracaceae:Oceanospirillales:Gammaproteobacteria:Proteobacteria:Bacteria |
| Marinobacter_aquaeolei_VT8 | NC_008740.1 | Marinobacter aquaeolei VT8:Marinobacter:Alteromonadaceae:Alteromonadales:Gammaproteobacteria:Proteobacteria:Bacteria |
| Desulfovibrio_desulfuricans_subsp_desulfuricans_str_ATCC_27774 | NC_011883.1 | Desulfovibrio desulfuricans subsp. desulfuricans str. ATCC 27774:Desulfovibrio:Desulfovibrionaceae:Desulfovibrionales:Deltaproteobacteria:Proteobacteria:Bacteria |
| Edwardsiella_ictaluri_93_146 | NC_012779.1 | Edwardsiella ictaluri 93-146:Edwardsiella:Enterobacteriaceae:Enterobacteriales:Gammaproteobacteria:Proteobacteria:Bacteria |
| Thermobispora_bispora_DSM_43833 | NC_014165.1 | Thermobispora bispora DSM 43833:Thermobispora:Pseudonocardiaceae:Pseudonocardineae:Actinomycetales:Actinobacteridae:Actinobacteria:Bacteria |
| Saccharopolyspora_erythraea_NRRL_2338 | NC_009142.1 | Saccharopolyspora erythraea NRRL 2338:Saccharopolyspora:Pseudonocardiaceae:Pseudonocardineae:Actinomycetales:Actinobacteridae:Actinobacteria:Bacteria |
| Geodermatophilus_obscurus_DSM_43160 | NC_013757.1 | Geodermatophilus obscurus DSM 43160:Geodermatophilus:Geodermatophilaceae:Frankineae:Actinomycetales:Actinobacteridae:Actinobacteria:Bacteria |
| Rickettsia_massiliae_str_AZT80 | NC_016931.1 | Rickettsia massiliae str. AZT80:spotted fever group:Rickettsia:Rickettsieae:Rickettsiaceae:Rickettsiales:Alphaproteobacteria:Proteobacteria:Bacteria |
| Nostoc_azollae_0708 | NC_014248.1 | 'Nostoc azollae' 0708:Trichormus:Nostocaceae:Nostocales:Cyanobacteria:Bacteria |
| Fusobacterium_nucleatum_subsp_nucleatum_ATCC_25586 | NC_003454.1 | Fusobacterium nucleatum subsp. nucleatum ATCC 25586:Fusobacterium:Fusobacteriaceae:Fusobacteriales:Fusobacteria:Bacteria |
| Sodalis_glossinidius_str_morsitans | NC_007712.1 | Sodalis glossinidius str. 'morsitans':Sodalis:Enterobacteriaceae:Enterobacteriales:Gammaproteobacteria:Proteobacteria:Bacteria |
| Bacillus_subtilis_BSn5 | NC_014976.1 | Bacillus subtilis BSn5:Bacillus:Bacillaceae:Bacillales:Firmicutes:Bacteria |
| Acidaminococcus_intestini_RyC_MR95 | NC_016077.1 | Acidaminococcus intestini RyC-MR95:Acidaminococcus:Acidaminococcaceae:Selenomonadales:Negativicutes:Firmicutes:Bacteria |
| Propionibacterium_acnes_ATCC_11828 | NC_017550.1 | Propionibacterium acnes ATCC 11828:Propionibacterium:Propionibacteriaceae:Propionibacterineae:Actinomycetales:Actinobacteridae:Actinobacteria:Bacteria |
| Rickettsia_peacockii_str_Rustic | NC_012730.1 | Rickettsia peacockii str. Rustic:spotted fever group:Rickettsia:Rickettsieae:Rickettsiaceae:Rickettsiales:Alphaproteobacteria:Proteobacteria:Bacteria |
| Gardnerella_vaginalis_409_05 | NC_013721.1 | Gardnerella vaginalis 409-05:Gardnerella:Bifidobacteriaceae:Bifidobacteriales:Actinobacteridae:Actinobacteria:Bacteria |
| Ketogulonicigenium_vulgare_Y25 | NC_014625.1 | Ketogulonicigenium vulgare Y25:Ketogulonicigenium:Rhodobacteraceae:Rhodobacterales:Alphaproteobacteria:Proteobacteria:Bacteria |
| Caulobacter_segnis_ATCC_21756 | NC_014100.1 | Caulobacter segnis ATCC 21756:Caulobacter:Caulobacteraceae:Caulobacterales:Alphaproteobacteria:Proteobacteria:Bacteria |
| Shewanella_woodyi_ATCC_51908 | NC_010506.1 | Shewanella woodyi ATCC 51908:Shewanella:Shewanellaceae:Alteromonadales:Gammaproteobacteria:Proteobacteria:Bacteria |
| Haemophilus_ducreyi_35000HP | NC_002940.2 | Haemophilus ducreyi 35000HP:Haemophilus:Pasteurellaceae:Pasteurellales:Gammaproteobacteria:Proteobacteria:Bacteria |
| Rickettsia_rickettsii_str_Brazil | NC_016913.1 | Rickettsia rickettsii str. Brazil:spotted fever group:Rickettsia:Rickettsieae:Rickettsiaceae:Rickettsiales:Alphaproteobacteria:Proteobacteria:Bacteria |
| Geobacter_bemidjiensis_Bem | NC_011146.1 | Geobacter bemidjiensis Bem:Geobacter:Geobacteraceae:Desulfuromonadales:Deltaproteobacteria:Proteobacteria:Bacteria |
| Pseudomonas_putida_BIRD_1 | NC_017530.1 | Pseudomonas putida BIRD-1:Pseudomonas:Pseudomonadaceae:Pseudomonadales:Gammaproteobacteria:Proteobacteria:Bacteria |
| Bacillus_licheniformis_DSM_13 | NC_006322.1 | Bacillus licheniformis DSM 13 = ATCC 14580:Bacillus:Bacillaceae:Bacillales:Firmicutes:Bacteria |
| Yersinia_pestis_A1122 | NC_017168.1 | Yersinia pestis A1122:Yersinia:Enterobacteriaceae:Enterobacteriales:Gammaproteobacteria:Proteobacteria:Bacteria |
| Arthrobacter_chlorophenolicus_A6 | NC_011886.1 | Arthrobacter chlorophenolicus A6:Arthrobacter:Micrococcaceae:Micrococcineae:Actinomycetales:Actinobacteridae:Actinobacteria:Bacteria |
| Methylobacillus_flagellatus_KT | NC_007947.1 | Methylobacillus flagellatus KT:Methylobacillus:Methylophilaceae:Methylophilales:Betaproteobacteria:Proteobacteria:Bacteria |
| Bacillus_megaterium_QM_B1551 | NC_014019.1 | Bacillus megaterium QM B1551:Bacillus:Bacillaceae:Bacillales:Firmicutes:Bacteria |
| Streptomyces_flavogriseus_ATCC_33331 | NC_016114.1 | Streptomyces flavogriseus ATCC 33331:Streptomyces:Streptomycetaceae:Streptomycineae:Actinomycetales:Actinobacteridae:Actinobacteria:Bacteria |
| Pantoea_ananatis_PA13 | NC_017554.1 | Pantoea ananatis PA13:Pantoea:Enterobacteriaceae:Enterobacteriales:Gammaproteobacteria:Proteobacteria:Bacteria |
| Desulfurispirillum_indicum_S5 | NC_014836.1 | Desulfurispirillum indicum S5:Desulfurispirillum:Chrysiogenaceae:Chrysiogenales:Chrysiogenetes:Bacteria |
| Nocardia_farcinica_IFM_10152 | NC_006361.1 | Nocardia farcinica IFM 10152:Nocardia:Nocardiaceae:Corynebacterineae:Actinomycetales:Actinobacteridae:Actinobacteria:Bacteria |
| Pseudomonas_syringae_pv_syringae_B728a | NC_007005.1 | Pseudomonas syringae pv. syringae B728a:Pseudomonas syringae:Pseudomonas:Pseudomonadaceae:Pseudomonadales:Gammaproteobacteria:Proteobacteria:Bacteria |
| Cronobacter_turicensis_z3032 | NC_013282.2 | Cronobacter turicensis z3032:Cronobacter:Enterobacteriaceae:Enterobacteriales:Gammaproteobacteria:Proteobacteria:Bacteria |
| Pseudoalteromonas_sp_SM9913 | NC_014803.1 | Pseudoalteromonas sp. SM9913:Pseudoalteromonas:Pseudoalteromonadaceae:Alteromonadales:Gammaproteobacteria:Proteobacteria:Bacteria |
| Geobacillus_sp_C56_T3 | NC_014206.1 | Geobacillus sp. C56-T3:Geobacillus:Bacillaceae:Bacillales:Firmicutes:Bacteria |
