# Supplementary material for: Brood Ball-Mediated Transmission of Microbiome Members in the Dung Beetle, Onthophagus taurus (Coleoptera: Scarabaeidae)
Source: PLoS One. 2013 Nov 1;8(11):e79061. doi: 10.1371/journal.pone.0079061 (PMC3815100; doi:10.1371/journal.pone.0079061)
Supplement: Table S2 — Abbreviations and full names of the loci used for the phylotyping analysis. (DOCX) [file pone.0079061.s006.docx]

**Locus Abbreviation Locus Description**

| acpP | acyl carrier protein |
| --- | --- |
| acpS | holo-(acyl-carrier-protein) synthase |
| adk | adenylate kinase |
| alaS | putative alanyl-tRNA synthetase |
| apt | adenine phosphoribosyltransferase |
| argB | acetylglutamate kinase |
| argC | N-acetyl-gamma-glutamyl-phosphate reductase |
| argG | putative argininosuccinate synthase |
| argS | putative arginyl-tRNA synthetase |
| aroC | chorismate synthase |
| aroE | shikimate 5-dehydrogenase |
| asd | aspartate semialdehyde dehydrogenase |
| aspS | aspartyl-tRNA synthetase |
| atpB | ATP synthase subunit a |
| atpC | ATP synthase subunit epsilon |
| atpD | ATP synthase subunit beta |
| atpE | ATP synthase subunit c |
| atpF | putative ATP synthase subunit b |
| atpG | ATP synthase subunit gamma |
| atpH | putative ATP synthase delta chain |
| bioB | putative biotin synthase |
| birA | BirA bifunctional biotin operon repressor/biotin--[acetyl-CoA-carboxylase synthetase |
| carB | carbamoyl phosphate synthase large subunit |
| cdsA | phosphatidate cytidylyltransferase |
| cmk | cytidylate kinase |
| coaBC | phosphopantothenoylcysteine decarboxylase /phosphopantothenate--cysteine ligase |
| coaD | putative phosphopantetheine adenylyltransferase |
| coaE | putative dephospho-CoA kinase |
| dapB | dihydrodipicolinate reductase |
| dapD | putative tetrahydrodipicolinate acetyltransferase |
| dapF | diaminopimelate epimerase |
| dnaA | chromosomal replication initiator protein DnaA |
| dnaG | DNA primase |
| dnaN | DNA polymerase III subunit beta |
| dnaX | DNA polymerase III subunit tau |
| dtd | D-tyrosyl-tRNA(Tyr) deacylase |
| dxr | 1-deoxy-D-xylulose 5-phosphate reductoisomerase |
| efp | putative elongation factor P |
| engB | ribosome biogenesis GTP-binding protein YsxC |
| era | putative GTP-binding protein |
| fabZ | putative hydroxymyristoyl-(acyl carrier protein)dehydratase |
| fmt | autolysis and methicillin resistant-related protein |
| folC | putative folylpolyglutamate synthase |
| folK | putative 2-amino-4-hydroxy-6-hydroxymethyldihydropteridine pyrophosphokinase |
| frr | ribosome recycling factor |
| ftsA | putative cell division protein |
| fusA | elongation factor G |
| gatA | glutamyl-tRNA amidotransferase subunit A |
| gatB | glutamyl-tRNA amidotransferase subunit B |
| gatC | glutamyl-tRNA amidotransferase subunit C |
| gcp | putative O-sialoglycoprotein endopeptidase |
| gidA | glucose inhibited division protein A |
| gidB | putative glucose inhibited division protein B |
| glmM | phosphoglucosamine mutase |
| glmU | UDP-N-acetylglucosamine pyrophosphorylase |
| glyS | glycyl-tRNA synthetase |
| gmk | guanylate kinase |
| gpsA | glycerol-3-phosphate dehydrogenase [NAD(P)+] |
| groEL | 60 kDa chaperonin |
| grpE | GrpE protein (Hsp-70 cofactor) |
| guaA | putative GMP synthase |
| guaB | putative inosine-5'-monophosphate dehydrogenase |
| gyrA | DNA gyrase subunit A |
| hemA | glutamyl-tRNA reductase |
| hemB | delta-aminolevulinic acid dehydratase |
| hemC | porphobilinogen deaminase |
| hemE | uroporphyrinogen decarboxylase |
| hemH | ferrochelatase |
| hemK | peptide release factor-glutamine N5-methyltransferase |
| hemN | coproporphyrinogen III oxidase |
| hisA | 1-(5-phosphoribosyl)-5-[(5- phosphoribosylamino)methylideneamino]imidazole-4- carboxamide isomerase |
| hisB | imidazoleglycerol-phosphate dehydratase |
| hisG | ATP phosphoribosyltransferase |
| hisS | histidyl-tRNA synthetase |
| holA | DNA polymerase III subunit delta |
| hslR | heat shock protein 15-like protein |
| hslV | putative ATP-dependent protease |
| ilvC | ketol-acid reductoisomerase |
| infB | translation initiation factor IF-2 |
| infC | translation initiation factor IF-3 |
| ipk | 4-diphosphocytidyl-2-C-methyl-D-erythritol kinase |
| ispF | 2-C-methyl-D-erythritol 2,4-cyclodiphosphate synthase |
| ispG | 4-hydroxy-3-methylbut-2-en-1-yl diphosphate synthase |
| ksgA | dimethyladenosine transferase |
| lepA | putative GTP-binding protein |
| leuS | leucyl-tRNA synthetase |
| lgt | prolipoprotein diacylglyceryl transferase |
| ligA | DNA ligase |
| lspA | lipoprotein signal peptidase |
| maf | Maf-like protein |
| metF | 5,10-methylenetetrahydrofolate reductase |
| metG | putative methionyl-tRNA synthetase |
| miaA | putative tRNA delta 2-isopentenyl pyrophosphatetransferase |
| mraW | S-adenosyl-L-methionine-dependent methyltransferas |
| mraY | phospho-N-acetylmuramoyl-pentapeptide- transferase |
| mraZ | cell division protein MraZ |
| mreC | cell shape determining protein |
| murB | putative UDP-N-acetylenolpyruvoylglucosamine reductase |
| murD | UDP-N-acetylmuramoylalanine--D-glutamate ligase |
| murG | putative UDP-N-acetylglucosamine-N-acetylmuramyl- (pentapeptide)pyrophosphoryl-undecaprenol N-acetylglucosamine transferase |
| murI | putative glutamate racemase |
| mutL | DNA mismatch repair protein MutL |
| mutS | DNA mismatch repair protein MutS |
| mutY | MutY A/G-specific adenine DNA glycosylase |
| nadA | Quinolinate synthetase A |
| nadD | putative nitrile hydratase subunit alpha |
| nadE | NAD synthetase |
| nusA | putative N utilization substance protein A |
| nusB | transcription termination factor N-utilization substance protein B |
| nusG | transcription antitermination protein |
| obgE | GTPase ObgE |
| pcnB | poly(A) polymerase / tRNA nucleotidyltransferase |
| pgi | glucose-6-phosphate isomerase |
| pgk | phosphoglycerate kinase |
| pgsA | putative CDP-diacylglycerol--glycerol-3-phosphate 3-phosphatidyltransferase |
| pheA | prephenate dehydratase |
| pheS | putative phenylalanyl-tRNA synthetase alpha chain |
| pheT | putative phenylalanyl-tRNA synthetase beta chain |
| plsX | glycerol-3-phosphate acyltransferase PlsX |
| pnp | polynucleotide phosphorylase |
| polA | DNA polymerase I |
| ppnK | inorganic polyphosphate/ATP-NAD kinase |
| prfB | peptide chain release factor 2 |
| priA | primosomal protein n' |
| prmA | ribosomal protein L11 methyltransferase |
| proA | gamma-glutamyl phosphate reductase |
| proB | glutamate 5-kinase |
| proS | prolyl-tRNA synthetase |
| prs | ribose-phosphate pyrophosphokinase |
| pth | peptidyl-tRNA hydrolase (PTH) |
| purA | putative adenylosuccinate synthetase |
| purC | putative phosphoribosylaminoimidazole-succinocarboxamide synthase |
| purE | putative phosphoribosylaminoimidazole carboxylase catalytic subunit |
| purF | putative amidophosphoribosyltransferase precursor |
| purH | bifunctional purine biosynthesis phosphoribosylaminoimidazolecarboxamide formyltransferase/IMP cyclohydrolase |
| purL | putative phosphoribosylformylglycinamidine synthase II |
| purM | putative phosphoribosylformylglycinamidine cyclo-ligase |
| purN | putative phosphoribosylglycinamide formyltransferase |
| purT | phosphoribosylglycinamide formyltransferase 2 |
| pyrD | dihydroorotate dehydrogenase 2 |
| pyrE | putative orotate phosphoribosyltransferase |
| pyrG | putative CTP synthase |
| pyrH | putative uridylate kinase |
| queA | S-adenosylmethionine:tRNA-ribosyltransferase- isomerase |
| radA | putative DNA repair protein |
| rbfA | putative ribosome-binding factor A |
| recA | recombinase A |
| recF | DNA replication and repair protein RecF |
| recJ | single-stranded-DNA-specific exonuclease RecJ |
| recN | DNA repair protein RecN |
| recO | DNA repair protein RecO |
| recR | putative recombination protein |
| ribD | bifunctional riboflavin biosynthesis protein |
| ribE | riboflavin synthase subunit alpha |
| ribF | riboflavin biosynthesis protein RibF |
| ribH | 6,7-dimethyl-8-ribityllumazine synthase |
| rimI | ribosomal-protein-S18-alanine acetyltransferase |
| rimM | 16S rRNA processing protein |
| rnc | ribonuclease III |
| rne | ribonuclease G |
| rnhA | ribonuclease H |
| rnhB | putative ribonuclease HII |
| rnpA | ribonuclease P protein component |
| rnr | putative ribonuclease R |
| rph | ribonuclease PH |
| rplA | 50S ribosomal protein L1 |
| rplB | 50S ribosomal protein L2 |
| rplC | 50S ribosomal protein L3 |
| rplD | 50S ribosomal protein L4 |
| rplE | 50S ribosomal protein L5 |
| rplF | 50S ribosomal protein L6 |
| rplI | 50S ribosomal protein L9 |
| rplJ | 50S ribosomal protein L10 |
| rplK | 50S ribosomal protein L11 |
| rplL | 50S ribosomal protein L7/L12 |
| rplM | 50S ribosomal protein L13 |
| rplN | 50S ribosomal protein L14 |
| rplO | 50S ribosomal protein L15 |
| rplP | 50S ribosomal protein L16 |
| rplQ | 50S ribosomal protein L17 |
| rplR | 50S ribosomal protein L18 |
| rplS | 50S ribosomal protein L19 |
| rplT | 50S ribosomal protein L20 |
| rplU | 50S ribosomal protein L21 |
| rplV | 50S ribosomal protein L22 |
| rplW | 50S ribosomal protein L23 |
| rplX | 50S ribosomal protein L24 |
| rplY | putative 50S ribosomal protein L25 |
| rpmA | 50S ribosomal protein L27 |
| rpmB | 50S ribosomal protein L28 |
| rpmC | 50S ribosomal protein L29 |
| rpmD | 50S ribosomal protein L30 |
| rpmE | putative 50S ribosomal protein L31 |
| rpmF | 50S ribosomal protein L32 |
| rpmG | 50S ribosomal protein L33 |
| rpmH | 50S ribosomal protein L34 |
| rpmI | 50S ribosomal protein L35 |
| rpoA | DNA-directed RNA polymerase subunit alpha |
| rpoB | DNA-directed RNA polymerase beta chain protein |
| rpoC | DNA-directed RNA polymerase beta' chain protein |
| rpoD | RNA polymerase sigma factor |
| rpoZ | DNA-directed RNA polymerase, omega subunit |
| rpsA | 30S ribosomal protein S1 |
| rpsB | 30S ribosomal protein S2 |
| rpsC | 30S ribosomal protein S3 |
| rpsD | 30S ribosomal protein S4 |
| rpsE | 30S ribosomal protein S5 |
| rpsF | 30S ribosomal protein S6 |
| rpsG | 30S ribosomal protein S7 |
| rpsH | 30S ribosomal protein S8 |
| rpsI | 30S ribosomal protein S9 |
| rpsJ | 30S ribosomal protein S10 |
| rpsK | 30S ribosomal protein S11 |
| rpsL | 30S ribosomal protein S12 |
| rpsM | 30S ribosomal protein S13 |
| rpsN | 30S ribosomal protein S14 |
| rpsO | 30S ribosomal protein S15 |
| rpsP | 30S ribosomal protein S16 |
| rpsQ | 30S ribosomal protein S17 |
| rpsR | 30S ribosomal protein S18 |
| rpsS | 30S ribosomal protein S19 |
| rpsT | putative 30S ribosomal protein S20 |
| ruvA | Holliday junction ATP-dependent DNA helicase RuvA |
| ruvB | holliday junction DNA helicase |
| ruvC | Holliday junction resolvase |
| scpB | segregation and condensation protein B |
| secG | putative protein-export membrane protein |
| secY | preprotein translocase subunit SecY |
| serC | phosphoserine aminotransferase |
| serS | seryl-tRNA synthetase |
| smpB | putative tmRNA-binding protein |
| spoT | pentaphosphate guanosine-3'-pyrophosphohydrolase |
| tatC | sec-independent protein translocase |
| tgt | queuine tRNA-ribosyltransferase |
| thiE | putative thiamine-phosphate pyrophosphorylase |
| thrB | homoserine kinase |
| thrS | threonyl-tRNA synthetase |
| tig | trigger factor (prolyl isomerase) |
| tmk | putative thymidylate kinase |
| topA | DNA topoisomerase I |
| trmD | putative tRNA (guanine-7-)-methyltransferase |
| trmE | tRNA modification GTPase TrmE |
| trpA | tryptophan synthase alpha chain |
| trpB | tryptophan synthase beta chain |
| trpC | indole-3-glycerol phosphate synthase |
| trpD | anthranilate phosphoribosyltransferase |
| trpE | anthranilate synthase component I |
| trpS | putative tryptophanyl-tRNA synthetase |
| truA | tRNA pseudouridine synthase A |
| truB | tRNA pseudouridine synthase B |
| tsf | elongation factor Ts |
| typA | GTP-binding protein TypA |
| tyrA | prephenate dehydrogenase |
| tyrS | tyrosyl-tRNA synthetase |
| ubiE | ubiquinone/menaquinone biosynthesis methlytransferase UbiE |
| upp | uracil phosphoribosyltransferase |
| uvrB | excinuclease ABC subunit B |
| uvrC | putative excinuclease ABC subunit C |
| valS | valyl-tRNA synthetase |
| xseA | exodeoxyribonuclease VII large subunit |
| xseB | exodeoxyribonuclease VII (small subunit) |
| yajC | preprotein translocase subunit YajC |
| ychF | catalase inhibitor protein |
| yidC | membrane protein insertase |
